# Synthesis of 2-Oxazolines from Ring Opening Isomerization of 3-Amido-2-Phenyl Azetidines

**DOI:** 10.3390/molecules26040857

**Published:** 2021-02-06

**Authors:** Xin Zhou, Baiyi Mao, Zhanbin Zhang

**Affiliations:** College of Chemistry, Beijing Normal University, Beijing 100875, China; 201321150129@mail.bnu.edu.cn (X.Z.); 201821150037@mail.bnu.edu.cn (B.M.)

**Keywords:** azetidine, oxazoline, isomerization, amide, acid

## Abstract

Chiral 2-oxazolines are valuable building blocks and famous ligands for asymmetric catalysis. The most common synthesis involves the reaction of an amino alcohol with a carboxylic acid. In this paper, an efficient synthesis of 2-oxazolines has been achieved via the stereospecific isomerization of 3-amido-2-phenyl azetidines. The reactions were studied in the presence of both Brønsted and Lewis acids, and Cu(OTf)_2_ was found to be the most effective.

## 1. Introduction

2-Oxazolines are very important five-membered heterocycles existing in numerous medicinally active compounds and natural products [1,2,3,4,5,6]. They are also widely applied as synthetic intermediates, protecting, activating and directing groups in organic synthesis. In addition, the optically active 2-oxazolines are valuable chiral building blocks and famous ligands for asymmetric catalysis [7,8,9,10,11]. There are numerous methods that have been developed for the preparation of 2-oxazolines [12,13,14,15]. The most common process for their preparation involves the reaction of an amino alcohol with a carboxylic acid [16,17]. In this way, oxazoline amines can be accessed from commercially available β-amino alcohols and suitably protected α-amino acids [18].

**Scheme 1:**
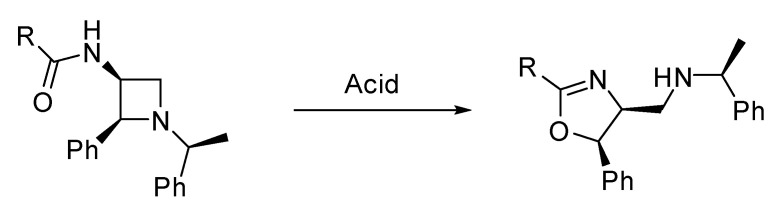
Isomerization of 3-amido-2-phenyl azetidines to 2-oxazolines.

Recently, we reported the synthesis of chiral 4,5-dihydrothiazol-2-amines and 4,5-dihydrooxazol-2-amines through an unexpected ring opening reaction of azetidines [19]. Based on this strategy, we now extend our research work and report herein the asymmetric synthesis of 2-oxazolines via the stereospecific isomerization of 3-amido-2-phenyl azetidines in the presence of acid (Scheme 1).

## 2. Results

**Scheme 2:**
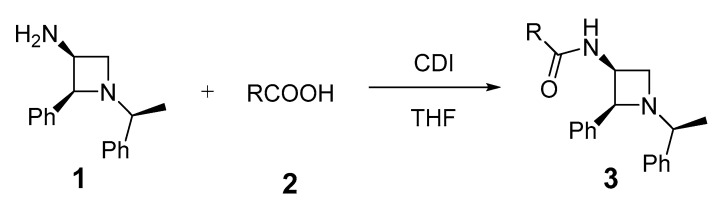
Synthesis of 3-amido-2-phenyl azetidines

Chiral 3-amino-2-phenyl azetidine **1** was prepared as previously reported by our group [20]. The coupling of 3-amino-2-phenyl azetidine **1** with acids **2** led to the corresponding amides **3,** which were obtained in 77% to 95% yields (Scheme 2; see Supplementary Materials).

**Scheme 3:**
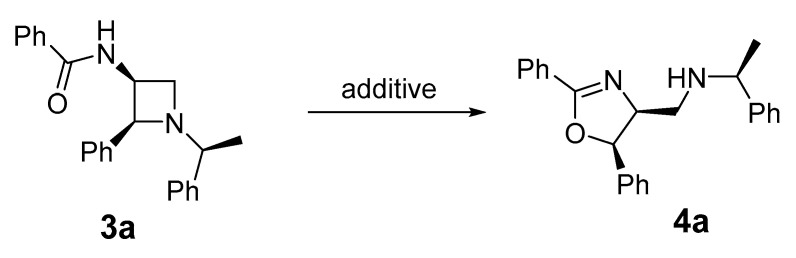
Isomerization of amide **3a** in different conditions.

The isomerization of amide **3a** was examined in different conditions (Scheme 3**, Table 1**). The results (Table 1) showed that the isomerization of **3a** did not occur at reflux in 1,2-dichloroethane (Table 1, entry 1) or in toluene (Table 1, entry 2) without additive or in the presence of bases such as DABCO, t-BuOK and NaH (Table 1, entries 3–5). Nevertheless, isomerization of amide **3a** to 2-oxazoline **4a** occurred in the presence of acids, including Lewis acids and Brønsted acids but excepting acetic acid (Table 1, entries 6–16). In search for a better additive, we explored Brønsted acids such as HClO_4_, CF_3_SO_3_H, CH_3_COOH and CF_3_COOH (TFA). It was found that TFA was the suitable additive for this transformation and the yield of **4a** could achieve up to 91% in 1,2-dichloroethane for about half an hour (Table 1, entry 10). However, prolonging the reaction time to over 2 h resulted in some degradation (Table 1, entry 11). Moreover, among the Lewis acids, Cu(OTf)_2_ turned out to be the most efficient for this transformation, allowing the formation of **4a** in a 93% yield (Table 1, entry 14).

**Scheme 4:**
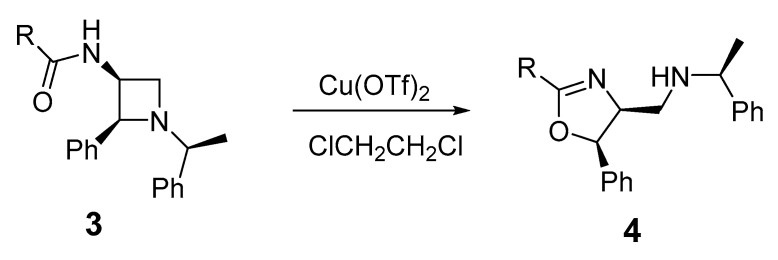
Substrate scope of the isomerization of amides **3**.

After optimizing the reaction conditions, we extended the substrate scope and different amides were examined (Scheme 4, Table 2). The amides with aryl, heteroaryl and alkyl groups were successfully isomerized to 2-oxazolines in the presence of Cu(OTf)_2_ in high yields (Table 2, entries 1–9 and 13–16). However, those substrates with 2-hydroxyl or 2-amino groups could not be isomerized, presumably due to their coordination to Cu(OTf)_2_. Nevertheless, these substrates could be isomerized to 2-oxazolines in the presence of CF_3_COOH in good yields (Table 2, entries 10–12).

**Scheme 5:**
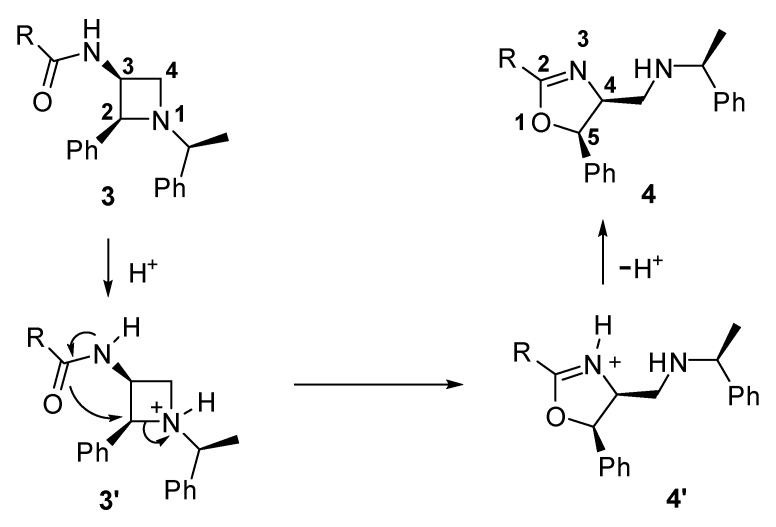
Proposed mechanism.

**Figure 1:**
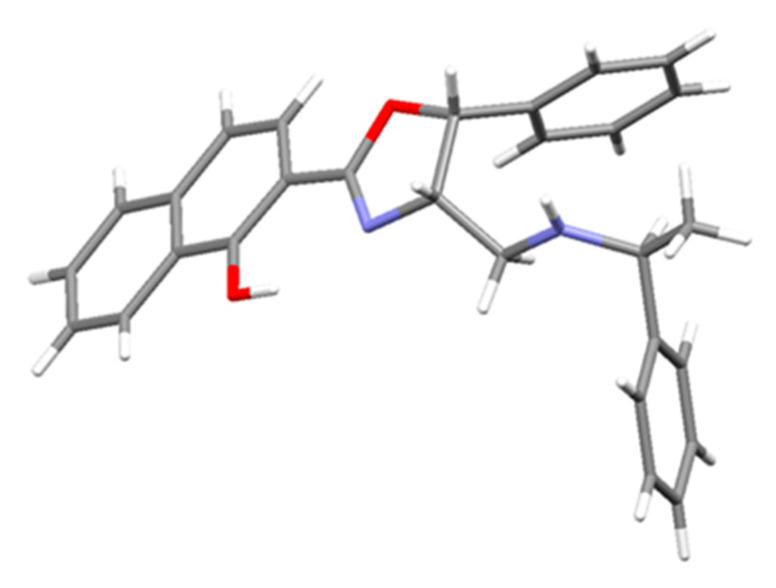
Structure of **4l**.

A proposed mechanism for this transformation is shown below (Scheme 5). The isomerization of amides **3** occurred regiospecifically by presumably an SN_2_ nucleophilic attack at the more active C2 but not the less-hindered C4 of the azetidine ring, thus the stereochemistry of 2-oxazolines **4** was shown to be *cis* [21]. This is also supported by comparison of the coupling constant (10 Hz) between H4 and H5 with reported *cis*-2-oxazolines [22,23]. In addition, all the structures of 2-oxazolines were established by ^1^H and ^13^C NMR, high resolution mass spectra (HRMS), IR. Furthermore, the absolute configuration of **4l** was further confirmed by X-ray analysis (Figure 1).

## 3. Materials and Methods

### 3.1. General Information

All reactants and reagents were commercially available and were used without further purification. ^1^H and ^13^C NMR spectra were recorded on a Bruker Advance III 400 MHz spectrometer (Billerica, MA, USA). Chemical shifts are reported in δ values (ppm) relative to an internal reference (0.03% *v*/*v*) of tetramethylsilane (TMS) for ^1^H NMR or the solvent signal, chloroform (CDCl_3_), for ^13^C NMR. IR data was obtained with an IRAffinity-1 spectrometer (Shimadzu, Kyoto, Japan). High resolution mass spectrometry (HRMS) was conducted with a high-resolution LCT Premier XE mass spectrometer in positive ESI mode (Waters, MA, USA). Melting points were measured on a digital melting point apparatus and are uncorrected.

### 3.2. General Procedure for the Isomeization of Amide 3

A mixture of amide **3** (1 mmol), Cu(OTf)_2_ (0.5 mmol) in 1,2-dichloroethane (10 mL) was refluxed for 4 h. The mixture was washed with water (10 mL), saturated NaHCO_3_ (10 mL), and dried over Na_2_SO_4_. The solvent was removed under reduced pressure. The residue was purified by gradient column chromatography on silica gel with PE/EA (5:1–2:1) as eluent to give oxazoline **4**.

(S)-N-(((4S,5R)-2,5-diphenyl-4,5-dihydrooxazol-4-yl)methyl)-1-phenylethanamine (**4a**)

Yellow oil, 96% yield, [α]_D_^20^ = −322.0 (c 1.0 CH_3_COOC_2_H_5_); ^1^H NMR (400 MHz, CDCl_3_) δ = 8.02 (d, *J* = 7.4 Hz, 2H), 7.59–7.00 (m, 13H), 5.79 (d, *J* = 9.9 Hz, 1H), 4.68 (q, *J* = 7.2 Hz, 1H), 3.46 (q, *J* = 6.4 Hz, 1H), 2.43–2.14 (m, 3H), 1.16 (d, *J* = 6.5 Hz, 3H); ^13^C NMR (100 MHz, CDCl_3_) δ = 23.72, 49.35, 58.29, 69.67, 83.13, 126.36, 126.49, 126.89, 127.53, 128.21, 128.35, 128.38, 128.42, 128.44, 131.60, 136.41, 144.86, 163.76; IR (KBr) ν 3061, 3028, 2963, 2924, 1651, 1603, 1580, 1495, 1450, 1368, 1333, 1277, 1246, 1209, 1175, 1128, 1082, 1065, 1024, 964, 781, 760, 696, 540 cm^−1^; HRMS *m/z* [M + H]^+^ calcd. for [C_24_H_25_N_2_O]^+^ 357.1961, found 357.1962.

(S)-N-(((4S,5R)-2-(4-methoxyphenyl)-5-phenyl-4,5-dihydrooxazol-4-yl)methyl)-1-phenylethanamine (**4b**)

Yellow solid, 94% yield, mp:71~74 °C, [α]_D_^20^ = −273.8 (c 1.0 CH_3_COOC_2_H_5_); ^1^H NMR (400 MHz, CDCl_3_) δ = 7.96 (d, *J* = 8.8 Hz, 2H), 7.37–7.07 (m, 10H), 6.92 (d, *J* = 8.9 Hz, 2H), 5.73 (d, *J* = 9.9 Hz, 1H), 4.61 (dt, *J* = 9.8, 7.0 Hz, 1H), 3.81 (s, 3H), 3.41 (q, *J* = 6.5 Hz, 1H), 2.28 (d, *J* = 7.0 Hz, 2H), 1.69 (br s, 1H), 1.12 (d, *J* = 6.6 Hz, 3H); ^13^C NMR (100 MHz, CDCl_3_) δ = 23.99, 49.55, 55.38, 58.23, 69.85, 83.10, 113.80, 120.08, 126.42, 126.46, 126.74, 128.12, 128.31, 128.33, 130.16, 136.69, 145.50, 162.28, 163.51; IR (KBr) ν 3061, 3026, 2961, 2930, 2837, 1647, 1609, 1512, 1495, 1452, 1420, 1368, 1335, 1310, 1256, 1167, 1076, 1030, 966, 841, 741, 700, 685 cm^−1^; HRMS *m/z* [M + H]^+^ calcd. for [C_25_H_27_N_2_O_2_]^+^ 387.2067, found 387.2067.

(S)-N-(((4S,5R)-2-(2-methoxyphenyl)-5-phenyl-4,5-dihydrooxazol-4-yl)methyl)-1-phenylethanamine (**4c**)

Yellow oil, 93% yield, [α]_D_^20^ = −204.5 (c 1.0 CH_3_COOC_2_H_5_); ^1^H NMR (400 MHz, CDCl_3_) δ = 7.80 (d, *J* = 7.2 Hz, 1H), 7.44 (t, *J* = 7.3 Hz, 1H), 7.39–7.06 (m, 10H), 6.98 (d, *J* = 7.9 Hz, 2H), 5.76 (d, *J* = 9.9 Hz, 1H), 4.80–4.74 (m, 1H), 3.90 (s, 4H), 3.52–3.45 (m, 1H), 2.39–2.27 (m, 2H), 1.21 (d, *J* = 6.1 Hz, 3H); ^13^C NMR (100 MHz, CDCl_3_) δ = 23.31, 49.09, 55.99, 58.38, 69.43, 82.44, 111.71, 116.89, 120.35, 126.45, 126.64, 127.10, 128.13, 128.32, 128.41, 131.40, 132.58, 136.43, 143.87, 158.61, 162.87; IR (KBr) ν 3061, 3028, 2961, 2924, 2837, 1653, 1636, 1578, 1560, 1491, 1458, 1437, 1331, 1283, 1258, 1123, 1043, 1024, 968, 754, 700, 592 cm^−1^; HRMS *m/z* [M + H]^+^ calcd. for [C_25_H_27_N_2_O_2_]^+^ 387.2067, found 387.2064.

(S)-N-(((4S,5R)-2-(4-nitrophenyl)-5-phenyl-4,5-dihydrooxazol-4-yl)methyl)-1-phenylethanamine (**4d**)

White solid, 94% yield, mp: 68~71 °C, [α]_D_^20^ = −145.8 (c 1.0 CH_3_COOC_2_H_5_); ^1^H NMR (400 MHz, CDCl_3_) δ = 8.29 (d, *J* = 8.9 Hz, 2H), 8.19 (d, *J* = 8.9 Hz, 2H), 7.42–7.30 (m, 3H), 7.28–7.11 (m, 7H), 5.85 (d, *J* = 10.1 Hz, 1H), 4.72 (dt, *J* = 10.0, 7.1 Hz, 1H), 3.45 (q, *J* = 6.5 Hz, 1H), 2.38–2.21 (m, 2H), 2.02 (br s, 1H), 1.16 (d, *J* = 6.6 Hz, 3H); ^13^C NMR (100 MHz, CDCl_3_) δ = 23.72, 49.16, 58.25, 70.20, 83.83, 123.62, 126.28, 126.41, 126.91, 128.38, 128.44, 128.46, 129.42, 133.31, 135.79, 144.99, 149.64, 161.93; IR (KBr) ν 3082, 3061, 3030, 2959, 2924, 2873, 1653, 1647, 1599, 1524, 1493, 1452, 1410, 1348, 1277, 1107, 1076, 1014, 961, 866, 851, 762, 702 cm^−1^; HRMS *m/z* [M + H]^+^ calcd. for [C_24_H_24_N_3_O_3_]^+^ 402.1812, found 402.1812.

(S)-N-(((4S,5R)-2-(2-nitrophenyl)-5-phenyl-4,5-dihydrooxazol-4-yl)methyl)-1-phenylethanamine (**4e**)

White solid, 91% yield, mp: 44~47 °C, [α]_D_^20^ = −314.7 (c 1.0 CH_3_COOC_2_H_5_); ^1^H NMR (400 MHz, CDCl_3_) δ = 7.90–7.79 (m, 2H), 7.68–7.53 (m, 2H), 7.39–7.06 (m, 10H), 5.80 (d, *J* = 10.0 Hz, 1H), 4.66 (dt, *J* = 9.8, 7.0 Hz, 1H), 3.44 (q, *J* = 6.5 Hz, 1H), 2.44–2.12 (m, 2H), 1.47 (br s, 1H), 1.12 (d, *J* = 6.6 Hz, 3H); ^13^C NMR (100 MHz, CDCl_3_) δ 24.00, 49.00, 58.11, 69.98, 84.57, 123.14, 123.96, 126.34, 126.42, 126.74, 128.28, 128.32, 128.40, 131.12, 131.61, 132.49, 135.43, 145.30, 149.13, 161.28; IR (KBr) ν 3327, 3063, 3028, 2963, 2926, 2833, 1661, 1607, 1576, 1537, 1493, 1450, 1368, 1352, 1279, 1244, 1209, 1107, 1059, 1032, 957, 852, 762, 736, 700, 681, 650 cm^−1^; HRMS *m/z* [M + H]^+^ calcd. for [C_24_H_24_N_3_O_3_]^+^ 402.1812, found 402.1812.

(S)-N-(((4S,5R)-2-(4-chlorophenyl)-5-phenyl-4,5-dihydrooxazol-4-yl)methyl)-1-phenylethanamine (**4f**)

Yellow oil, 79% yield, [α]_D_^20^ = −171.0 (c 1.0 CH_3_COOC_2_H_5_); ^1^H NMR (400 MHz, CDCl_3_) δ = 7.94 (d, *J* = 8.5 Hz, 2H), 7.39 (d, *J* = 8.5 Hz, 2H), 7.36–7.08 (m, 10H), 5.76 (d, *J* = 10.0 Hz, 1H), 4.64 (dt, *J* = 9.9, 7.0 Hz, 1H), 3.42 (q, *J* = 6.5 Hz, 1H), 2.28 (d, *J* = 7.0 Hz, 2H), 1.57 (br s, 1H), 1.12 (d, *J* = 6.6 Hz, 3H); ^13^C NMR (100 MHz, CDCl_3_) δ = 23.95, 49.39, 58.22, 70.06, 83.44, 126.12, 126.37, 126.43, 126.78, 128.26, 128.35, 128.75, 129.77, 136.33, 137.77, 145.45, 162.82; IR (KBr) ν 3318, 3061, 3028, 2965, 2924, 1651, 1599, 1491, 1452, 1402, 1368, 1333, 1277, 1244, 1170, 1128, 1092, 1074, 1015, 964, 841, 760, 731, 700, 677, 550, 534 cm^−1^; HRMS *m/z* [M + H]^+^ calcd. for [C_24_H_24_ClN_2_O]^+^ 391.1572, found 391.1572.

(S)-N-(((4S,5R)-2-(2-chlorophenyl)-5-phenyl-4,5-dihydrooxazol-4-yl)methyl)-1-phenylethanamine (**4g**)

Yellow oil, 84.8% yield, [α]_D_^20^ = −259.2 (c 1.0 CH_3_COOC_2_H_5_); ^1^H NMR (400 MHz, CDCl_3_) δ = 7.80 (dd, *J* = 7.7, 1.6 Hz, 1H), 7.46 (dd, *J* = 8.0, 0.8 Hz, 1H), 7.39–7.06 (m, 12H), 5.79 (d, *J* = 10.1 Hz, 1H), 4.69 (dt, *J* = 10.0, 6.9 Hz, 1H), 3.43 (q, *J* = 6.6 Hz, 1H), 2.39–2.20 (m, 2H), 1.54 (br s, 1H), 1.13 (d, *J* = 6.6 Hz, 3H); ^13^C NMR (100 MHz, CDCl_3_) δ = 24.05, 49.39, 58.18, 70.12, 83.55, 126.43, 126.44, 126.63, 126.72, 127.45, 128.17, 128.30, 128.32, 130.77, 131.45, 131.74, 133.53, 136.05, 145.37, 162.47; IR (KBr) ν 3323, 3061, 3028, 2961, 1651, 1593, 1493, 1477, 1452, 1435, 1368, 1331, 1279, 1240, 1132, 1096, 1036, 961, 914, 762, 735, 700, 654 cm^−1^; HRMS *m/z* [M + H]^+^ calcd. for [C_24_H_24_ClN_2_O]^+^ 391.1572, found 391.1575.

(S)-1-phenyl-N-(((4S,5R)-5-phenyl-2-(pyridin-4-yl)-4,5-dihydrooxazol-4-yl)methyl)ethanamine (**4h**)

Yellow oil, 82% yield, [α]_D_^20^ = −302.7 (c 1.0 CH_3_COOC_2_H_5_); ^1^H NMR (400 MHz, CDCl_3_) δ = 8.74 (d, *J* = 5.9 Hz, 2H), 7.85 (d, *J* = 5.9 Hz, 2H), 7.40–7.09 (m, 10H), 5.82 (d, *J* = 10.1 Hz, 1H), 4.70 (dt, *J* = 10.0, 7.1 Hz, 1H), 3.44 (q, *J* = 6.5 Hz, 1H), 2.39–2.20 (m, 2H), 1.97 (br s, 1H), 1.15 (d, *J* = 6.6 Hz, 3H); ^13^C NMR (100 MHz, CDCl_3_) δ = 23.78, 49.16, 58.22, 70.09, 83.63, 122.05, 126.30, 126.41, 126.88, 128.37, 128.42, 134.88, 135.83, 145.06, 150.38, 162.03; IR (KBr) ν 3061, 3030, 2963, 2924, 1655, 1599, 1558, 1493, 1452, 1410, 1368, 1339, 1277, 1210, 1128, 1094, 1080, 1063, 991, 961, 837, 760, 745, 700, 681 cm^−1^; HRMS *m/z* [M + H]^+^ calcd. for [C_23_H_24_N_3_O]^+^ 358.1914, found 358.1914.

(S)-1-phenyl-N-(((4S,5R)-5-phenyl-2-(pyridin-2-yl)-4,5-dihydrooxazol-4-yl)methyl)ethanamine (**4i**)

Yellow oil, 87% yield, [α]_D_^20^ = −261.0 (c 1.0 CH_3_COOC_2_H_5_); ^1^H NMR (400 MHz, CDCl_3_) δ = 8.74 (d, *J* = 4.2 Hz, 1H), 8.06 (d, *J* = 7.9 Hz, 1H), 7.79 (td, *J* = 7.8, 1.6 Hz, 1H), 7.48–7.35 (m, 1H), 7.35–7.01 (m, 10H), 5.85 (d, *J* = 10.1 Hz, 1H), 4.89–4.67 (m, 1H), 3.52 (q, *J* = 6.5 Hz, 1H), 3.25 (s, 1H), 2.43–2.12 (m, 2H), 1.19 (d, *J* = 6.6 Hz, 3H); ^13^C NMR (100 MHz, CDCl_3_) δ = 23.31, 49.11, 58.32, 69.63, 83.76, 124.09, 125.81, 126.36, 126.59, 127.01, 128.30, 128.34, 128.36, 135.87, 136.79, 144.34, 146.37, 150.03, 162.96; IR (KBr) ν 3314, 3059, 3028, 2961, 2924, 1653, 1647, 1582, 1570, 1493, 1468, 1452, 1439, 1368, 1341, 1246, 1117, 1098, 1043, 993, 962, 799, 745, 700, 621 cm^−1^; HRMS *m/z* [M + H]^+^ calcd. for [C_23_H_24_N_3_O]^+^ 358.1914, found 358.1913.

2-((4S,5R)-5-phenyl-4-((((S)-1-phenylethyl)amino)methyl)-4,5-dihydrooxazol-2-yl)phenol (**4j**)

White solid, 87% yield, mp:76~79 °C, [α]_D_^20^ = −256.6 (c 1.0 CH_3_COOC_2_H_5_); ^1^H NMR (400 MHz, CDCl_3_) δ = 11.96 (s, 1H), 7.74 (dd, *J* = 7.8, 1.5 Hz, 1H), 7.43–7.14 (m, 9H), 7.11 (d, *J* = 7.0 Hz, 2H), 7.03 (d, *J* = 8.3 Hz, 1H), 6.87 (t, *J* = 7.3 Hz, 1H), 5.75 (d, *J* = 9.9 Hz, 1H), 4.68 (dt, *J* = 9.8, 6.9 Hz, 1H), 3.39 (q, *J* = 6.6 Hz, 1H), 2.34–2.22 (m, 2H), 1.11 (d, *J* = 6.6 Hz, 3H); ^13^C NMR (100 MHz, CDCl_3_) δ = 23.89, 49.25, 58.14, 68.82, 82.51, 110.43, 116.85, 118.84, 126.36, 126.49, 126.93, 128.31, 128.46, 128.53, 133.70, 135.59, 145.18, 160.00, 165.44; IR (KBr) ν 3061, 3028, 2961, 2924, 2849, 1643, 1616, 1491, 1454, 1368, 1352, 1312, 1258, 1231, 1155, 1130, 1070, 1032, 961, 756, 700, 571, 540 cm^−1^; HRMS *m/z* [M + H]^+^ calcd. for [C_24_H_25_N_2_O_2_]^+^ 373.1911, found 373.1909.

2-((4S,5R)-5-phenyl-4-((((S)-1-phenylethyl)amino)methyl)-4,5-dihydrooxazol-2-yl)aniline (**4k**)

Yellow oil, 82% yield, [α]_D_^20^ = −285.7 (c 1.0 CH_3_COOC_2_H_5_); ^1^H NMR (400 MHz, CDCl_3_) δ = 7.79 (d, *J* = 7.1 Hz, 1H), 7.35–7.11 (m, 11H), 6.66 (dd, *J* = 13.6, 7.6 Hz, 2H), 6.04 (s, 2H), 5.65 (d, *J* = 9.9 Hz, 1H), 4.68 (dt, *J* = 9.7, 7.1 Hz, 1H), 3.41 (q, *J* = 6.5 Hz, 1H), 2.36–2.13 (m, 2H), 1.44 (br s, 1H), 1.11 (d, *J* = 6.6 Hz, 3H); ^13^C NMR (100 MHz, CDCl_3_) δ = 23.91, 49.76, 58.21, 70.06, 81.32, 108.70, 115.75, 116.14, 126.46, 126.49, 126.85, 128.35, 128.41, 129.91, 132.37, 136.66, 145.54, 148.80, 164.07; IR (KBr) ν 3466, 3296, 3061, 3028, 2961, 2924, 1636, 1609, 1595, 1560, 1490, 1454, 1368, 1348, 1319, 1263, 1161, 1078, 1053, 970, 914, 750, 700, 569, 538 cm^−1^; HRMS *m/z* [M + H]^+^ calcd. for [C_24_H_26_N_3_O]^+^ 372.2070, found 372.2070.

2-((4S,5R)-5-phenyl-4-((((S)-1-phenylethyl)amino)methyl)-4,5-dihydrooxazol-2-yl)naphthalen-1-ol (**4l**)

Dark green solid, 88% yield, mp: 79~81 °C, [α]_D_^20^ = +166.9 (c 1.0 CH_3_COOC_2_H_5_); ^1^H NMR (400 MHz, CDCl_3_) δ = 13.11 (br s, 1H), 8.42 (d, *J* = 8.2 Hz, 1H), 7.77 (d, *J* = 7.8 Hz, 1H), 7.72 (d, *J* = 8.7 Hz, 1H), 7.57 (td, *J* = 7.0, 1.4 Hz, 1H), 7.51 (td, *J* = 6.9, 1.3 Hz, 1H), 7.40–7.05 (m, 11H), 5.81 (d, *J* = 9.8 Hz, 1H), 4.74 (dt, *J* = 9.8, 6.9 Hz, 1H), 3.43 (q, *J* = 6.6 Hz, 1H), 2.37–2.28 (m, 2H), 1.13 (d, *J* = 6.6 Hz, 3H); ^13^C NMR (100 MHz, CDCl_3_) δ = 23.87, 49.32, 58.13, 68.52, 82.68, 103.37, 118.20, 123.47, 123.70, 124.75, 125.54, 126.37, 126.51, 126.90, 127.54, 128.44, 128.50, 128.52, 135.62, 136.28, 145.18, 158.85, 166.38; IR (KBr) ν 3061, 3030, 2959, 2924, 2851, 1628, 1599, 1578, 1466, 1450, 1408, 1391, 1304, 1260, 1140, 1078, 964, 810, 756, 700 cm^−1^; HRMS *m/z* [M + H]^+^ calcd. for [C_28_H_27_N_2_O_2_]^+^ 423.2067, found 423.2064.

(S)-1-phenyl-N-(((4S,5R)-5-phenyl-2-(thiophen-2-yl)-4,5-dihydrooxazol-4-yl)methyl)ethanamine (**4m**)

Yellow oil, 90% yield, [α]_D_^20^ = −256.6 (c 1.0 CH_3_COOC_2_H_5_); ^1^H NMR (400 MHz, CDCl_3_) δ = 7.67 (d, *J* = 2.2 Hz, 1H), 7.48 (d, *J* = 4.5 Hz, 1H), 7.41–7.03 (m, 11H), 5.77 (d, *J* = 9.8 Hz, 1H), 4.62 (q, *J* = 7.1 Hz, 1H), 3.51–3.27 (m, 1H), 2.34–2.24 (m, 2H), 1.53 (br s, 1H), 1.11 (d, *J* = 6.4 Hz, 3H); ^13^C NMR (100 MHz, CDCl_3_) δ = 24.01, 49.28, 58.21, 70.05, 83.67, 126.43, 126.74, 127.71, 128.26, 128.33, 130.19, 130.21, 130.66, 136.18, 145.38, 159.54; IR (KBr) ν 3323, 3082, 3061, 3026, 2963, 2924, 1649, 1522, 1493, 1452, 1433, 1369, 1329, 1277, 1217, 1128, 1082, 1059, 1018, 959, 851, 760, 716, 700, 650 cm^−1^; HRMS *m/z* [M + H]^+^ calcd. for [C_22_H_23_N_2_OS]^+^ 363.1526, found 363.1526.

(S)-N-(((4S,5R)-2-(furan-2-yl)-5-phenyl-4,5-dihydrooxazol-4-yl)methyl)-1-phenylethanamine (**4n**)

White solid, 84% yield, mp: 45~48 °C; [α]_D_^20^ = −279.1 (c 1.0 CH_3_COOC_2_H_5_); ^1^H NMR (400 MHz, CDCl_3_) δ = 7.57 (s, 1H), 7.42–7.09 (m, 10H), 7.02 (d, *J* = 3.1 Hz, 1H), 6.51 (s, 1H), 5.74 (d, *J* = 9.9 Hz, 1H), 4.69–4.69 (m, 1H), 3.45 (q, *J* = 6.4 Hz, 1H), 2.29 (d, *J* = 6.9 Hz, 2H), 2.00 (br s, 1H), 1.13 (d, *J* = 6.5 Hz, 3H); ^13^C NMR (100 MHz, CDCl_3_) δ = 23.70, 49.24, 58.20, 69.69, 83.35, 111.63, 114.90, 126.38, 126.47, 126.80, 128.30, 128.34, 135.96, 142.71, 145.12, 145.56, 156.15; IR (KBr) ν 3318, 3061, 3028, 2963, 2924, 2855, 1674, 1580, 1560, 1493, 1481, 1452, 1402, 1331, 1229, 1169, 1092, 1011, 962, 885, 754, 700, 631, 596, 552 cm^−1^; HRMS *m/z* [M + H]^+^ calcd. for [C_22_H_23_N_2_O_2_]^+^ 347.1754, found 347.1754.

(S)-N-(((4S,5R)-2-methyl-5-phenyl-4,5-dihydrooxazol-4-yl)methyl)-1-phenylethanamine (**4o**)

Yellow solid, 85% yield, mp: 44~47 °C; [α]_D_^20^ = −252.6 (c 1.0 CH_3_COOC_2_H_5_); ^1^H NMR (400 MHz, CDCl_3_) δ = 7.37–7.04 (m, 10H), 5.57 (d, *J* = 10.0 Hz, 1H), 4.44–4.37 (m, 1H), 3.39 (q, *J* = 6.5 Hz, 1H), 2.24–2.10 (m, 2H), 2.07 (d, *J* = 0.8 Hz, 3H), 1.10 (d, *J* = 6.6 Hz, 3H); ^13^C NMR (100 MHz, CDCl_3_) δ = 14.06, 23.82, 49.41, 58.14, 69.48, 83.12, 126.24, 126.40, 126.69, 128.05, 128.25, 136.43, 145.43, 164.76; IR (KBr) ν 3320, 3061, 3026, 2967, 2928, 1680, 1603, 1493, 1452, 1387, 1369, 1310, 1229, 1130, 1078, 1028, 974, 914, 760, 700, 623, 592, 538 cm^−1^; HRMS *m/z* [M + H]^+^ calcd. for [C_19_H_23_N_2_O]^+^ 295.1805, found 295.1801.

(S)-N-(((4S,5R)-2-benzyl-5-phenyl-4,5-dihydrooxazol-4-yl)methyl)-1-phenylethanamine (**4p**)

White solid, 83% yield, mp: 103~106 °C, [α]_D_^20^ = −248.0 (c 1.0 CH_3_COOC_2_H_5_); ^1^H NMR (400 MHz, CDCl_3_) δ = 7.44–7.15 (m, 11H), 7.08–7.02 (m, 4H), 5.56 (d, *J* = 10.0 Hz, 1H), 4.47–4.40 (m, 1H), 3.69 (q, *J* = 14.6 Hz, 2H), 3.37 (q, *J* = 6.5 Hz, 1H), 2.21–2.05 (m, 2H), 1.52 (br s, 1H), 1.10 (d, *J* = 6.6 Hz, 3H); ^13^C NMR (100 MHz, CDCl_3_) δ = 23.99, 35.08, 49.41, 58.17, 69.41, 83.25, 126.20, 126.40, 126.72, 127.10, 128.00, 128.19, 128.30, 128.65, 129.06, 135.07, 136.32, 145.33, 166.20; IR (KBr) ν 3302, 3057, 3028, 2985, 2932, 2857, 1663, 1603, 1558, 1495, 1449, 1267, 1244, 1219, 1167, 1142, 1126, 1076, 1055, 1028, 993, 972, 920, 822, 784, 736, 723, 700, 575, 538 cm^−1^; HRMS *m/z* [M + H]^+^ calcd. for [C_25_H_27_N_2_O]^+^ 371.2118, found 371.2118.

## 4. Conclusions

In conclusion, we have developed a new isomerization of amides **3** that leads to 2-oxzaolines **4** in a completely highly regio- and stereoselective isomerization manner. Further application of these 2-oxzaolines in catalytic asymmetric reaction is currently underway in our laboratory.

## Figures and Tables

**Table 1:** Isomerization of amide **3a** ^1^.

**Entry**	**Additive (Equiv.)**	**Solvent**	**Time (h)**	**Yield (%) ^2^**
1	-	ClCH_2_CH_2_Cl	6	n.r.
2	-	Toluene	6	n.r.
3	DABCO (1)	ClCH_2_CH_2_Cl	6	n.r.
4	t-BuOK (1)	THF	6	n.r.
5	NaH (1)	THF	6	n.r.
6	HClO_4_ (1)	ClCH_2_CH_2_Cl	0.5	87
7	CF_3_SO_3_H (1)	ClCH_2_CH_2_Cl	0.5	65
8	CH_3_COOH (1)	CH_2_Cl_2_	12	n.r.
9	CF_3_COOH (1)	CH_2_Cl_2_	12	37
10	CF_3_COOH (1)	ClCH_2_CH_2_Cl	0.5	91
11	CF_3_COOH (1)	ClCH_2_CH_2_Cl	2	86
12	CF_3_COOH (0.75)	ClCH_2_CH_2_Cl	0.5	71
13	TMSOTf (1)	ClCH_2_CH_2_Cl	4	72
14	Cu(OTf)_2_ (1)	ClCH_2_CH_2_Cl	4	93
15	Cu(OTf)_2_ (0.5)	ClCH_2_CH_2_Cl	4	90
16	BF_3_·Et_2_O (1)	ClCH_2_CH_2_Cl	6	87

^1^ The reactions were conducted with amide **3a** (1 mmol) in solvent (10 mL) in the presence of additive at reflux. ^2^ Isolated yields.

**Table 2:** Isomerizations of various amides in the presence of Cu(OTf)_2_^1^.

**Entry**	**R**	**Product**	**Yield (%) ^2^**
1	**3a**: C_6_H_5_-	**4a**	96
2	**3b**: 4-MeOC_6_H_4_-	**4b**	94
3	**3c**: 2-MeOC_6_H_4_-	**4c**	93
4	**3d**: 4-O_2_NC_6_H_4_-	**4d**	94
5	**3e**: 2-O_2_NC_6_H_4_-	**4e**	91
6	**3f**: 4-ClC_6_H_4_-	**4f**	79
7	**3g**: 2-ClC_6_H_4_-	**4g**	85
8	**3h**: 4-pyridyl	**4h**	82
9	**3i**: 2-pyridyl	**4i**	87
10 ^3^	**3j**: 2-HOC_6_H_4_-	**4j**	87
11 ^3^	**3k**: 2-H_2_NC_6_H_4_-	**4k**	82
12 ^3^	**3l**: 1-hydroxynaphthalen-2-yl	**4l**	88
13	**3m**: thiophen-2-yl	**4m**	90
14	**3n**: furan-2-yl	**4n**	84
15	**3o**: Me-	**4o**	85
16	**3p**: C_6_H_5_CH_2_-	**4p**	83

^1^ Reactions were conducted with amide **3** (1 mmol) at reflux for 4 h in ClCH_2_CH_2_Cl (10 mL) in the presence of Cu(OTf)_2_ (0.5 mmol). ^2^ Isolated yields. ^3^ Reactions were conducted in the presence of CF_3_COOH (1.5 mmol) at reflux for 30 min.

## Data Availability

The data presented in this study are available in this article.

## Supplementary Materials

The supplementary materials in the text are available online. CCDC 2059257 contain the supplementary crystallographic data for this paper. These data can be obtained free of charge via www.ccdc.cam.ac.uk/data_request/cif (accessed on 6 February 2021), or by emailing data_request@ccdc.cam.ac.uk, or by contacting The Cambridge Crystallographic Data Centre, 12 Union Road, Cambridge CB2 1EZ, UK; fax: +44-1223-336033.

## Sample Availability

Not available.

## References

[1] Wong G.S.K., Wu W. (2004). 2-oxazolines. Chem. Heterocycl. Compd..

[2] Wipf P., Miller C.P. (1992). Total synthesis of westiellamide. J. Am. Chem. Soc..

[3] Xiao S.-J., Guo D.-L., Zhang M.-S., Chen F., Ding L.-S., Zhou Y. (2016). Three new oxazoline alkaloids from Gymnotheca chinensis. J. Asian Nat. Prod. Res..

[4] Campiani G., De Angelis M., Armaroli S., Fattorusso C., Catalanotti B., Ramunno A., Nacci V., Novellino E., Grewer C., Ionescu D. (2001). A rational approach to the design of selective substrates and potent nontransportable inhibitors of the excitatory amino acid transporter EAAC1 (EAAT3). New glutamate and aspartate analogues as potential neuroprotective agents. J. Med. Chem..

[5] Avalos-Alanis F.G., Hernandez-Fernandez E., Carranza-Rosales P., Lopez-Cortina S., Hernandez-Fernandez J., Ordonez M., Guzman-Delgado N.E., Morales-Vargas A., Velazquez-Moreno V.M., Santiago-Mauricio M.G. (2017). Synthesis, antimycobacterial and cytotoxic activity of α,β-unsaturated amides and 2,4-disubstituted oxazoline derivatives. Bioorg. Med. Chem. Lett..

[6] Li Q., Woods K.W., Claiborne A., Gwaltney S.L., Barr K.J., Liu G., Gehrke L., Credo R.B., Hui Y.H., Lee J. (2002). Synthesis and Biological Evaluation of 2-Indolyloxazolines as a New Class of Tubulin Polymerization Inhibitors. Discovery of A-289099 as an Orally Active Antitumor Agent. Bioorg. Med. Chem. Lett..

[7] Ye X.-Y., Liang Z.-Q., Jin C., Lang Q.-W., Chen G.-Q., Zhang X. (2021). Design of oxa-spirocyclic PHOX ligands for the asymmetric synthesis of lorcaserin via iridium-catalyzed asymmetric hydrogenation. Chem. Commun..

[8] Xi C.-C., Zhao X.-J., Tian J.-M., Chen Z.-M., Zhang K., Zhang F.-M., Tu Y.-Q., Dong J.-W. (2020). Atroposelective Synthesis of Axially Chiral 3-Arylindoles by Copper-Catalyzed Asymmetric Cross-Coupling of Indoles with Quinones and Naphthoquinones. Org. Lett..

[9] Park J.-U., Ahn H.-I., Cho H.-J., Xuan Z., Kim J.H. (2020). Asymmetric Synthesis of N-Fused 1,3-Oxazolidines via Pd-Catalyzed Decarboxylative (3+2) Cycloaddition. Adv. Synth. Catal..

[10] Qiu Z., Sun R., Yang K., Teng D. (2019). Spiro indan-based phosphine-oxazolines as highly efficient P,N ligands for enantioselective Pd-catalyzed allylic alkylation of indoles and allylic etherification. Molecules.

[11] Itoh K., Sibi M.P. (2018). Dibenzofuran-4,6-bis(oxazoline) (DBFOX). A novel trans-chelating bis(oxazoline) ligand for asymmetric reactions. Org. Biomol. Chem..

[12] Frump J.A. (1971). Oxazolines. Their preparation, reactions, and applications. Chem. Rev..

[13] Goud D.R., Pathak U. (2012). A mild and efficient synthesis of 2-oxazolines via transamidation-cyclodehydrosulfurization of thioamides with 2-aminoethanol. Synthesis.

[14] Brandstatter M., Roth F., Luedtke N.W. (2015). Synthesis of 2-Oxazolines by in Situ Desilylation and Cyclodehydration of β-Hydroxyamides. J. Org. Chem..

[15] Huang H., Yang W., Chen Z., Lai Z., Sun J. (2019). A mild catalytic synthesis of 2-oxazolines via oxetane ring-opening: Rapid access to a diverse family of natural products. Chem. Sci..

[16] Petersson M.J., Jenkins I.D., Loughlin W.A. (2009). The use of phosphonium anhydrides for the synthesis of 2-oxazolines, 2-thiazolines and 2-dihydrooxazine under mild conditions. Org. Biomol. Chem..

[17] Sharma R., Vadivel S.K., Duclos R.I., Makriyannis A. (2009). Open vessel mode microwave-assisted synthesis of 2-oxazolines from carboxylic acids. Tetrahedron Lett..

[18] Rajaram S., Sigman M.S. (2002). Modular Synthesis of Amine-Functionalized Oxazolines. Org. Lett..

[19] Zhou X., Xu X., Li Y., Zhang Z., Zheng Z.-b. (2016). Synthesis of chiral 4,5-dihydrothiazol-2-amines and 4,5-dihydrooxazol-2-amines through an unexpected ring opening reaction of azetidines. Tetrahedron Lett..

[20] Zhang Z., Bai X., Liu R., Zi G. (2009). Synthesis of new chiral cis-3-aminoazetidines and their use in catalytic asymmetric reactions. Inorg. Chim. Acta.

[21] Higgins R.H., Faircloth W.J., Baughman R.G., Eaton Q.L. (1994). Ring Opening of Azetidinols by Phenols: Regiochemistry and Stereochemistry. J. Org. Chem..

[22] Purkarthofer T., Gruber K., Gruber-Khadjawi M., Waich K., Skranc W., Mink D., Griengl H. (2006). A biocatalytic Henry reaction—The hydroxynitrile lyase from Hevea brasiliensis also catalyzes nitroaldol reactions. Angew. Chem. Int. Ed..

[23] Nishimura M., Minakata S., Takahashi T., Oderaotoshi Y., Komatsu M. (2002). Asymmetric N1 Unit Transfer to Olefins with a Chiral Nitridomanganese Complex: Novel Stereoselective Pathways to Aziridines or Oxazolines. J. Org. Chem..

