# Supplementary material for: Synthesis of 2-Oxazolines from Ring Opening Isomerization of 3-Amido-2-Phenyl Azetidines
Source: Molecules. 2021 Feb 6;26(4):857. doi: 10.3390/molecules26040857 (PMC7914936; doi:10.3390/molecules26040857)
Supplement: Supplementary file 1 [file molecules-26-00857-s001.pdf]

Supporting Information

Synthesis of 2-oxazolines from ring opening isomerization of 3-amido-2-phenyl azetidines

Xin Zhou, Baiyi Mao and Zhanbin Zhang*

College of Chemistry, Beijing Normal University, Beijing 100875, China

Content:

1. General Procedures for the Synthesis of **3** and **4** …………………………. S2
2. Analytic data for compounds **3** …………………………………….... S2-10
3. ^{1}H NMR and ^{13}C NMR spectra of compounds **3**………………………S11-42
4. ^{1}H NMR and ^{13}C NMR spectra of compounds **4**………………………S43-74

* Corresponding author. Tel.: +86-10-58806051; fax: +0-000-000-0000; e-mail: zhangzb@bnu.edu.cn

General Procedure for the Synthesis of amide 3

Acid **2** (2.2mmol), CDI (2.2mmol) was stirred in THF (10mL) for 4h at room temperature. To this solution 3-amino-2-phenyl-azetidine **1** (2 mmol) was added. After being stirred overnight, the mixture was washed with water (10 mL), saturated $NaHCO_3$ (10 mL), and dried over Na_2SO_4. The solvent was removed under reduced pressure. The residue was purified by gradient column chromatography on silica gel with PE/EA (10:1-5:1) as eluent to give amide **3**.

General Procedures for the isomerization of amide 3

a. A mixture of amide **3**(1mmol), $Cu(OTf)_2$ (0.5mmol) in 1,2-dichloroethane (10mL) was refluxed for 4h. The mixture was washed with water (10 mL), saturated $NaHCO_3$ (10 mL), and dried over Na_2SO_4. The solvent was removed under reduced pressure. The residue was purified by gradient column chromatography on silica gel with PE/EA (5:1-2:1) as eluent to give oxazoline **4**.
b. A mixture of amide **3**(1mmol), CF_3COOH (1.5mmol) in 1,2-dichloroethane (10mL) was refluxed for 30min. The mixture was washed with water (10 mL), saturated $NaHCO_3$ (10 mL), and dried over Na_2SO_4. The solvent was removed under reduced pressure. The residue was purified by gradient column chromatography on silica gel with PE/EA (5:1-2:1) as eluent to give oxazoline **4**.

N-((2S,3S)-2-phenyl-1-((S)-1-phenylethyl)azetidin-3-yl)benzamide (3a)

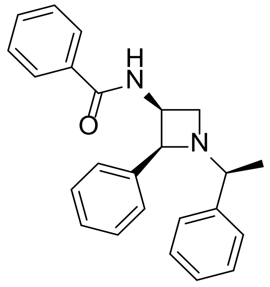

White solid, 94 % yield, mp: 156~158℃, $[\alpha]_D^{20}$ = +160.5 (c 1.0 $CH_3COOC_2H_5$); ^{1}H NMR (400 MHz, $CDCl_3$) δ 7.39 – 7.00 (m, 15H), 6.42 (d, J = 6.8 Hz, 1H), 4.75 (qd, J = 7.6, 2.1 Hz, 1H), 4.57 (d, J = 7.4 Hz, 1H), 3.59 – 3.47 (m, 2H), 3.43 (d, J = 7.4 Hz, 1H), 1.32 (d, J = 6.6 Hz, 3H); ^{13}C NMR (100 MHz, $CDCl_3$) δ 19.00, 45.05, 56.68, 66.31, 69.99, 126.67, 127.20, 127.24, 127.35, 127.87, 127.95, 128.41, 131.29, 134.50, 137.59, 141.87, 167.28; IR (KBr) ν 3377, 3061, 3030, 2986, 2959, 2845, 2820, 2785, 1651, 1603, 1580, 1528, 1450, 1452, 1352, 1273, 1227, 1167, 1074, 1028, 978, 843, 758, 716, 694, 538 cm^{-1}; HRMS m/z $[M+H]^+$ calced for $C_{24}H_{25}N_2O^+$ 357.1961, found 357.1961.

4-methoxy-N-((2S,3S)-2-phenyl-1-((S)-1-phenylethyl)azetidin-3-yl)benzamide (3b)

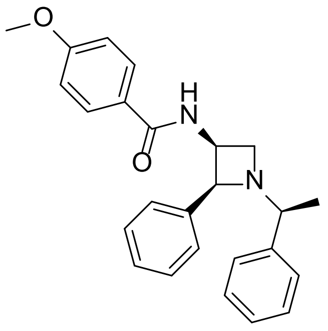

Yellow solid, 86% yield, mp: 147~150℃, $[\alpha]_D^{20}$ = +222.9 (c 1.0 $CH_3COOC_2H_5$); ^{1}H NMR (400 MHz, $CDCl_3$) δ 7.32 (d, *J* = 8.7 Hz, 2H), 7.20 – 6.95 (m, 10H), 6.73 (d, *J* = 8.8 Hz, 2H), 6.34 (d, *J* = 6.6 Hz, 1H), 4.74 (qd, *J* = 7.6, 2.1 Hz, 1H), 4.56 (d, *J* = 7.5 Hz, 1H), 3.70 (s, 3H), 3.54 - 3.42 (m, 3H), 1.32 (d, *J* = 6.6 Hz, 3H); ^{13}C NMR (100 MHz, $CDCl_3$) δ 19.03, 44.95, 55.29, 56.78, 66.32, 70.00, 113.57, 126.79, 127.14, 127.19, 127.37, 127.84, 127.90, 128.08, 128.45, 137.70, 141.95, 161.98, 166.74; IR (KBr) ν 3375, 3030, 2959, 2887, 2843, 2787, 1643, 1609, 1576, 1535, 1504, 1425, 1366, 1352, 1182, 1165, 1113, 1072, 843, 764, 756, 700, 694, 611, 536 cm^{-1}; HRMS m/z $[M+H]^+$ calced for $C_{25}H_{27}N_2O_2^+$ 387.2067, found 387.2067.

2-methoxy-N-((2S,3S)-2-phenyl-1-((S)-1-phenylethyl)azetidin-3-yl)benzamide (3c)

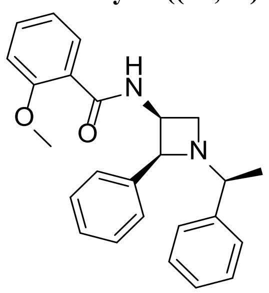

Yellow oil, 95 % yield, $[\alpha]_D^{20}$ = +212.1 (c 1.0 $CH_3COOC_2H_5$); ^{1}H NMR (400 MHz, $CDCl_3$) δ 8.08 (d, *J* = 7.4 Hz, 1H), 8.01 (d, *J* = 4.4 Hz, 1H), 7.35 – 6.83 (m, 12H), 6.72 (d, *J* = 8.2 Hz, 1H), 4.76 (s, 1H), 4.56 (d, *J* = 7.4 Hz, 1H), 3.51 (s, 3H), 3.43 (s, 3H), 1.32 (d, *J* = 6.1 Hz, 3H); ^{13}C NMR (100 MHz, $CDCl_3$) δ 18.99, 45.79, 55.45, 57.58, 66.45, 69.13, 111.08, 120.92, 121.04, 126.94, 127.13, 127.70, 127.76, 127.81, 128.12, 132.01, 132.70, 138.03, 142.14, 157.60, 165.21; IR (KBr) ν 3379, 3061, 3028, 2967, 2928, 2845, 1649, 1599, 1508, 1481, 1452, 1364, 1292, 1238, 1161, 1103, 1072, 1022, 754, 700, 669, 536 cm^{-1}; HRMS m/z $[M+H]^+$ calced for $C_{25}H_{27}N_2O_2^+$ 387.2067, found 387.2067.

4-nitro-N-((2S,3S)-2-phenyl-1-((S)-1-phenylethyl)azetidin-3-yl)benzamide (3d)

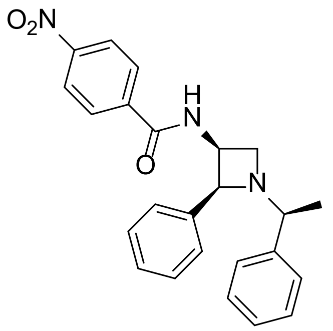

White solid, 90 % yield, mp: 144~147℃; $[\alpha]_D^{20}$ = +109.2 (c 1.0 $CH_3COOC_2H_5$); ^{1}H NMR (400 MHz, $CDCl_3$) δ 8.13 (d, *J* = 8.8 Hz, 2H), 7.45 (d, *J* = 8.3 Hz, 2H), 7.25 – 6.97 (m, 10H), 6.42 (s, 1H), 4.77 (q, *J* = 6.8 Hz, 1H), 4.64 (d, *J* = 7.2 Hz, 1H), 3.59 – 3.55 (m, 2H), 3.47– 3.45 (m, 1H), 1.38 (d, *J* = 6.6 Hz, 2H); ^{13}C NMR (100 MHz, $CDCl_3$) δ 18.71, 45.47, 56.21, 66.15, 69.96, 123.66, 127.22, 127.38, 127.39, 127.78, 127.92, 128.03, 137.19, 140.02, 141.43, 149.37, 165.26; IR (KBr) ν 3420, 3300, 3063, 3030, 2964, 2859, 2781, 1645, 1601, 1526, 1493, 1452, 1346, 1290, 1277, 1167, 1109, 1074, 1028, 1015, 866, 837, 758, 721, 698 cm^{-1}; HRMS m/z $[M+H]^+$ calced for $C_{24}H_{24}N_3O_3^+$ 402.1812, found 402.1811.

2-nitro-N-((2S,3S)-2-phenyl-1-((S)-1-phenylethyl)azetidin-3-yl)benzamide (3e)

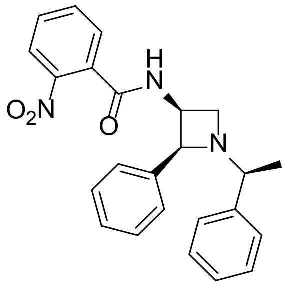

Yellow solid, 84 % yield, mp: 149~152℃, $[\alpha]_D^{20}$ = +36.5 (c 1.0 $CH_3COOC_2H_5$); ^{1}H NMR (400 MHz, $CDCl_3$) δ 7.91 (d, *J* = 7.8 Hz, 1H), 7.49 – 7.34 (m, 2H), 7.29 – 6.95 (m, 10H), 6.48 (d, *J* = 6.5 Hz, 1H), 6.10 (s, 1H), 4.85 (q, *J* = 7.1 Hz, 1H), 4.61 (d, *J* = 7.0 Hz, 1H), 3.59 – 3.55 (m, 2H), 3.46 (d, *J* = 7.6 Hz, 1H), 1.35 (d, *J* = 6.5 Hz, 3H); ^{13}C NMR (100 MHz, $CDCl_3$) δ 18.63, 45.35, 55.85, 65.90, 70.10, 124.34, 126.99, 127.26, 127.37, 127.84, 127.87, 128.02, 130.31, 132.59, 133.43, 137.63, 141.76, 146.32, 165.96; IR (KBr) ν 3418, 2949, 2924, 2857, 1730, 1489, 1452, 1443, 1383, 1341, 1269, 1215, 1138, 1094, 1062, 1013, 934, 878, 812, 760, 698, 675 cm^{-1}; HRMS m/z $[M+H]^+$ calced for $C_{24}H_{24}N_3O_3^+$ 302.1812, found 402.1812.

4-chloro-N-((2S,3S)-2-phenyl-1-((S)-1-phenylethyl)azetidin-3-yl)benzamide (3f)

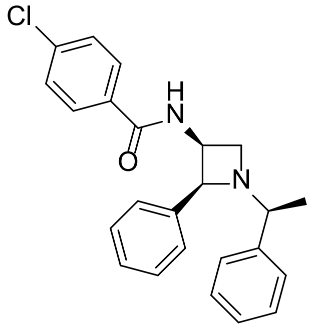

White solid, 77 % yield, mp: 145~148℃, $[\alpha]_D^{20}$ = +216.6 (c 1.0 $CH_3COOC_2H_5$); ^{1}H NMR (400 MHz, $CDCl_3$) δ 7.37 – 6.92 (m, 14H), 6.39 (d, *J* = 6.1 Hz, 1H), 4.74 – 4.71 (m, 1H), 4.57 (d, *J* = 7.1 Hz, 1H), 3.63 – 3.31 (m, 3H), 1.33 (d, *J* = 6.2 Hz, 3H); ^{13}C NMR (100 MHz, $CDCl_3$) δ 18.94, 45.15, 56.44, 66.26, 69.98, 127.23, 127.27, 127.30, 127.88, 127.94, 128.07, 128.11, 128.63, 132.88, 137.42, 137.54, 141.77, 166.23; IR (KBr) ν 3292, 3061, 3028, 2968, 2926, 2841, 1638, 1595, 1541, 1533, 1485, 1450, 1364, 1275, 1164, 1092, 1028, 1015, 845, 756, 698, 536 cm^{-1}; HRMS m/z $[M+H]^+$ calced for $C_{24}H_{24}ClN_2O^+$ 391.1572, found 391.1572.

2-chloro-N-((2S,3S)-2-phenyl-1-((S)-1-phenylethyl)azetidin-3-yl)benzamide (3g)

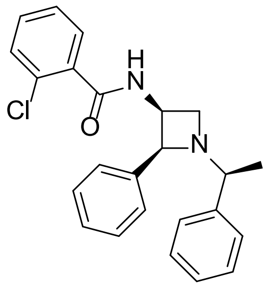

White solid, 82 % yield, mp: 95~98℃, $[\alpha]_D^{20}$ = +117.4 (c 1.0 $CH_3COOC_2H_5$); ^{1}H NMR (400 MHz, $CDCl_3$) δ 7.27 – 6.94 (m, 14H), 6.46 (d, *J* = 6.9 Hz, 1H), 4.81 (qd, *J* = 7.2, 1.7 Hz, 1H), 4.60 (d, *J* = 7.4 Hz, 1H), 3.58 – 3.51 (m, 3H), 1.34 (d, *J* = 6.6 Hz, 3H); ^{13}C NMR (100 MHz, $CDCl_3$) δ 18.71, 45.60, 56.36, 65.94, 69.78, 126.77, 127.08, 127.22, 127.44, 127.86, 127.90, 128.05, 129.93, 130.04, 130.68, 131.12, 134.66, 137.56, 141.87, 166.18; IR (KBr) ν 3273, 3057, 3026, 2974, 2924, 2868, 2847, 1639, 1531, 1493, 1450, 1431, 1366, 1283, 1167, 1051, 1028, 968, 856, 768, 754, 721, 700, 671, 540 cm^{-1}; HRMS m/z $[M+H]^+$ calced for $C_{24}H_{24}ClN_2O^+$ 391.1572, found 391.1572.

N-((2S,3S)-2-phenyl-1-((S)-1-phenylethyl)azetidin-3-yl)isonicotinamide (3h)

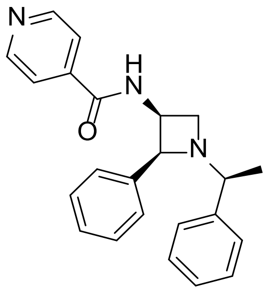

White solid, 88 % yield, mp: 90~93℃, $[\alpha]_D^{20}$ = +158.8 (c 1.0 $CH_3COOC_2H_5$); ^{1}H NMR (400 MHz, $CDCl_3$) δ 8.53 (d, J = 5.3 Hz, 2H), 7.25 – 6.95 (m, 12H), 6.90 (d, J = 7.8 Hz, 1H), 4.76 (q, J = 6.9 Hz, 1H), 4.57 (d, J = 7.2 Hz, 1H), 3.56 – 3.48 (m, 2H), 3.41 (d, J = 8.1 Hz, 1H), 1.32 (d, J = 6.5 Hz, 3H); ^{13}C NMR (100 MHz, $CDCl_3$) δ 18.80, 45.35, 55.77, 66.05, 70.09, 120.68, 127.23, 127.26, 127.33, 127.86, 128.00, 137.45, 141.64, 141.68, 150.25, 165.46; IR (KBr) ν 3294, 3061, 3028, 2967, 2926, 2846, 1651, 1537, 1493, 1452, 1408, 1368, 1356, 1290, 1231, 1167, 1072, 1028, 841, 754, 698, 669, 538 cm^{-1}; HRMS m/z $[M+H]^+$ calced for $C_{23}H_{24}N_3O^+$ 358.1914, found 358.1914.

N-((2S,3S)-2-phenyl-1-((S)-1-phenylethyl)azetidin-3-yl)picolinamide (3i)

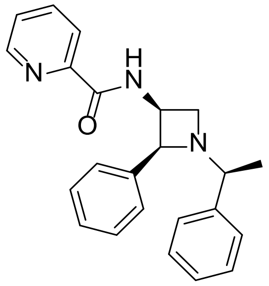

Yellow oil, 92 % yield, $[\alpha]_D^{20}$ = +239.1 (c 1.0 $CH_3COOC_2H_5$); ^{1}H NMR (400 MHz, $CDCl_3$) δ 8.45 – 8.39 (m, 2H), 7.93 (d, J = 7.8 Hz, 1H), 7.65 (td, J = 7.7, 1.6 Hz, 1H), 7.34 – 7.11 (m, 5H), 7.10 – 6.90 (m, 6H), 4.79 (td, J = 9.0, 2.4 Hz, 1H), 4.51 (d, J = 7.2 Hz, 1H), 3.77 – 3.30 (m, 3H), 1.33 (d, J = 6.6 Hz, 3H); ^{13}C NMR (100 MHz, $CDCl_3$) δ 19.15, 45.20, 56.55, 66.41, 70.60, 121.82, 125.93, 126.86, 127.08, 127.53, 127.75, 128.09, 137.01, 137.53, 141.96, 147.98, 149.53, 164.14; IR (KBr) ν 3381, 3059, 3028, 2968, 2928, 2843, 1682, 1589, 1570, 1516, 1464, 1452, 1433, 1371, 1275, 1240, 1167, 1072, 1028, 997, 856, 818, 750, 698, 621, 538 cm^{-1}; HRMS m/z $[M+H]^+$ calced for $C_{23}H_{24}N_3O^+$ 358.1914, found 358.1914

2-hydroxy-N-((2S,3S)-2-phenyl-1-((S)-1-phenylethyl)azetidin-3-yl)benzamide (3j)

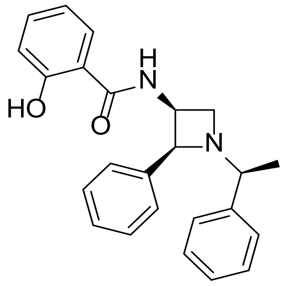

Yellow oil, 85 % yield, $[\alpha]_D^{20}$ = +189.5 (c 1.0 $CH_3COOC_2H_5$); ^{1}H NMR (400 MHz, $CDCl_3$) δ 12.04 (s, 1H), 7.29(td, J = 7.5, 1.3 Hz, 1H), 7.22 – 6.96 (m, 10H), 6.87 (d, J = 8.3 Hz, 1H), 6.81 (d, J = 6.2 Hz, 1H), 6.69 (t, J = 7.5 Hz, 1H), 6.53 (s, 1H), 4.79 – 4.45 (m, 2H), 3.77 – 3.25 (m, 3H), 1.36 (d, J = 6.6 Hz, 3H); ^{13}C NMR (100 MHz, $CDCl_3$) δ 18.96, 44.87, 56.59, 66.42, 69.61, 114.17, 118.40, 118.63, 125.16, 127.32, 127.56, 127.89, 128.10, 128.14, 134.10, 137.06, 141.65, 161.36, 169.74; IR (KBr) ν 3422, 3335, 3061, 3028, 2968, 2928, 2845, 1643, 1595, 1531, 1493, 1452, 1366, 1302, 1254, 1232, 1169, 1072, 1028, 980, 866, 810, 752, 698, 663, 534 cm^{-1}; HRMS m/z $[M+H]^+$ calced for $C_{24}H_{25}N_2O_2^+$ 373.1911, found 373.1911.

2-amino-N-((2S,3S)-2-phenyl-1-((S)-1-phenylethyl)azetidin-3-yl)benzamide (3k)

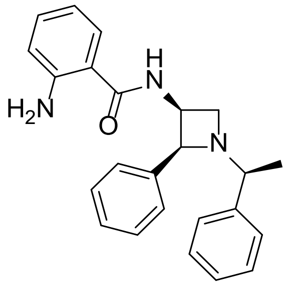

White solid, 79 % yield, mp:61~65℃, $[\alpha]_D^{20}$ = +98.6 (c 1.0 $CH_3COOC_2H_5$); ^{1}H NMR (400 MHz, $CDCl_3$) δ 7.20 – 7.17 (m, 4H), 7.13 (t, J = 7.4 Hz, 2H), 7.06 (t, J = 7.2 Hz, 4H), 7.02 – 6.96 (m, 1H), 6.67 (d, J = 7.7 Hz, 1H), 6.51 (d, J = 8.1 Hz, 1H), 6.44 (t, J = 7.4 Hz, 1H), 6.28 (d, J = 7.2 Hz, 1H), 5.23 (s, 2H), 4.70 (qd, J = 7.5, 1.8 Hz, 1H), 4.55 (d, J = 7.4 Hz, 1H), 3.55 – 3.46 (m, 1H), 3.41 (d, J = 8.0 Hz, 1H), 1.32 (d, J = 6.6 Hz, 3H); ^{13}C NMR (100 MHz, $CDCl_3$) δ 18.97, 44.77, 56.77, 66.27, 69.91, 116.03, 116.49, 117.05, 126.98, 127.21, 127.33, 127.86, 127.97, 128.09, 132.12, 137.67, 141.96, 148.43, 168.97; IR (KBr) ν 3435, 3345, 3061, 3026, 2967, 2926, 2849, 1641, 1585, 1508, 1450, 1364, 1260, 1161, 1072, 1028, 868, 750, 698, 556, 536, 503 cm^{-1}; HRMS m/z $[M+H]^+$ calced for $C_{24}H_{26}N_3O^+$ 372.2070, found 372.2070.

1-hydroxy-N-((2S,3S)-2-phenyl-1-((S)-1-phenylethyl)azetidin-3-yl)-2-naphthamide (3l)

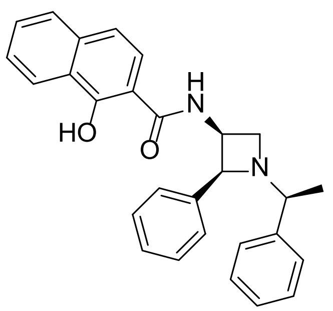

Yellow solid, 87 % yield, mp: 79~81℃, $[\alpha]_D^{20}$ = +166.9 (c 1.0 $CH_3COOC_2H_5$); ^{1}H NMR (400 MHz, $CDCl_3$) δ 13.57 (s, 1H), 8.34 (d, J = 8.2 Hz, 1H), 7.65 (d, J = 8.0 Hz, 1H), 7.58 – 7.37 (m, 2H), 7.28 – 7.12 (m, 6H), 7.12 – 6.93 (m, 5H), 6.76 (d, J = 8.8 Hz, 1H), 6.55 (s, 1H), 4.76 – 4.62 (m, 1H), 4.58 (d, J = 7.3 Hz, 1H), 3.64 – 3.34 (m, 3H), 1.33 (d, J = 6.6 Hz, 3H); ^{13}C NMR (100 MHz, $CDCl_3$) δ 18.99, 44.87, 56.76, 66.49, 69.59, 106.53, 118.13, 120.55, 123.80, 125.52, 125.79, 127.30, 127.34, 127.60, 127.88, 128.11, 128.84, 136.19, 137.05, 141.67, 160.45, 17.40; IR (KBr) ν 3424, 3059, 3028, 2966, 2918, 2847, 1624, 1595, 1528, 1501, 1466, 1414, 1393, 1362, 1275, 1258, 1121, 1169, 1072, 1026, 980, 793, 764, 698, 658, 534, 492 cm^{-1}; HRMS m/z $[M+H]^+$ calced for $C_{28}H_{27}N_2O_2^+$ 423.2067, found 423.2066.

N-((2S,3S)-2-phenyl-1-((S)-1-phenylethyl)azetidin-3-yl)thiophene-2-carboxamide (3m)

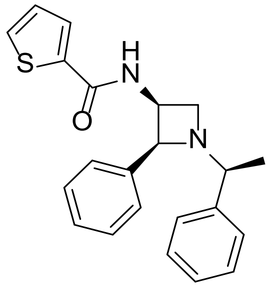

White solid, 90 % yield, mp:119~122℃, $[\alpha]_D^{20}$ = +210.7 (c 1.0 $CH_3COOC_2H_5$);^{1}H NMR (400 MHz, $CDCl_3$) δ 7.32 (d, J = 4.9 Hz, 1H), 7.18 – 6.18 (m, 11H), 6.94 – 6.86 (m, 1H), 6.20 (s, 1H), 4.79 – 4.62 (m, 1H), 4.56 (d, J = 7.4 Hz, 1H), 3.59 – 3.41 (m, 3H), 1.33 (d, J = 6.6 Hz, 3H); ^{13}C NMR (100 MHz, $CDCl_3$) δ 18.98, 44.98, 56.81, 66.34, 66.74, 127.22, 127.32, 127.42, 127.67, 127.83, 128.01, 128.07, 129.72, 137.32, 138.74, 141.79, 161.51; IR (KBr) ν 3374, 3028, 2960, 2922, 2891, 2852, 1634, 1537, 1514, 1454, 1417, 1358, 1275, 1167, 1130, 1072, 1029, 978, 860, 762, 718, 694, 605, 538 cm^{-1}; HRMS m/z $[M+H]^+$ calced for $C_{22}H_{23}N_2OS^+$ 363.1526, found 363.1526.

N-((2S,3S)-2-phenyl-1-((S)-1-phenylethyl)azetidin-3-yl)furan-2-carboxamide (3n)

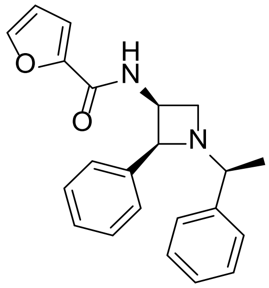

White solid, 87 % yield, mp:105~107℃, $[\alpha]_D^{20}$ = +229.8 (c 1.0 $CH_3COOC_2H_5$); ^{1}H NMR (400 MHz, $CDCl_3$) δ 7.29 – 7.26 (m, 1H), 7.20 – 7.16 (m, 4H), 7.14 – 6.96 (m, 6H), 6.87 (d, *J* = 3.4 Hz, 1H), 6.61 (d, *J* = 6.8 Hz, 1H), 6.34 (dd, *J* = 3.4, 1.8 Hz, 1H), 4.76 – 4.65 (m, 1H), 4.53 (d, *J* = 7.3 Hz, 1H), 3.57 – 3.39 (m, 3H), 1.33 (d, *J* = 6.6 Hz, 3H); ^{13}C NMR (100 MHz, $CDCl_3$) δ 18.90, 44.64, 56.71, 66.24, 69.94, 111.82, 113.77, 127.14, 127.42, 127.79, 127.81, 128.04, 137.24, 141.84, 143.77, 147.76, 158.05; IR (KBr) ν 3410, 3318, 3084, 3061, 3028, 2968, 2928, 2843, 1667, 1593, 1518, 1474, 1452, 1371, 1277, 1225, 1173, 1072, 1028, 1009, 914, 883, 824, 756, 698, 596, 538 cm^{-1}; HRMS m/z $[M+H]^+$ calced for $C_{22}H_{23}N_2O_2^+$ 347.1754, found 347.1752.

N-((2S,3S)-2-phenyl-1-((S)-1-phenylethyl)azetidin-3-yl)acetamide (3o)

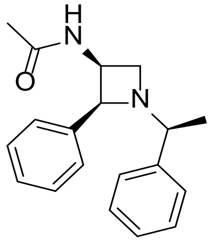

White solid, 90 % yield, mp:118~121℃, $[\alpha]_D^{20}$ = +60.4 (c 1.0 $CH_3COOC_2H_5$); ^{1}H NMR (400 MHz, $CDCl_3$) δ 7.18 (d, *J* = 7.0 Hz, 2H), 7.15 – 6.97 (m, 8H), 5.67 (s, 1H), 4.57 (td, *J* = 8.8, 1.8 Hz, 1H), 4.48 (d, *J* = 7.4 Hz, 1H), 3.51 (q, *J* = 6.6 Hz, 1H), 3.44 (t, *J* = 7.6 Hz, 1H), 3.34 (d, *J* = 8.1 Hz, 1H), 1.67 (s, 3H), 1.33 (d, *J* = 6.6 Hz, 3H); ^{13}C NMR (100 MHz, $CDCl_3$) δ 18.91, 22.96, 45.02, 56.22, 66.18, 70.12, 126.95, 127.16, 127.40, 127.65, 127.80, 127.90, 128.01, 137.55, 141.84, 170.03; IR (KBr) ν 3304, 3086, 3065, 3024, 2959,2920, 2858, 2774, 1653, 1558, 1493, 1449, 1373, 1292, 1281, 1240, 1168, 1125, 1074, 1063, 1020, 974, 758, 696, 590, 542 cm^{-1}; HRMS m/z $[M+H]^+$ calced for $C_{19}H_{23}N_2O^+$ 295.1805, found 295.1804.

2-phenyl-N-((2S,3S)-2-phenyl-1-((S)-1-phenylethyl)azetidin-3-yl)acetamide (3p)

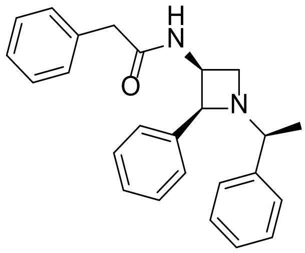

Yellow solid, 82 % yield, mp: 95~98℃, $[\alpha]_D^{20}$= +95.7 (c 1.0 $CH_3COOC_2H_5$); ^{1}H NMR (400 MHz, $CDCl_3$) δ 7.20 – 6.90 (m, 14H), 6.81 (d, *J* = 4.0 Hz, 2H), 5.76 (d, *J* = 7.6 Hz, 1H), 4.60 (q, *J* =7.1 Hz, 1H), 4.40 (d, *J* = 7.5 Hz, 1H), 3.52 – 3.08 (m, 5H), 1.24 (d, *J* = 6.5 Hz, 3H); ^{13}C NMR (100 MHz, $CDCl_3$) δ 19.02, 43.58, 44.61, 56.40, 66.19, 69.82, 126.85, 127.12, 127.17, 127.21, 127.80, 127.82, 128.04, 128.92, 129.40, 134.29, 137.39, 141.80, 170.78; IR (KBr) ν 3283, 3061, 3028, 2968, 2926, 2841, 1645, 1547, 1495, 1452, 1368, 1275, 1074, 1028, 983, 725, 699, 538 cm^{-1}; HRMS m/z $[M+H]^+$ calced for $C_{25}H_{27}N_2O^+$ 371.2118, found 371.2118.

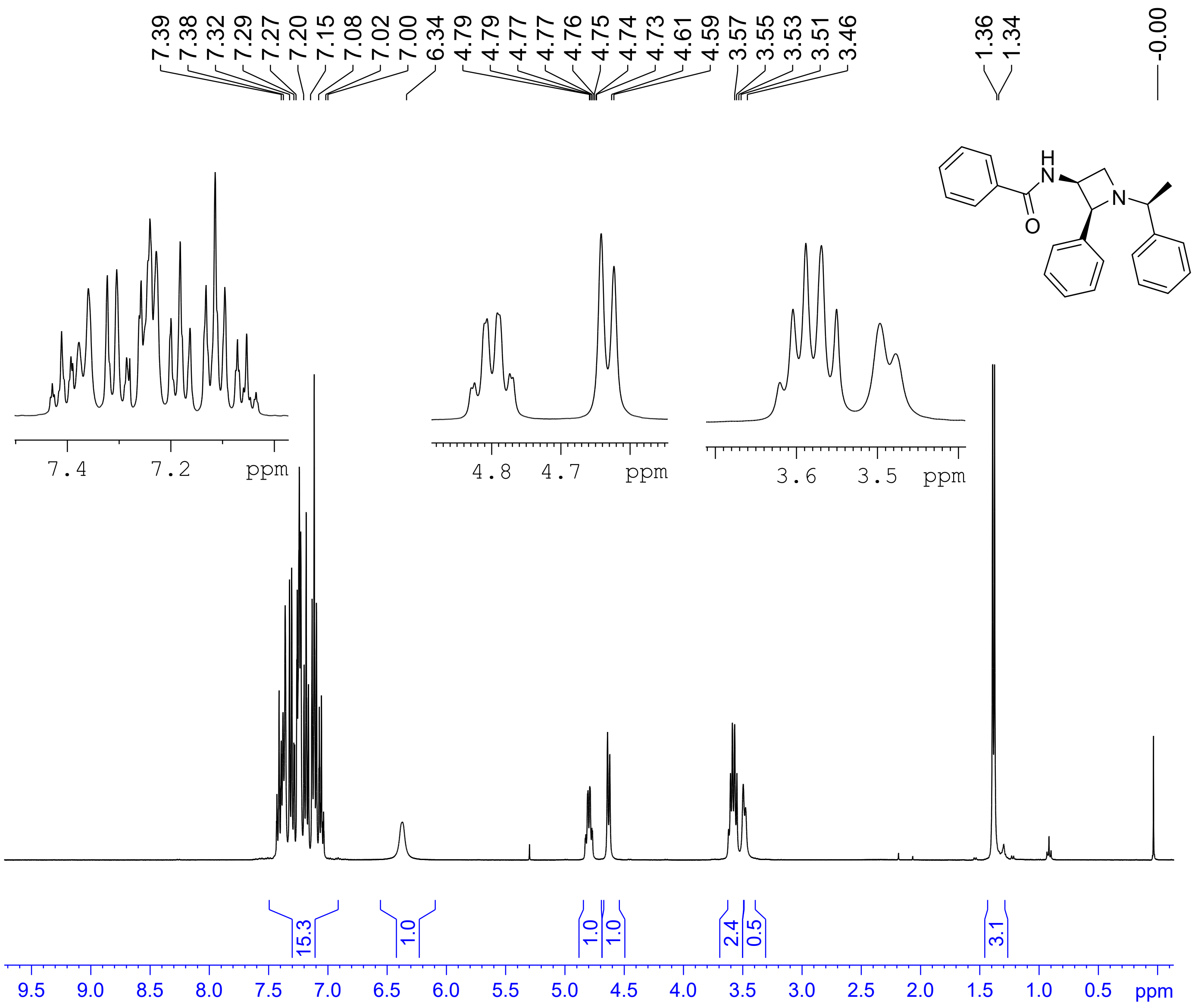

3a ¹H NMR

```
NAME       3a ZX-Ph-CisPhamide
EXPNO                        1
PROCNO                       1
Date_                 20141128
Time                     16.58
INSTRUM                  spect
PROBHD        5 mm PABBO BB-
PULPROG                   zg30
TD                       65536
SOLVENT                  CDCl3
NS                          16
DS                           2
SWH                   8223.685 Hz
FIDRES                0.125483 Hz
AQ                   3.9846387 sec
RG                        80.6
DW                      60.800 usec
DE                        6.50 usec
TE                       296.2 K
D1                  1.00000000 sec
TD0                          1

======== CHANNEL f1 ========
NUC1                        1H
P1                       13.80 usec
PL1                      -1.00 dB
PL1W               13.18669796 W
SFO1               400.1724712 MHz
SI                       32768
SF                 400.1699961 MHz
WDW                         EM
SSB                          0
LB                        0.30 Hz
GB                           0
PC                        1.00
```

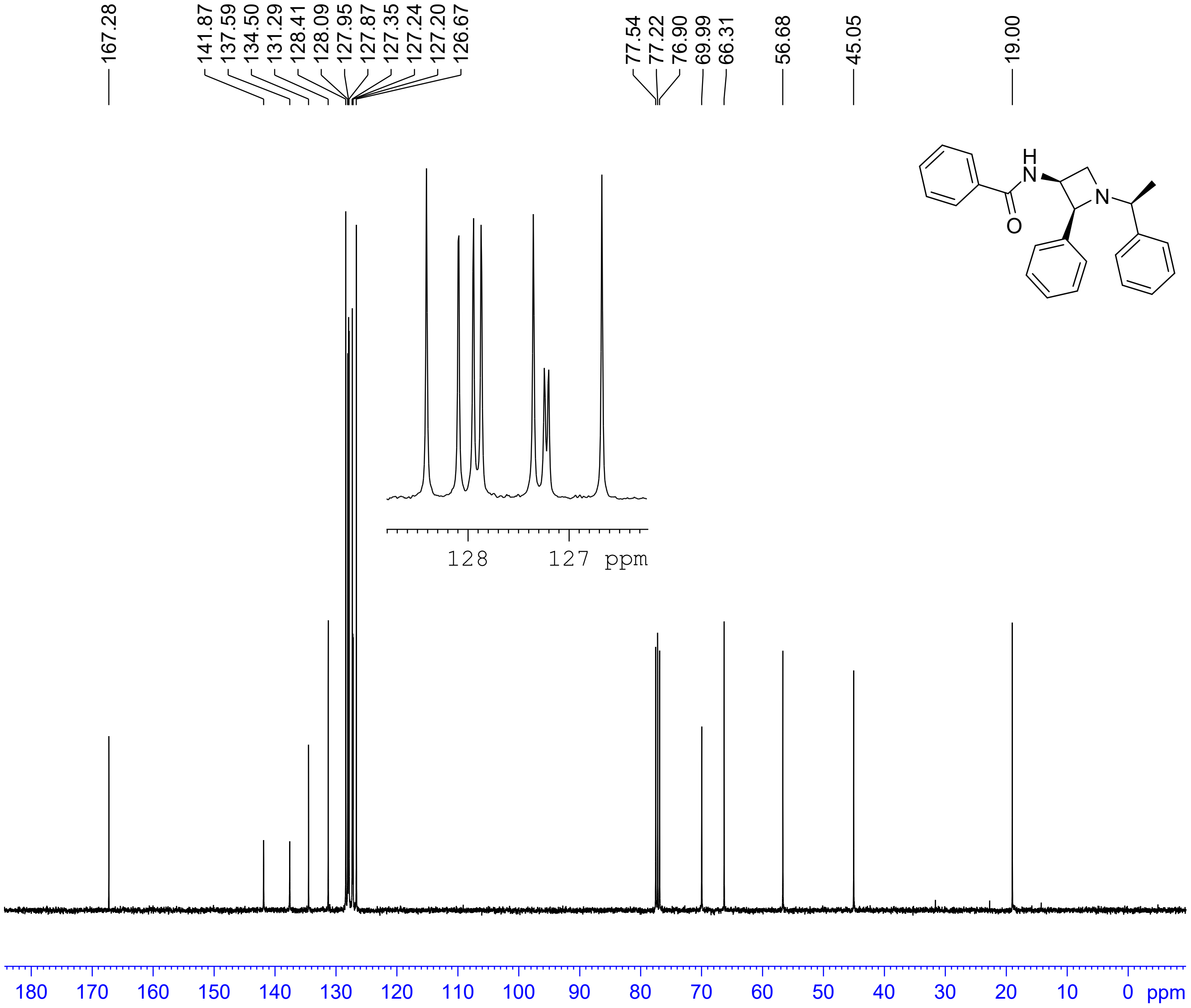


```
NAME        3a ZX-Ph-CisPhamide-C13
EXPNO                     1
PROCNO                    1
Date_              20141202
Time                  17.16
INSTRUM               spect
PROBHD      5 mm PABBO BB-
PULPROG             zgpg30
TD                    65536
SOLVENT               CDCl3
NS                      117
DS                        4
SWH              24038.461 Hz
FIDRES            0.366798 Hz
AQ               1.3631988 sec
RG                      203
DW                   20.800 usec
DE                     6.50 usec
TE                    292.4 K
D1               2.00000000 sec
D11              0.03000000 sec
TD0                       1

======== CHANNEL f1 ========
NUC1                    13C
P1                     8.50 usec
PL1                   -2.00 dB
PL1W            57.32743073 W
SFO1            100.6328888 MHz

======== CHANNEL f2 ========
CPDPRG2             waltz16
NUC2                     1H
PCPD2                 80.00 usec
PL2                   -1.00 dB
PL12                  14.26 dB
PL13                  14.46 dB
PL2W            13.18669796 W
PL12W            0.39276794 W
PL13W            0.37509048 W
SFO2            400.1716007 MHz
SI                    32768
SF              100.6228270 MHz
WDW                      EM
SSB                       0
LB                     1.00 Hz
GB                        0
PC                     1.40
```

3a ^{13}C NMR

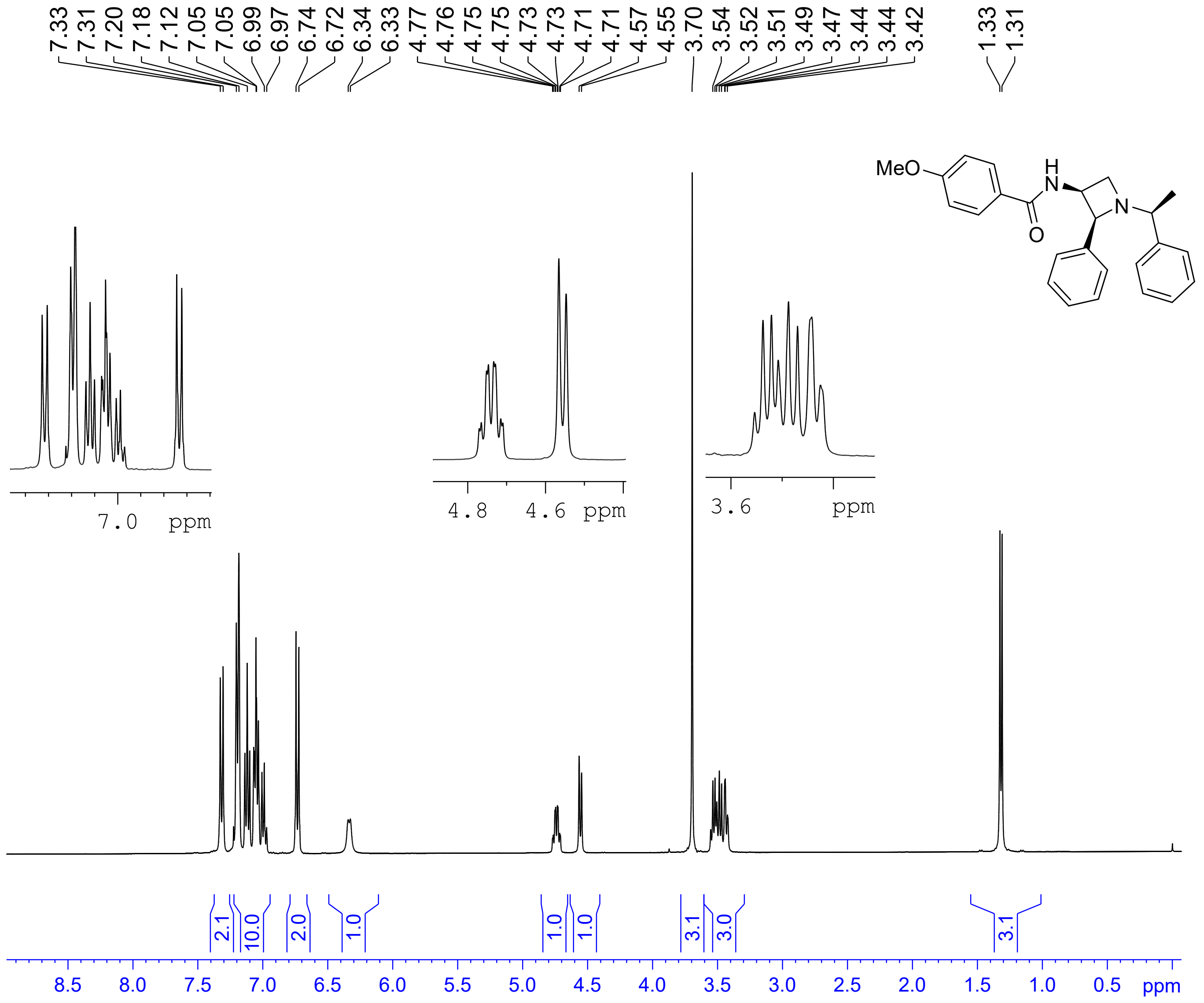

NAME	3b ZX-pOME-CisPhamide
EXPNO	1
PROCNO	1
Date_	20150331
Time	9.53
INSTRUM	spect
PROBHD	5 mm PABBO BB-
PULPROG	zg30
TD	65536
SOLVENT	CDCl3
NS	16
DS	2
SWH	8223.685 Hz
FIDRES	0.125483 Hz
AQ	3.9846387 sec
RG	25.4
DW	60.800 usec
DE	6.50 usec
TE	296.1 K
D1	1.00000000 sec
TD0	1

======== CHANNEL f1 ========

NUC1	1H
P1	13.80 usec
PL1	-1.00 dB
PL1W	13.18669796 W
SFO1	400.1724712 MHz
SI	32768
SF	400.1700180 MHz
WDW	EM
SSB	0
LB	0.30 Hz
GB	0
PC	1.00

3b ^{1}H NMR

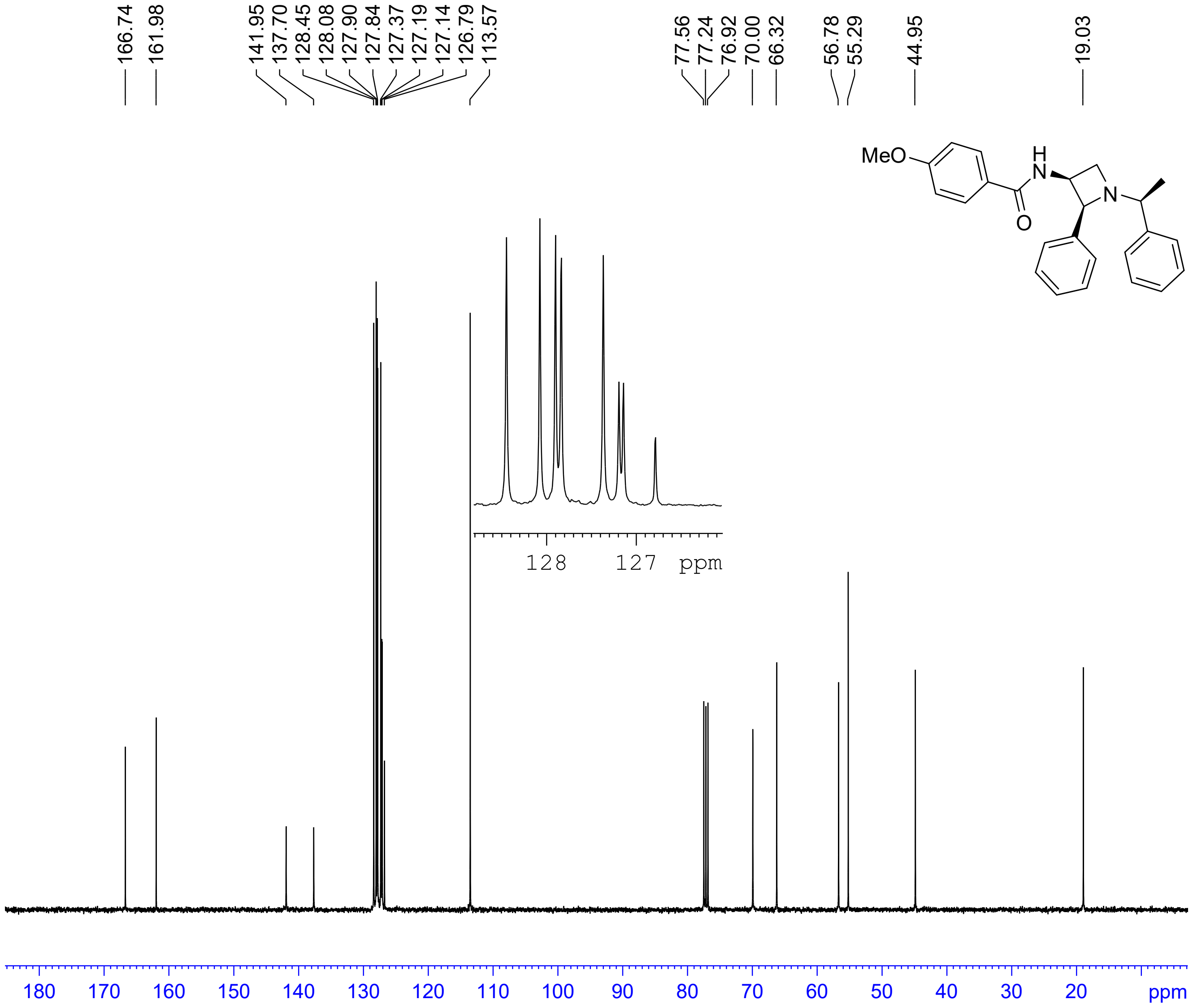

NAME	3b ZX-pOME-CisPhamide-C13	
EXPNO	1	
PROCNO	1	
Date_	20150331	
Time	11.36	
INSTRUM	spect	
PROBHD	5 mm PABBO BB-	
PULPROG	zgpg30	
TD	65536	
SOLVENT	CDCl3	
NS	150	
DS	4	
SWH	24038.461	Hz
FIDRES	0.366798	Hz
AQ	1.3631988	sec
RG	203	
DW	20.800	usec
DE	6.50	usec
TE	296.1	K
D1	2.00000000	sec
D11	0.03000000	sec
TD0	1	
======== CHANNEL f1 ========		
NUC1	13C	
P1	8.50	usec
PL1	-2.00	dB
PL1W	57.32743073	W
SFO1	100.6328888	MHz
======== CHANNEL f2 ========		
CPDPRG2	waltz16	
NUC2	1H	
PCPD2	80.00	usec
PL2	-1.00	dB
PL12	14.26	dB
PL13	14.46	dB
PL2W	13.18669796	W
PL12W	0.39276794	W
PL13W	0.37509048	W
SFO2	400.1716007	MHz
SI	32768	
SF	100.6228270	MHz
WDW	EM	
SSB	0	
LB	1.00	Hz
GB	0	
PC	1.40	

3b ^{13}C NMR

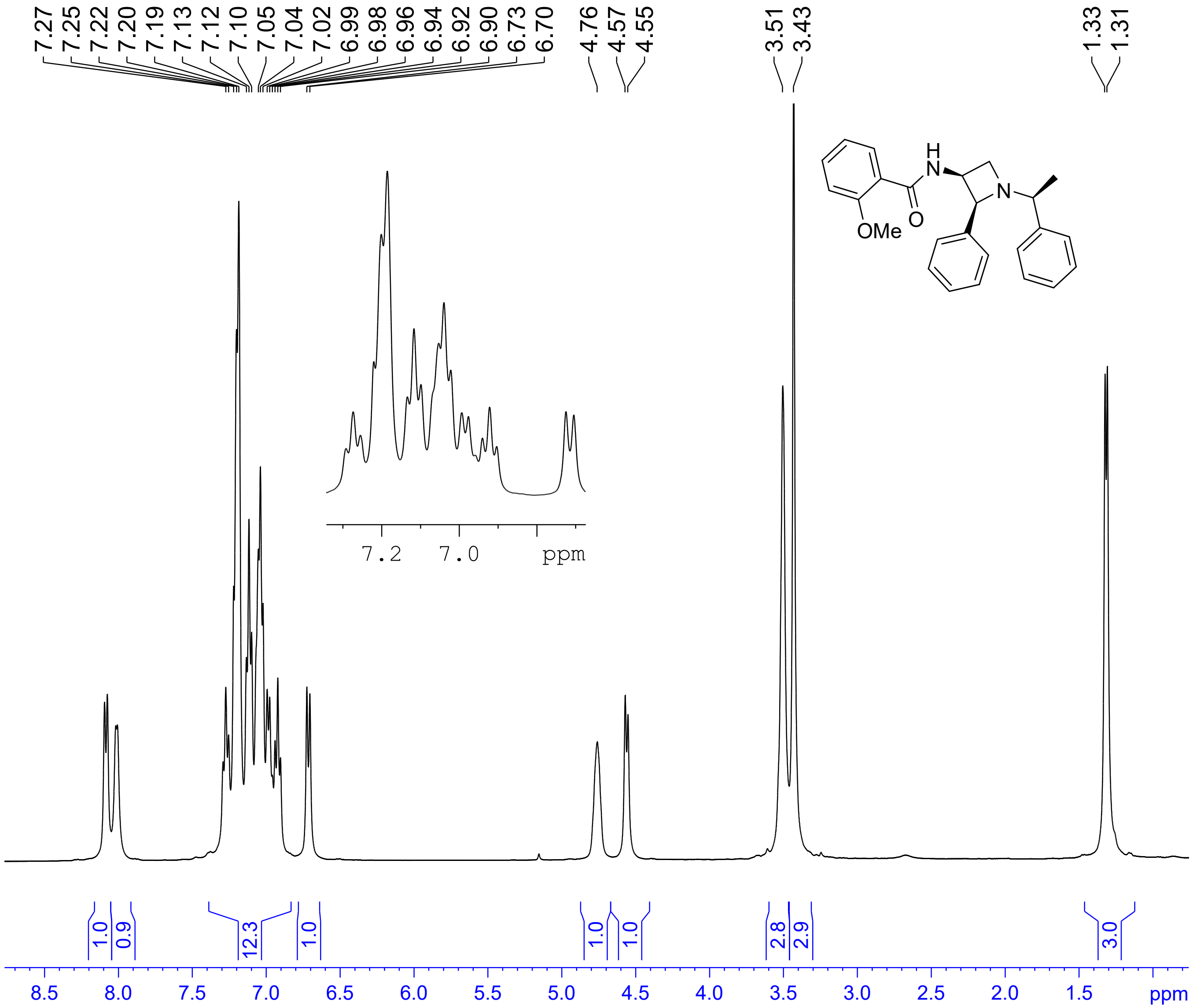

3c ^{1}H NMR

```
NAME         3c ZX-oOMe-CisPhamide
EXPNO                         1
PROCNO                        1
Date_                  20141110
Time                      21.18
INSTRUM                   spect
PROBHD        5 mm PABBO BB-
PULPROG                    zg30
TD                        65536
SOLVENT                   CDCl3
NS                           16
DS                            2
SWH                    8223.685 Hz
FIDRES                 0.125483 Hz
AQ                    3.9846387 sec
RG                           18
DW                       60.800 usec
DE                         6.50 usec
TE                        292.5 K
D1                   1.00000000 sec
TD0                           1

======== CHANNEL f1 ========
NUC1                         1H
P1                        13.80 usec
PL1                       -1.00 dB
PL1W                13.18669796 W
SFO1                400.1724712 MHz
SI                        32768
SF                  400.1700201 MHz
WDW                          EM
SSB                           0
LB                         0.30 Hz
GB                            0
PC                         1.00
```

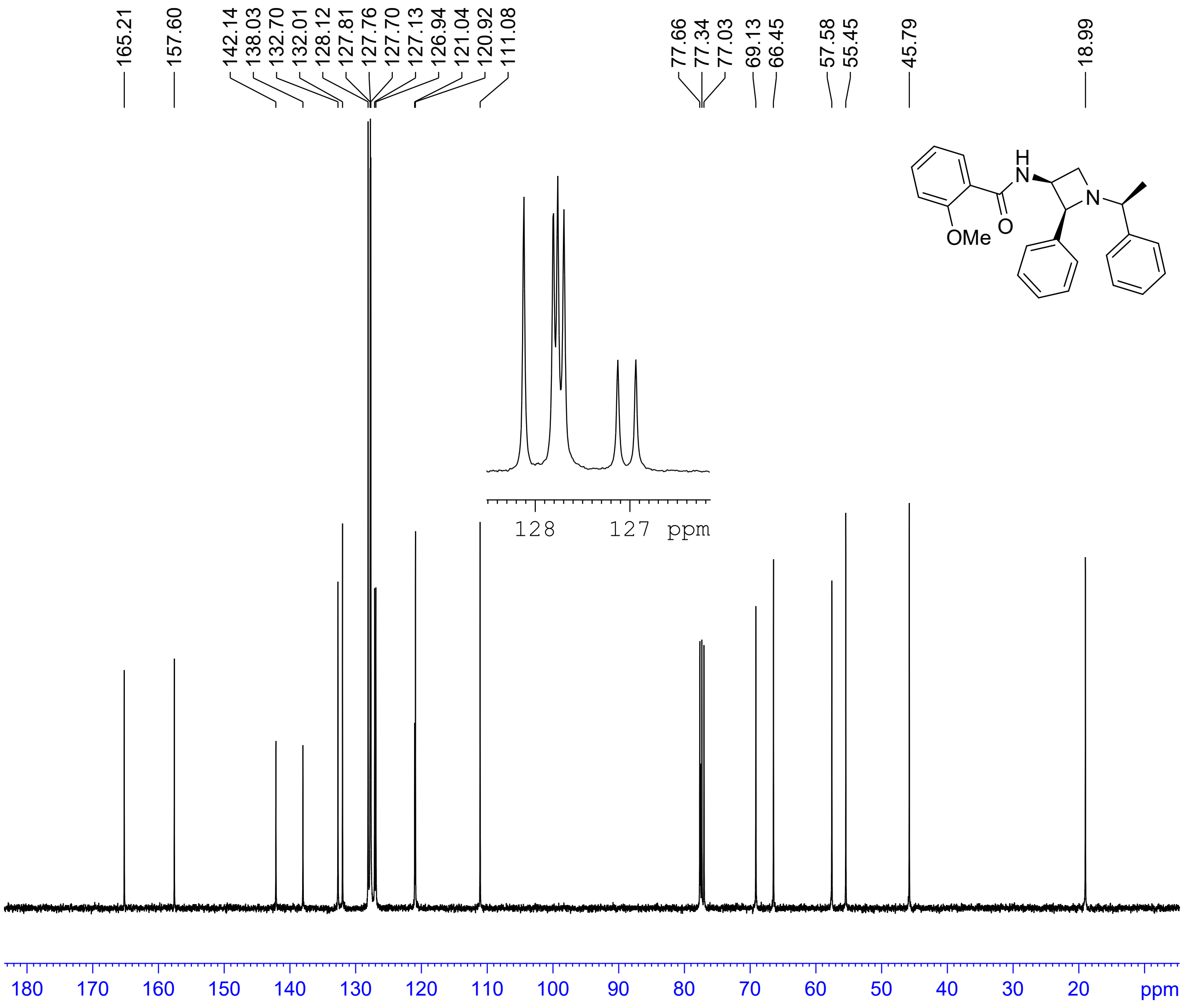


```
NAME        3c ZX-oOMe-CisPhamide-C13
EXPNO                     1
PROCNO                    1
Date_              20141110
Time                  21.28
INSTRUM               spect
PROBHD    5 mm PABBO BB-
PULPROG              zgpg30
TD                    65536
SOLVENT               CDCl3
NS                      128
DS                        4
SWH               24038.461 Hz
FIDRES             0.366798 Hz
AQ                1.3631988 sec
RG                      203
DW                   20.800 usec
DE                     6.50 usec
TE                    293.2 K
D1               2.00000000 sec
D11              0.03000000 sec
TD0                       1

======== CHANNEL f1 ========
NUC1                    13C
P1                     8.50 usec
PL1                   -2.00 dB
PL1W            57.32743073 W
SFO1            100.6328888 MHz

======== CHANNEL f2 ========
CPDPRG2             waltz16
NUC2                     1H
PCPD2                 80.00 usec
PL2                   -1.00 dB
PL12                  14.26 dB
PL13                  14.46 dB
PL2W            13.18669796 W
PL12W            0.39276794 W
PL13W            0.37509048 W
SFO2            400.1716007 MHz
SI                    32768
SF              100.6228270 MHz
WDW                      EM
SSB                       0
LB                     1.00 Hz
GB                        0
PC                     1.40
```

3c ^{13}C NMR

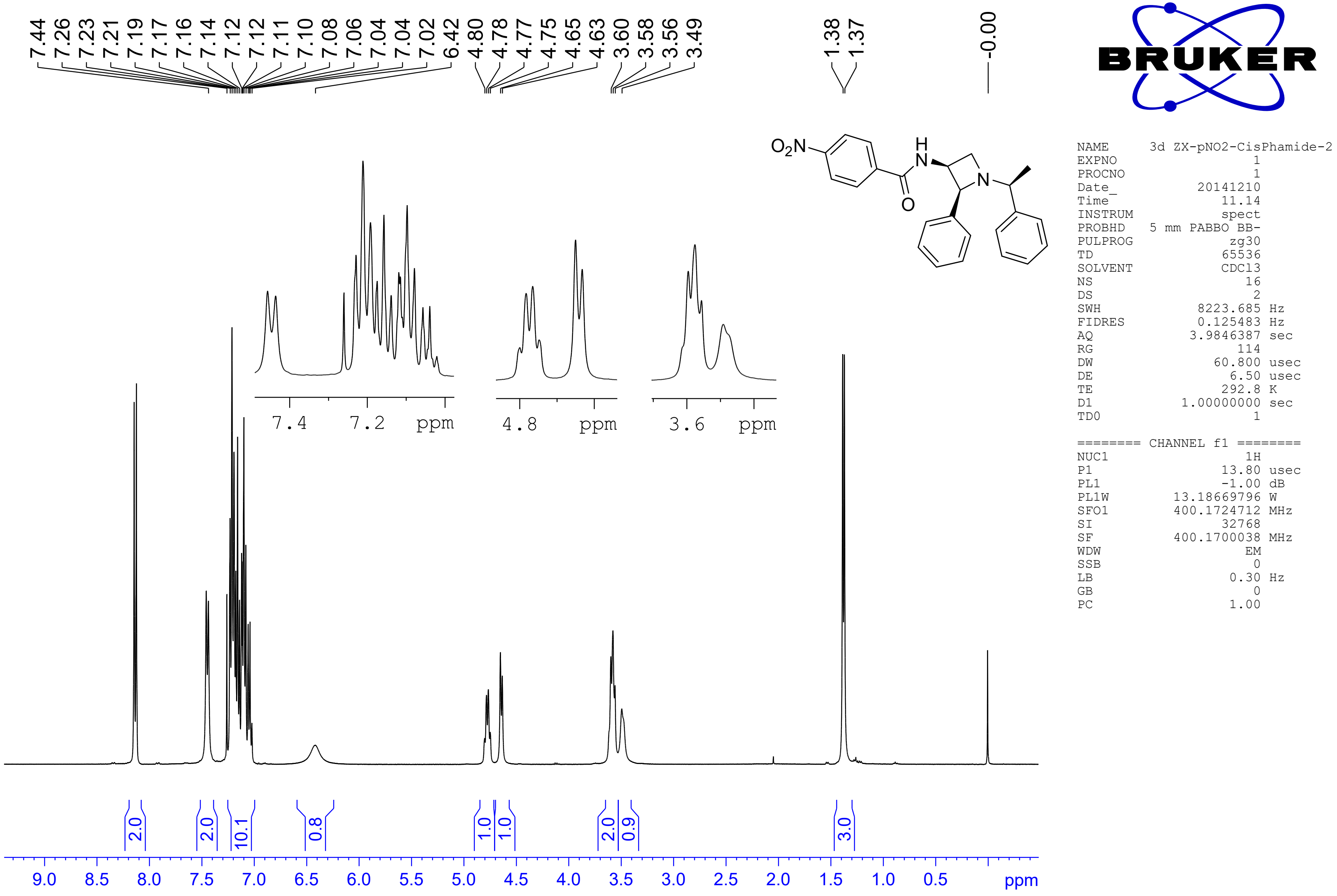
7.44 7.26 7.23 7.21 7.19 7.17 7.16 7.14 7.12 7.12 7.11 7.10 7.08 7.06 7.04 7.04 7.02 6.42 4.80 4.78 4.77 4.75 4.65 4.63 3.60 3.58 3.56 3.49 1.38 1.37 -0.00
BRUKER
NAME 3d ZX-pNO2-CisPhamide-2
EXPNO 1
PROCNO 1
Date_ 20141210
Time 11.14
INSTRUM spect
PROBHD 5 mm PABBO BB-
PULPROG zg30
TD 65536
SOLVENT CDCl3
NS 16
DS 2
SWH 8223.685 Hz
FIDRES 0.125483 Hz
AQ 3.9846387 sec
RG 114
DW 60.800 usec
DE 6.50 usec
TE 292.8 K
D1 1.00000000 sec
TD0 1
======== CHANNEL f1 ========
NUC1 1H
P1 13.80 usec
PL1 -1.00 dB
PL1W 13.18669796 W
SFO1 400.1724712 MHz
SI 32768
SF 400.1700038 MHz
WDW EM
SSB 0
LB 0.30 Hz
GB 0
PC 1.00
2.0 2.0 10.1 0.8 1.0 1.0 2.0 0.9 3.0
9.0 8.5 8.0 7.5 7.0 6.5 6.0 5.5 5.0 4.5 4.0 3.5 3.0 2.5 2.0 1.5 1.0 0.5 ppm

3d ^{1}H NMR

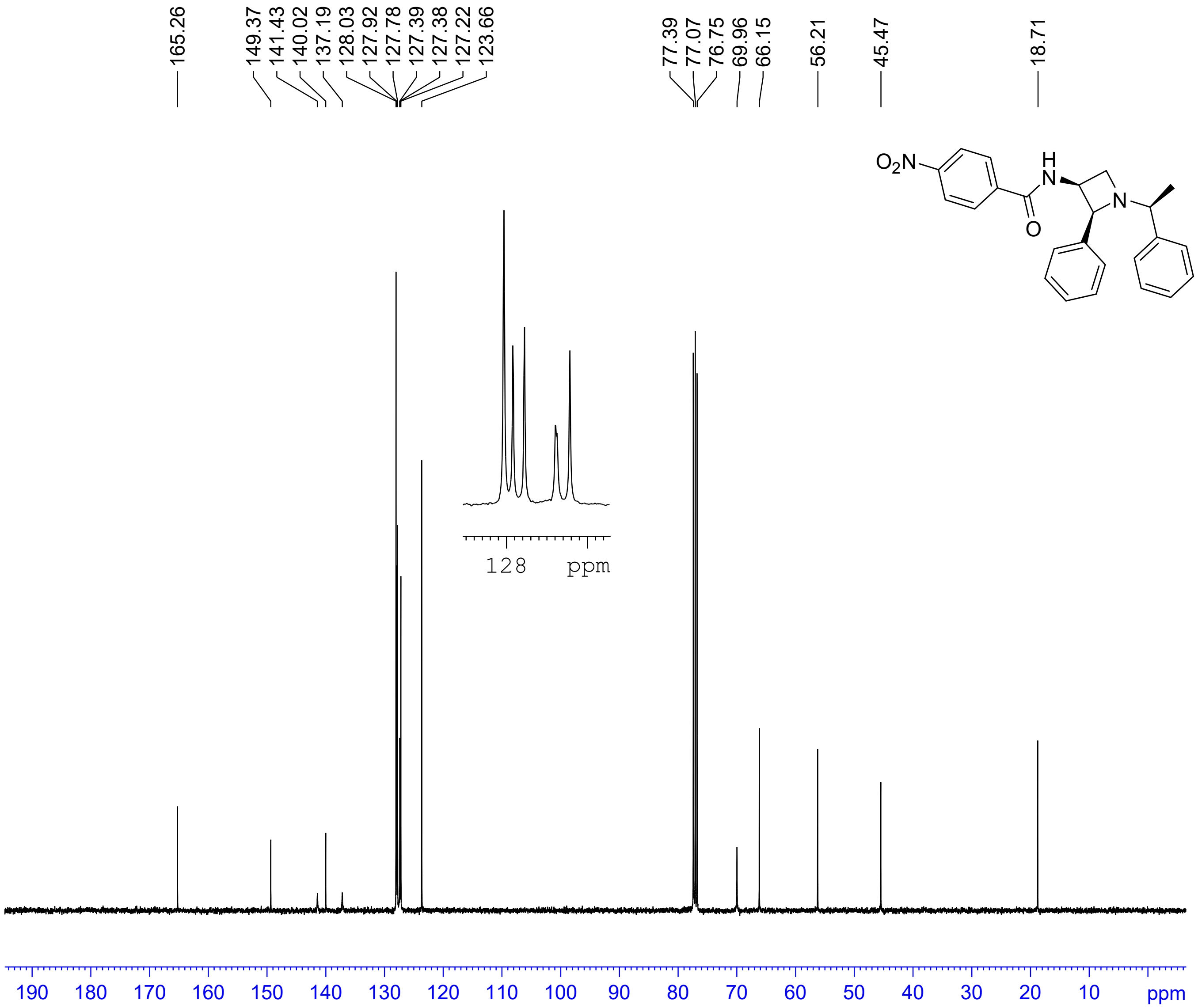


```
NAME       3d ZX-pNO2-CisPhamide-2-C13
EXPNO                     1
PROCNO                    1
Date_              20141210
Time                  18.51
INSTRUM               spect
PROBHD     5 mm PABBO BB-
PULPROG             zgpg30
TD                    65536
SOLVENT               CDCl3
NS                      430
DS                        4
SWH               24038.461 Hz
FIDRES             0.366798 Hz
AQ                1.3631988 sec
RG                      203
DW                   20.800 usec
DE                     6.50 usec
TE                    293.9 K
D1               2.00000000 sec
D11              0.03000000 sec
TD0                       1

======== CHANNEL f1 ========
NUC1                    13C
P1                     8.50 usec
PL1                   -2.00 dB
PL1W            57.32743073 W
SFO1            100.6328888 MHz

======== CHANNEL f2 ========
CPDPRG2             waltz16
NUC2                     1H
PCPD2                 80.00 usec
PL2                   -1.00 dB
PL12                  14.26 dB
PL13                  14.46 dB
PL2W            13.18669796 W
PL12W            0.39276794 W
PL13W            0.37509048 W
SFO2            400.1716007 MHz
SI                    32768
SF              100.6228270 MHz
WDW                      EM
SSB                       0
LB                     1.00 Hz
GB                        0
PC                     1.40
```

3d ^{13}C NMR

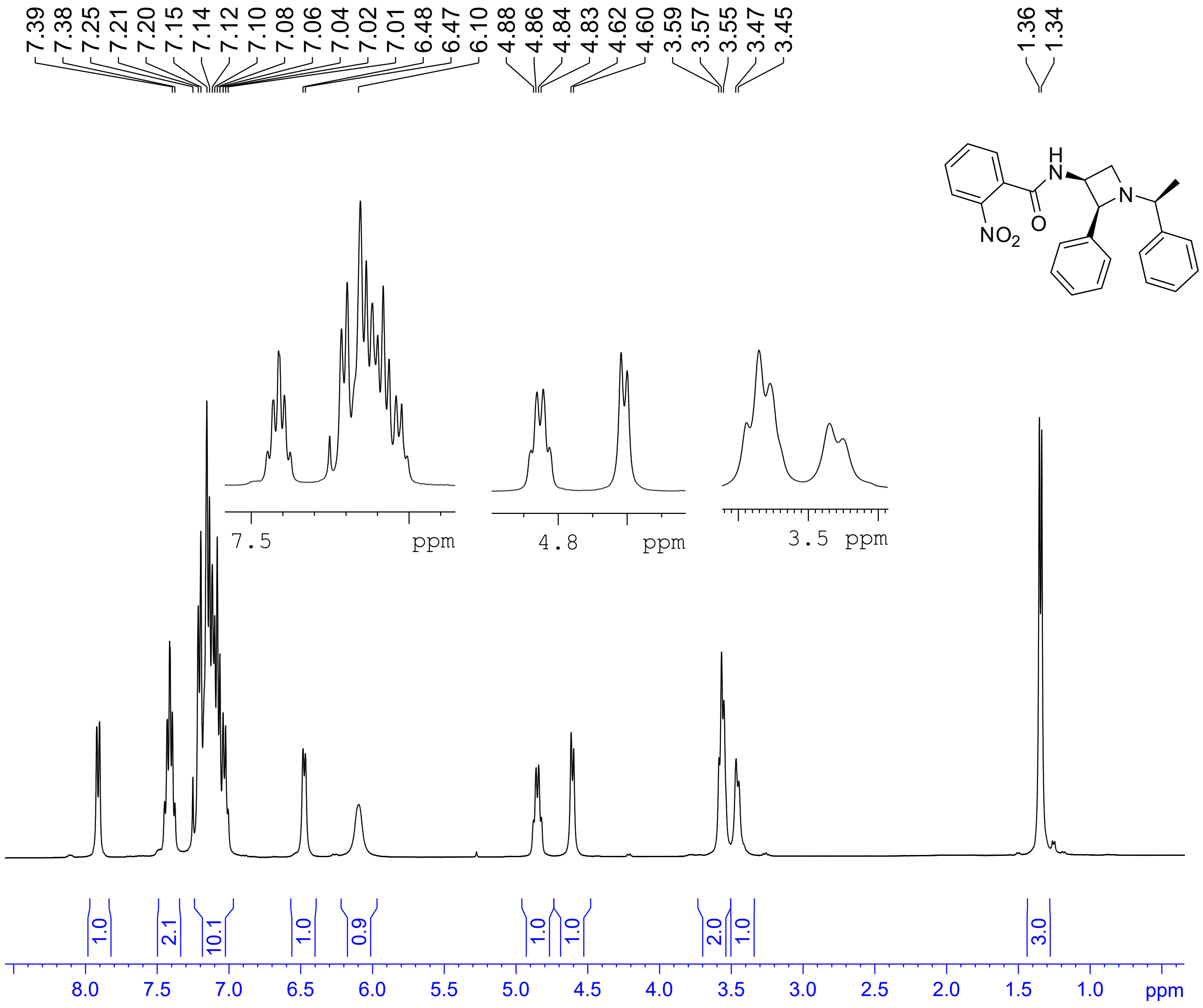

NAME	3e ZX-ONO2-CisPhamide
EXPNO	1
PROCNO	1
Date_	20150115
Time	9.26
INSTRUM	spect
PROBHD	5 mm PABBO BB-
PULPROG	zg30
TD	65536
SOLVENT	CDCl3
NS	16
DS	2
SWH	8223.685 Hz
FIDRES	0.125483 Hz
AQ	3.9846387 sec
RG	71.8
DW	60.800 usec
DE	6.50 usec
TE	293.0 K
D1	1.00000000 sec
TD0	1

======== CHANNEL f1 ========

NUC1	1H
P1	13.80 usec
PL1	-1.00 dB
PL1W	13.18669796 W
SFO1	400.1724712 MHz
SI	32768
SF	400.1700076 MHz
WDW	EM
SSB	0
LB	0.30 Hz
GB	0
PC	1.00

3e ^{1}H NMR

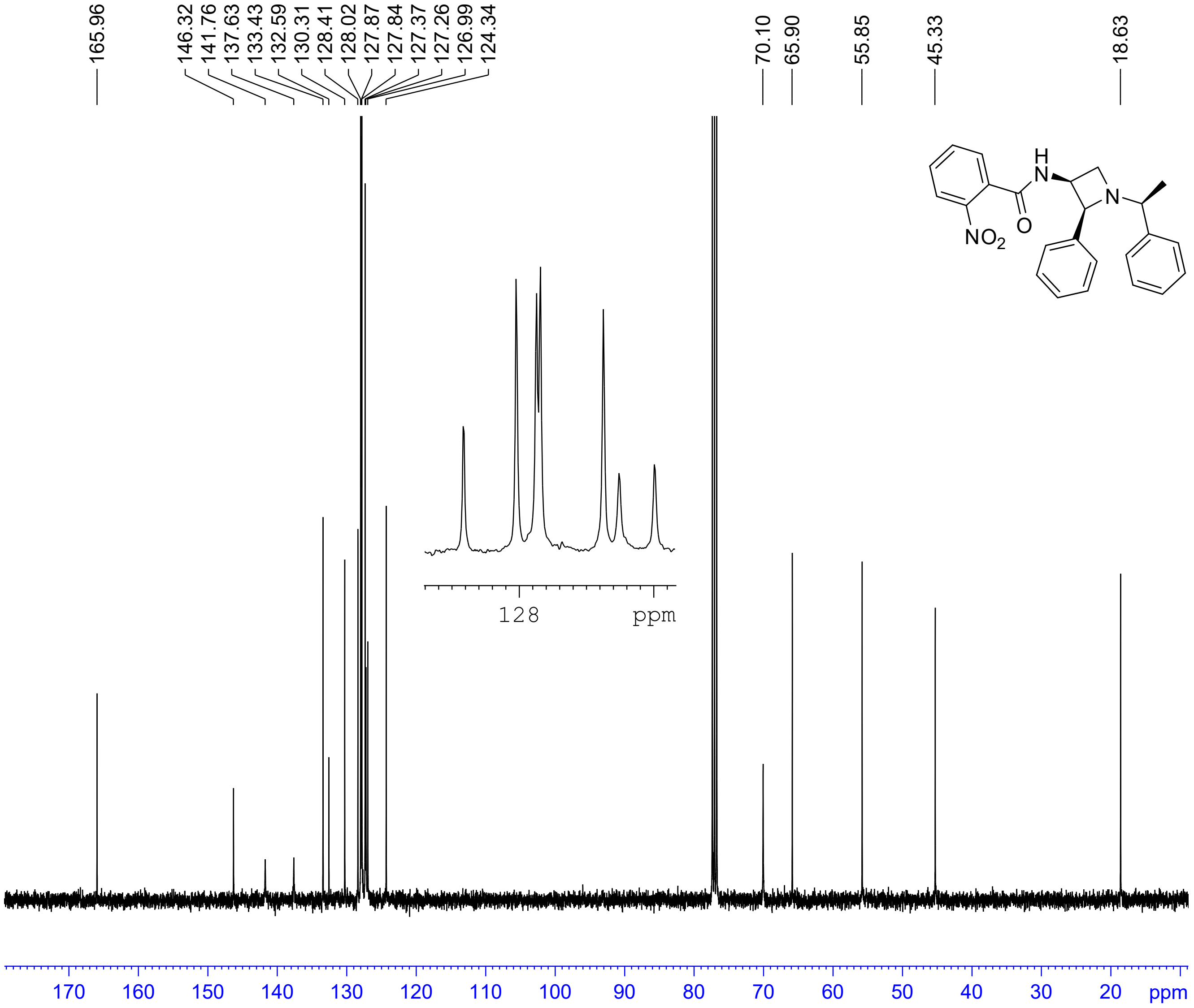


```
NAME       3e ZX-oNO2-CisPhamide-C13
EXPNO                      1
PROCNO                     1
Date_               20150116
Time                   21.49
INSTRUM                spect
PROBHD   5 mm PABBO BB-
PULPROG              zgpg30
TD                     65536
SOLVENT                CDCl3
NS                       166
DS                         4
SWH                24038.461 Hz
FIDRES              0.366798 Hz
AQ                 1.3631988 sec
RG                       203
DW                    20.800 usec
DE                      6.50 usec
TE                     292.3 K
D1                2.00000000 sec
D11               0.03000000 sec
TD0                        1

======== CHANNEL f1 ========
NUC1                     13C
P1                      8.50 usec
PL1                    -2.00 dB
PL1W             57.32743073 W
SFO1             100.6328888 MHz

======== CHANNEL f2 ========
CPDPRG2              waltz16
NUC2                      1H
PCPD2                  80.00 usec
PL2                    -1.00 dB
PL12                   14.26 dB
PL13                   14.46 dB
PL2W             13.18669796 W
PL12W             0.39276794 W
PL13W             0.37509048 W
SFO2             400.1716007 MHz
SI                     32768
SF               100.6228270 MHz
WDW                       EM
SSB                        0
LB                      1.00 Hz
GB                         0
PC                      1.40
```

3e ^{13}C NMR

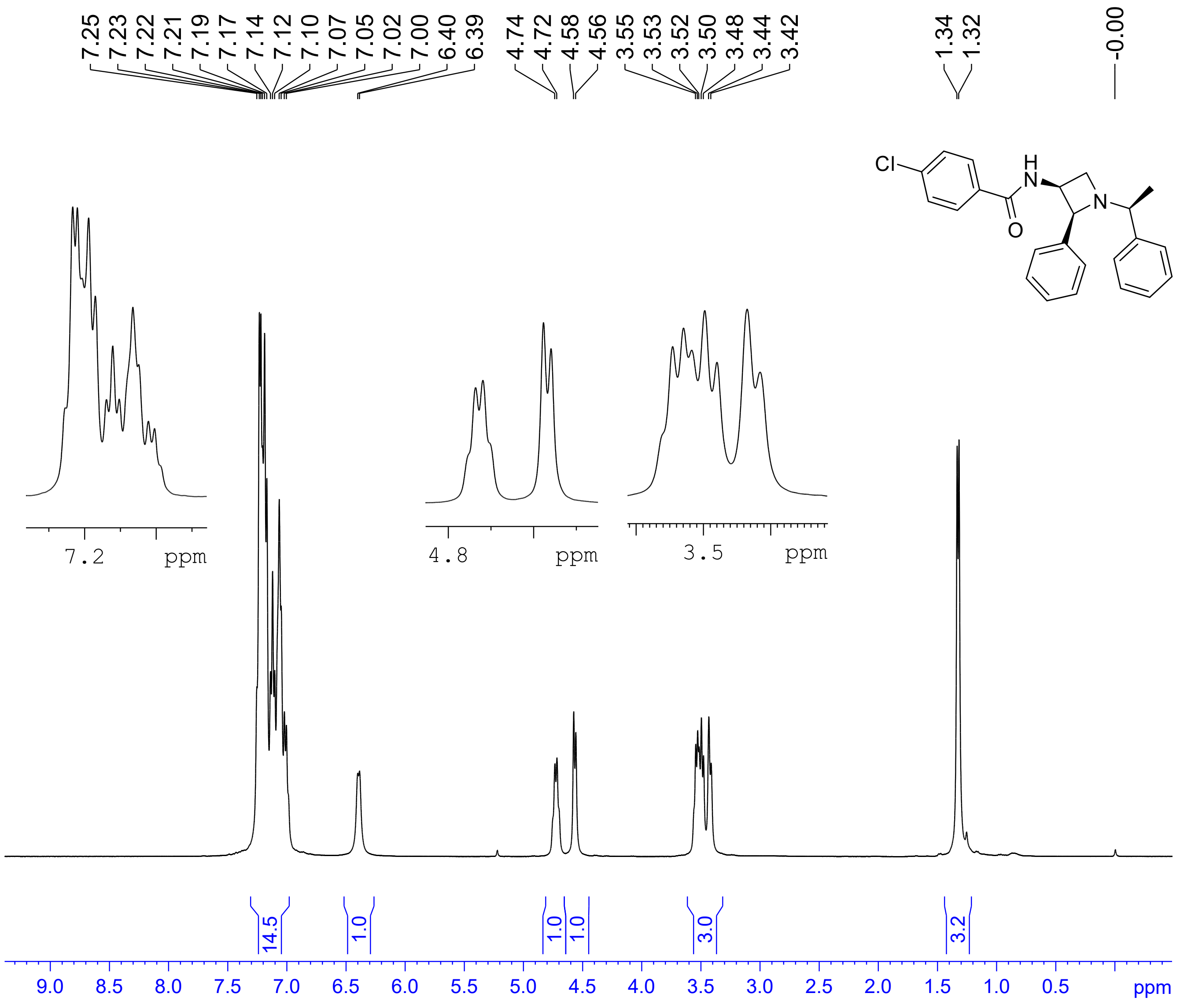

NAME	3f ZX-pCl-CisPhamide
EXPNO	1
PROCNO	1
Date_	20141111
Time	20.39
INSTRUM	spect
PROBHD	5 mm PABBO BB-
PULPROG	zg30
TD	65536
SOLVENT	CDCl3
NS	16
DS	2
SWH	8223.685 Hz
FIDRES	0.125483 Hz
AQ	3.9846387 sec
RG	28.5
DW	60.800 usec
DE	6.50 usec
TE	292.7 K
D1	1.00000000 sec
TD0	1
======== CHANNEL f1 ========	
NUC1	1H
P1	13.80 usec
PL1	-1.00 dB
PL1W	13.18669796 W
SFO1	400.1724712 MHz
SI	32768
SF	400.1700165 MHz
WDW	EM
SSB	0
LB	0.30 Hz
GB	0
PC	1.00

3f ^{1}H NMR

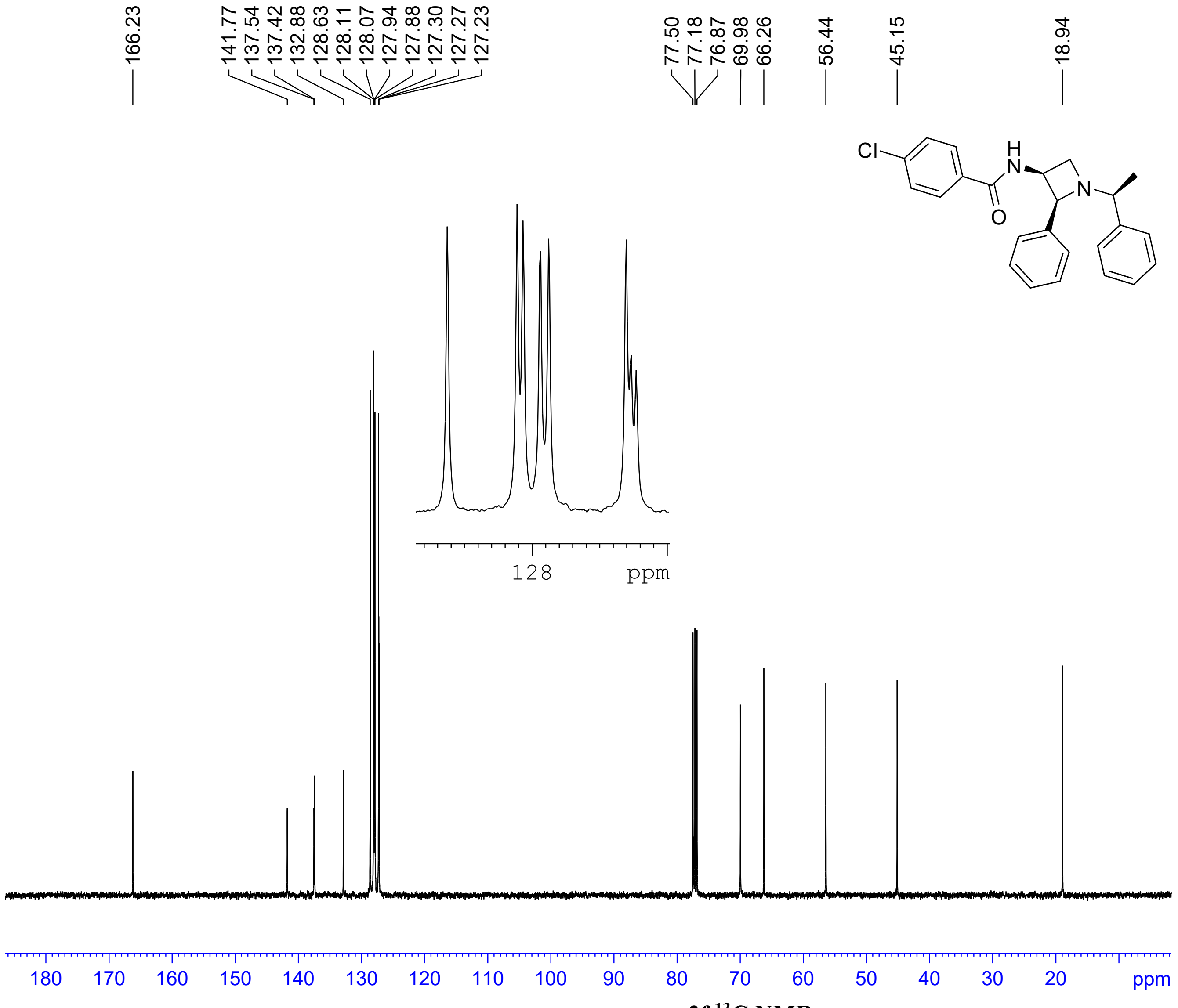

3f ^{13}C NMR

```
NAME       3f ZX-pCl-CisPhamide-C13
EXPNO                    1
PROCNO                   1
Date_             20141111
Time                 20.47
INSTRUM              spect
PROBHD   5 mm PABBO BB-
PULPROG            zgpg30
TD                   65536
SOLVENT              CDCl3
NS                     169
DS                       4
SWH              24038.461 Hz
FIDRES            0.366798 Hz
AQ               1.3631988 sec
RG                     203
DW                  20.800 usec
DE                    6.50 usec
TE                   293.6 K
D1              2.00000000 sec
D11             0.03000000 sec
TD0                      1

======== CHANNEL f1 ========
NUC1                   13C
P1                    8.50 usec
PL1                  -2.00 dB
PL1W           57.32743073 W
SFO1           100.6328888 MHz

======== CHANNEL f2 ========
CPDPRG2            waltz16
NUC2                    1H
PCPD2                80.00 usec
PL2                  -1.00 dB
PL12                 14.26 dB
PL13                 14.46 dB
PL2W           13.18669796 W
PL12W           0.39276794 W
PL13W           0.37509048 W
SFO2           400.1716007 MHz
SI                   32768
SF             100.6228270 MHz
WDW                     EM
SSB                      0
LB                    1.00 Hz
GB                       0
PC                    1.40
```

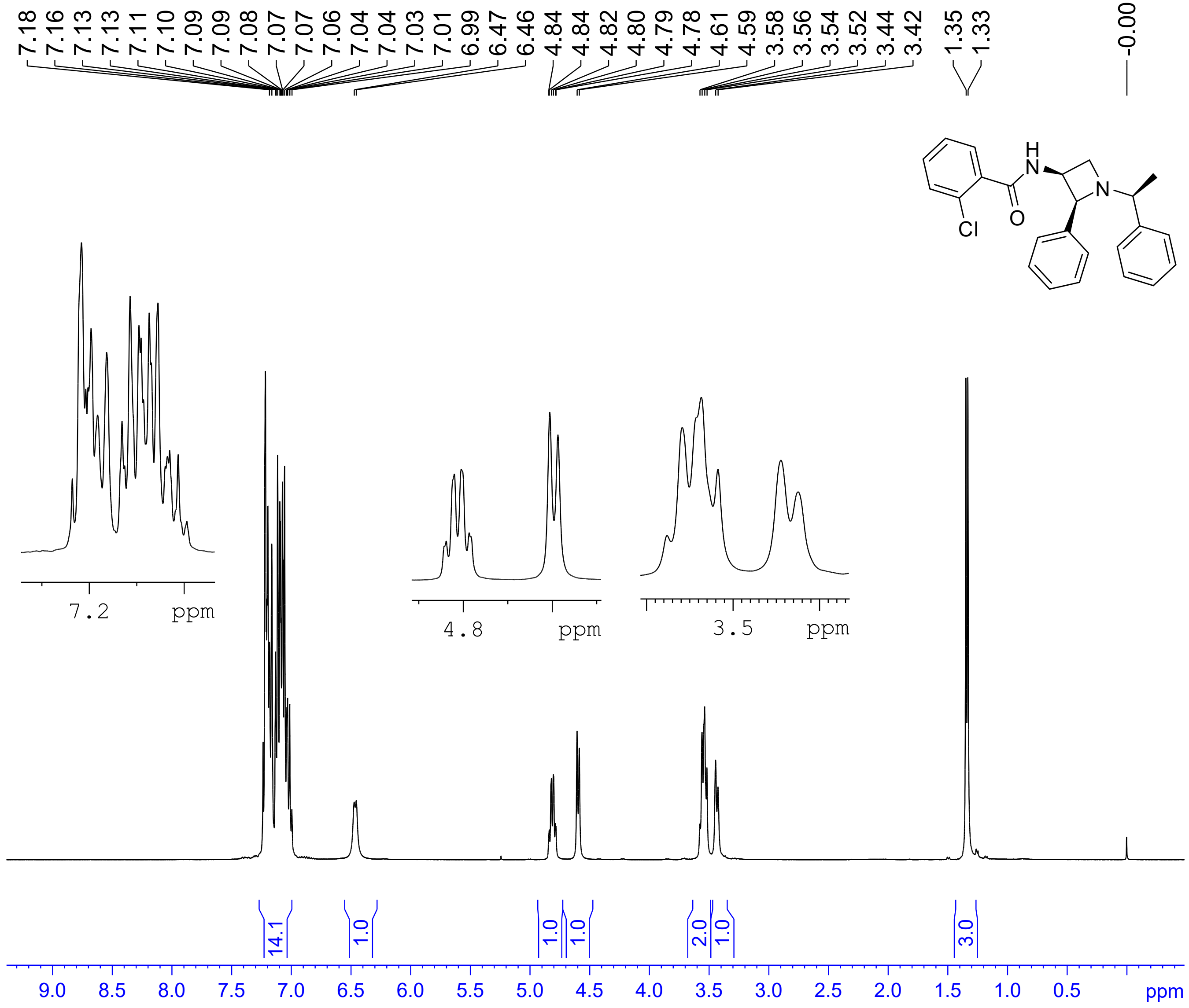

3g ^{1}H NMR

BRUKER

NAME	3g ZX-oCl-CisPhamide	
EXPNO	1	
PROCNO	1	
Date_	20150116	
Time	21.36	
INSTRUM	spect	
PROBHD	5 mm PABBO BB-	
PULPROG	zg30	
TD	65536	
SOLVENT	CDCl3	
NS	16	
DS	2	
SWH	8223.685	Hz
FIDRES	0.125483	Hz
AQ	3.9846387	sec
RG	32	
DW	60.800	usec
DE	6.50	usec
TE	291.5	K
D1	1.00000000	sec
TD0	1	

======== CHANNEL f1 ========

NUC1	1H	
P1	13.80	usec
PL1	-1.00	dB
PL1W	13.18669796	W
SFO1	400.1724712	MHz
SI	32768	
SF	400.1700136	MHz
WDW	EM	
SSB	0	
LB	0.30	Hz
GB	0	
PC	1.00	

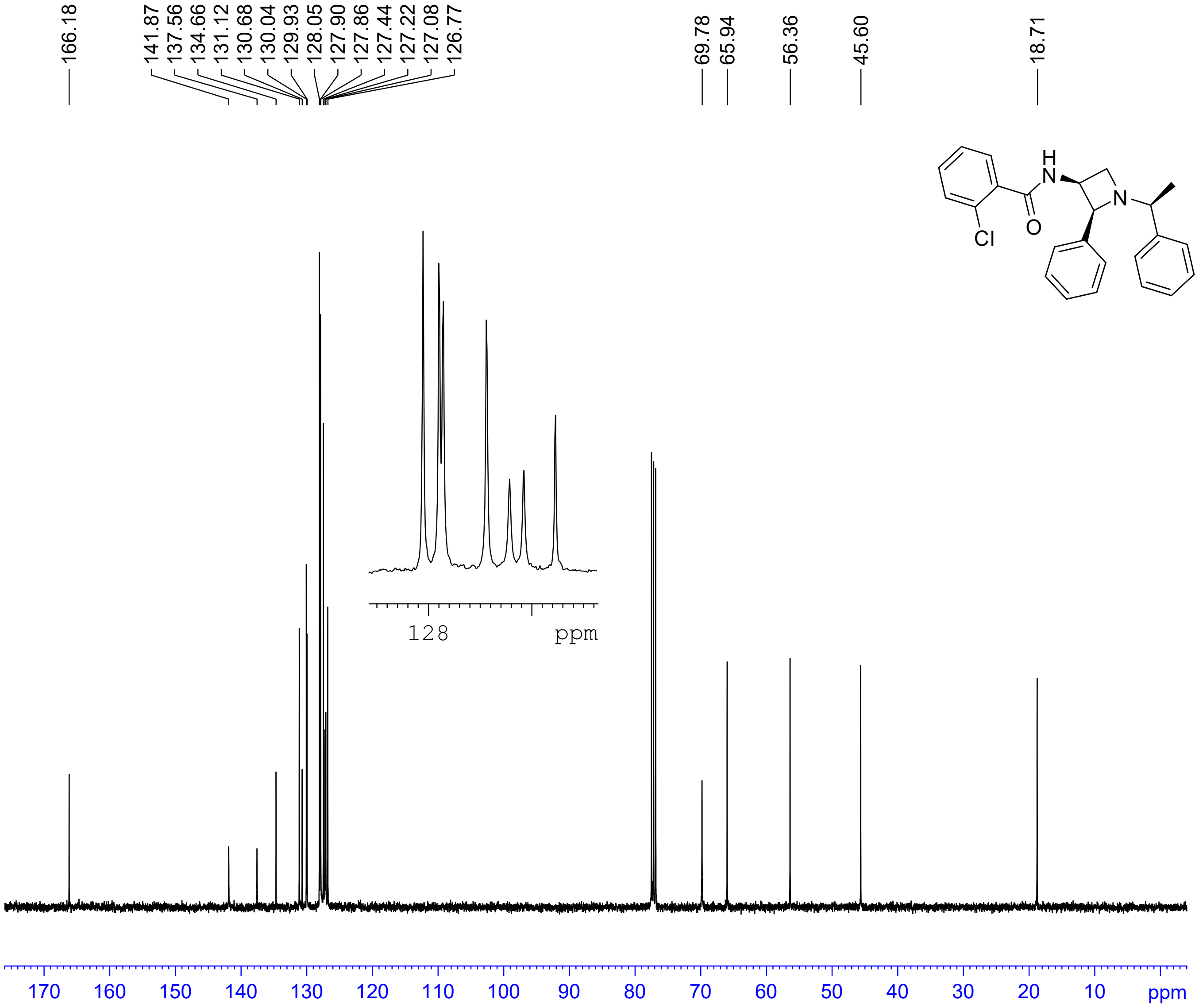

NAME	3g ZX-oCl-CisPhamide-C13	
EXPNO	1	
PROCNO	1	
Date_	20150116	
Time	21.40	
INSTRUM	spect	
PROBHD	5 mm PABBO BB-	
PULPROG	zgpg30	
TD	65536	
SOLVENT	CDCl3	
NS	117	
DS	4	
SWH	24038.461	Hz
FIDRES	0.366798	Hz
AQ	1.3631988	sec
RG	203	
DW	20.800	usec
DE	6.50	usec
TE	292.4	K
D1	2.00000000	sec
D11	0.03000000	sec
TD0	1	

======== CHANNEL f1 ========

NUC1	13C	
P1	8.50	usec
PL1	-2.00	dB
PL1W	57.32743073	W
SFO1	100.6328888	MHz

======== CHANNEL f2 ========

CPDPRG2	waltz16	
NUC2	1H	
PCPD2	80.00	usec
PL2	-1.00	dB
PL12	14.26	dB
PL13	14.46	dB
PL2W	13.18669796	W
PL12W	0.39276794	W
PL13W	0.37509048	W
SFO2	400.1716007	MHz
SI	32768	
SF	100.6228270	MHz
WDW	EM	
SSB	0	
LB	1.00	Hz
GB	0	
PC	1.40	

3g ^{13}C NMR

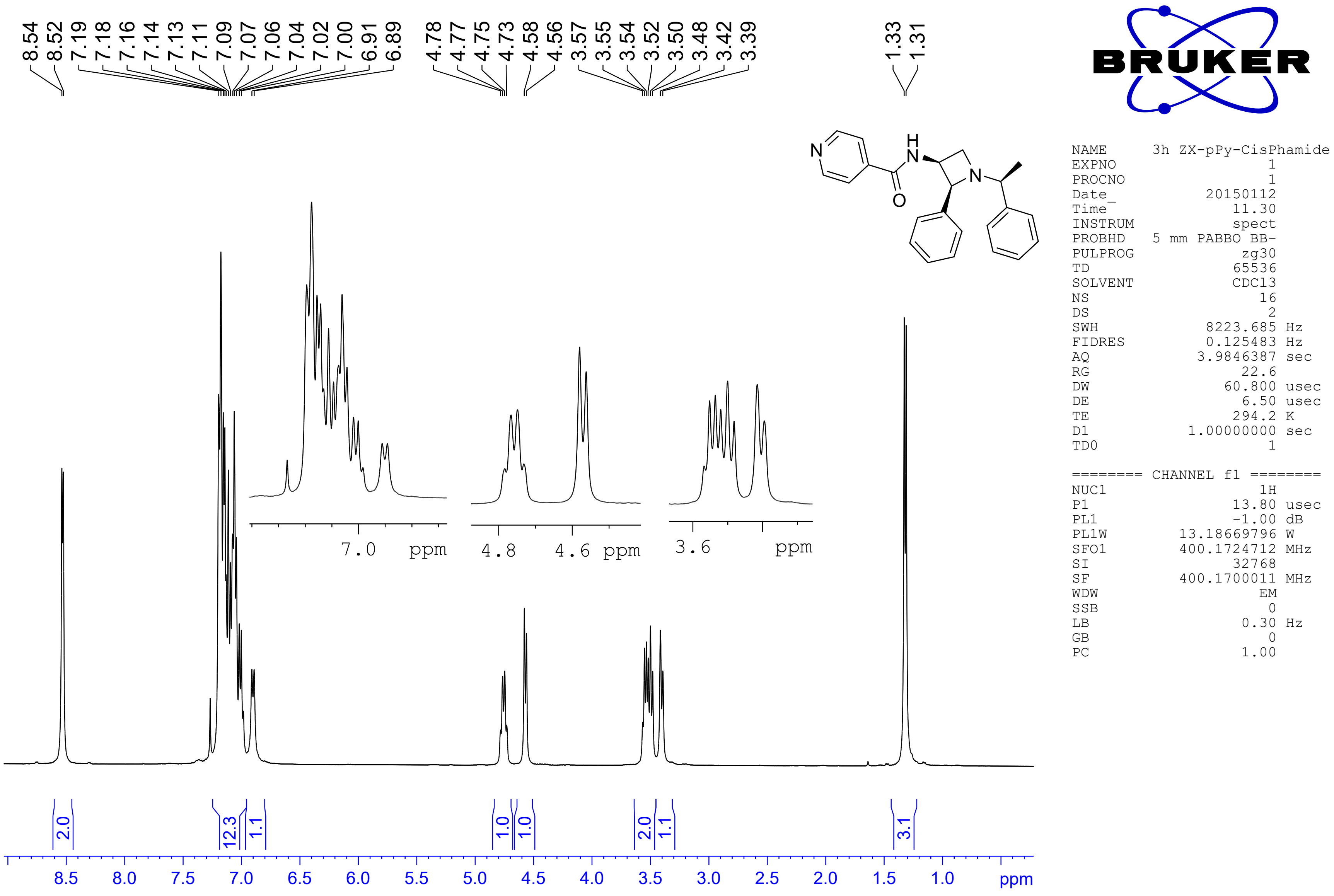
8.54 8.52 7.19 7.18 7.16 7.14 7.13 7.11 7.09 7.07 7.06 7.04 7.02 7.00 6.91 6.89
4.78 4.77 4.75 4.73 4.58 4.56 3.57 3.55 3.54 3.52 3.50 3.48 3.42 3.39
1.33 1.31
BRUKER
N
H
N
N
O
7.0 ppm
4.8 4.6 ppm
3.6 ppm
2.0 12.3 1.1 1.0 1.0 2.0 1.1 3.1
8.5 8.0 7.5 7.0 6.5 6.0 5.5 5.0 4.5 4.0 3.5 3.0 2.5 2.0 1.5 1.0 ppm
NAME 3h ZX-pPy-CisPhamide
EXPNO 1
PROCNO 1
Date_ 20150112
Time 11.30
INSTRUM spect
PROBHD 5 mm PABBO BB-
PULPROG zg30
TD 65536
SOLVENT CDCl3
NS 16
DS 2
SWH 8223.685 Hz
FIDRES 0.125483 Hz
AQ 3.9846387 sec
RG 22.6
DW 60.800 usec
DE 6.50 usec
TE 294.2 K
D1 1.00000000 sec
TD0 1
======== CHANNEL f1 ========
NUC1 1H
P1 13.80 usec
PL1 -1.00 dB
PL1W 13.18669796 W
SFO1 400.1724712 MHz
SI 32768
SF 400.1700011 MHz
WDW EM
SSB 0
LB 0.30 Hz
GB 0
PC 1.00

3h ^{1}H NMR

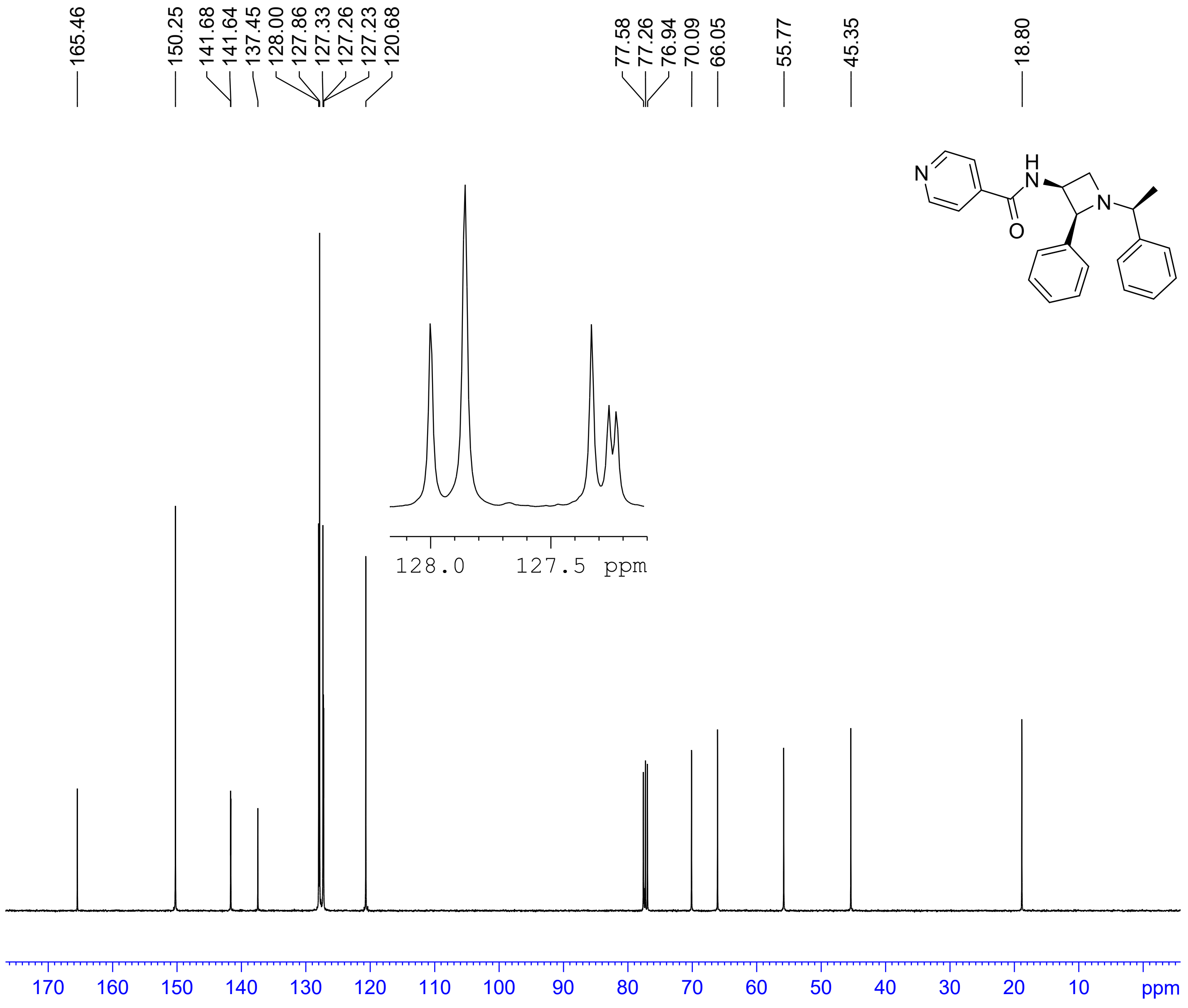


```
NAME      3h ZX-pPy-CisPhamide-C13
EXPNO                  1
PROCNO                 1
Date_           20150112
Time               14.06
INSTRUM            spect
PROBHD    5 mm PABBO BB-
PULPROG          zgpg30
TD                 65536
SOLVENT            CDCl3
NS                   512
DS                     4
SWH          24038.461 Hz
FIDRES        0.366798 Hz
AQ           1.3631988 sec
RG                   203
DW                20.800 usec
DE                  6.50 usec
TE                 296.7 K
D1            2.00000000 sec
D11           0.03000000 sec
TD0                    1

======== CHANNEL f1 ========
NUC1                 13C
P1                  8.50 usec
PL1                -2.00 dB
PL1W         57.32743073 W
SFO1         100.6328888 MHz

======== CHANNEL f2 ========
CPDPRG2          waltz16
NUC2                  1H
PCPD2              80.00 usec
PL2                -1.00 dB
PL12               14.26 dB
PL13               14.46 dB
PL2W         13.18669796 W
PL12W         0.39276794 W
PL13W         0.37509048 W
SFO2         400.1716007 MHz
SI                 32768
SF           100.6228270 MHz
WDW                   EM
SSB                    0
LB                  1.00 Hz
GB                     0
PC                  1.40
```

3h ^{13}C NMR

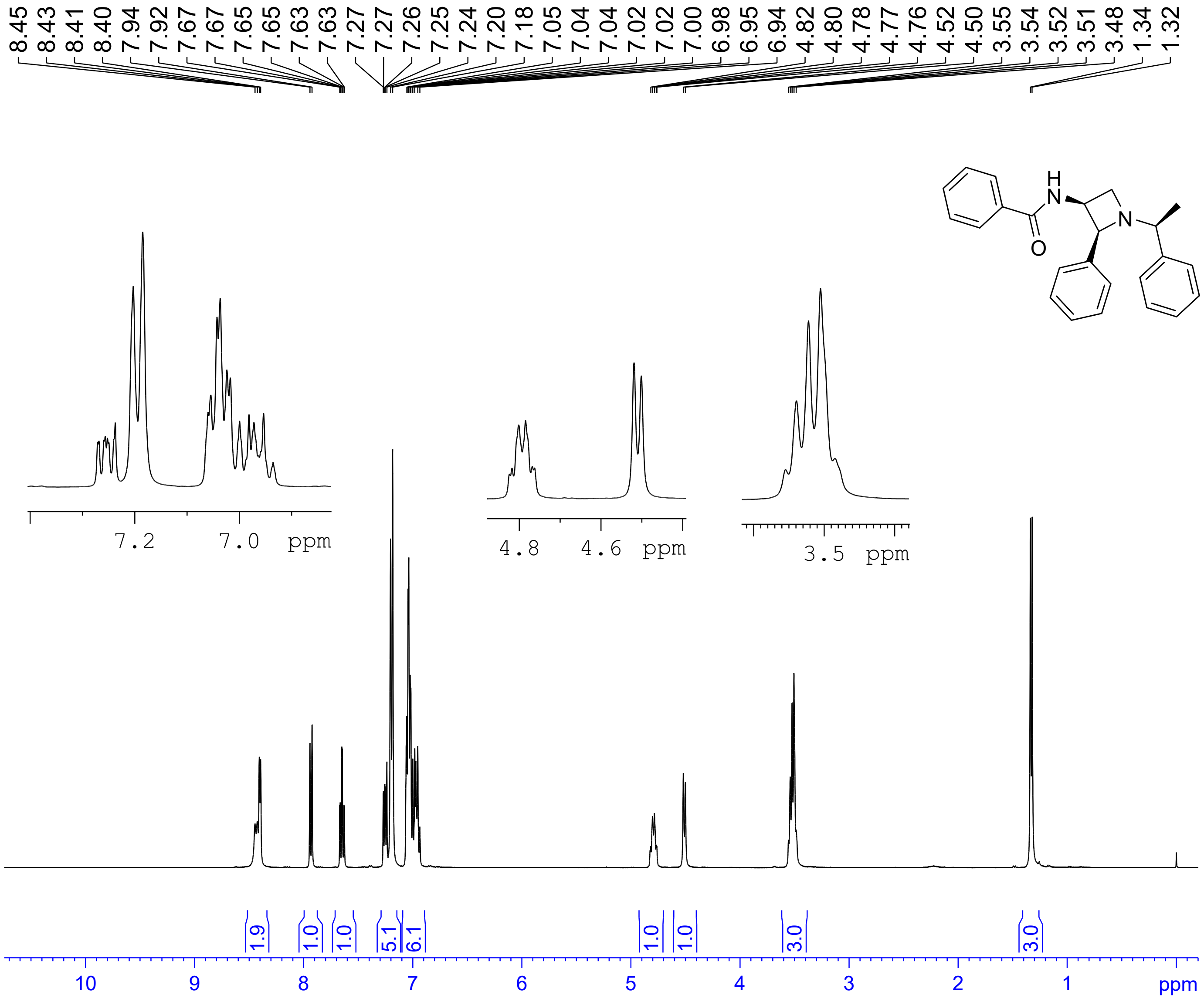

3i ^{1}H NMR

```
NAME       3i ZX-oPy-CisPhamide
EXPNO                 1
PROCNO                1
Date_          20150116
Time               9.40
INSTRUM           spect
PROBHD   5 mm PABBO BB-
PULPROG            zg30
TD                65536
SOLVENT           CDCl3
NS                   16
DS                    2
SWH            8223.685 Hz
FIDRES         0.125483 Hz
AQ            3.9846387 sec
RG                 28.5
DW               60.800 usec
DE                 6.50 usec
TE                292.4 K
D1           1.00000000 sec
TD0                   1

======== CHANNEL f1 ========
NUC1                 1H
P1                13.80 usec
PL1               -1.00 dB
PL1W        13.18669796 W
SFO1        400.1724712 MHz
SI                32768
SF          400.1700129 MHz
WDW                  EM
SSB                   0
LB                 0.30 Hz
GB                    0
PC                 1.00
```

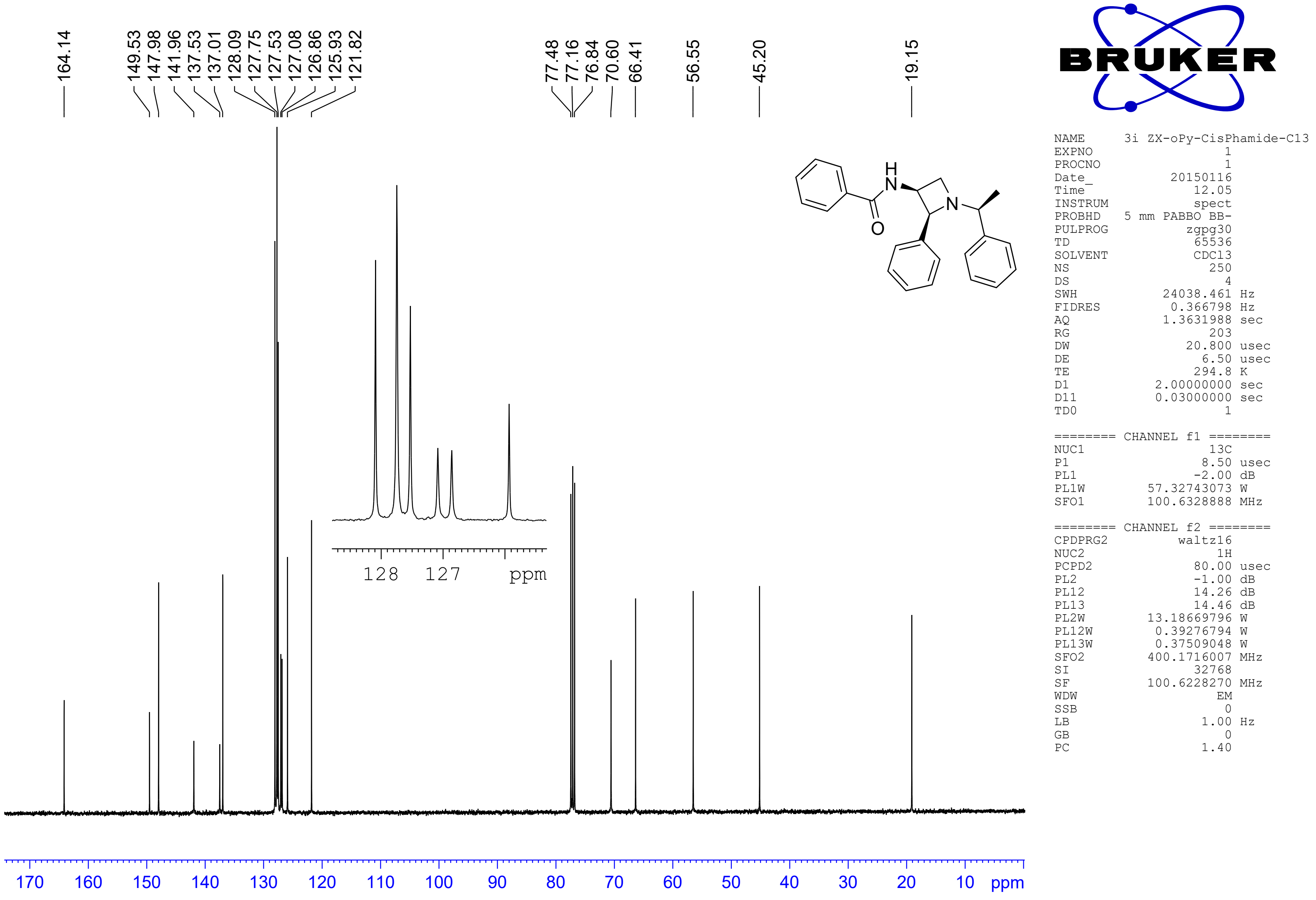
164.14
149.53
147.98
141.96
137.53
137.01
128.09
127.75
127.53
127.08
126.86
125.93
121.82
77.48
77.16
76.84
70.60
66.41
56.55
45.20
19.15
128
127
ppm
170 160 150 140 130 120 110 100 90 80 70 60 50 40 30 20 10 ppm
H
N
O
N
BRUKER
NAME 3i ZX-oPy-CisPhamide-C13
EXPNO 1
PROCNO 1
Date_ 20150116
Time 12.05
INSTRUM spect
PROBHD 5 mm PABBO BB-
PULPROG zgpg30
TD 65536
SOLVENT CDCl3
NS 250
DS 4
SWH 24038.461 Hz
FIDRES 0.366798 Hz
AQ 1.3631988 sec
RG 203
DW 20.800 usec
DE 6.50 usec
TE 294.8 K
D1 2.00000000 sec
D11 0.03000000 sec
TD0 1
======== CHANNEL f1 ========
NUC1 13C
P1 8.50 usec
PL1 -2.00 dB
PL1W 57.32743073 W
SFO1 100.6328888 MHz
======== CHANNEL f2 ========
CPDPRG2 waltz16
NUC2 1H
PCPD2 80.00 usec
PL2 -1.00 dB
PL12 14.26 dB
PL13 14.46 dB
PL2W 13.18669796 W
PL12W 0.39276794 W
PL13W 0.37509048 W
SFO2 400.1716007 MHz
SI 32768
SF 100.6228270 MHz
WDW EM
SSB 0
LB 1.00 Hz
GB 0
PC 1.40

3i ^{13}C NMR

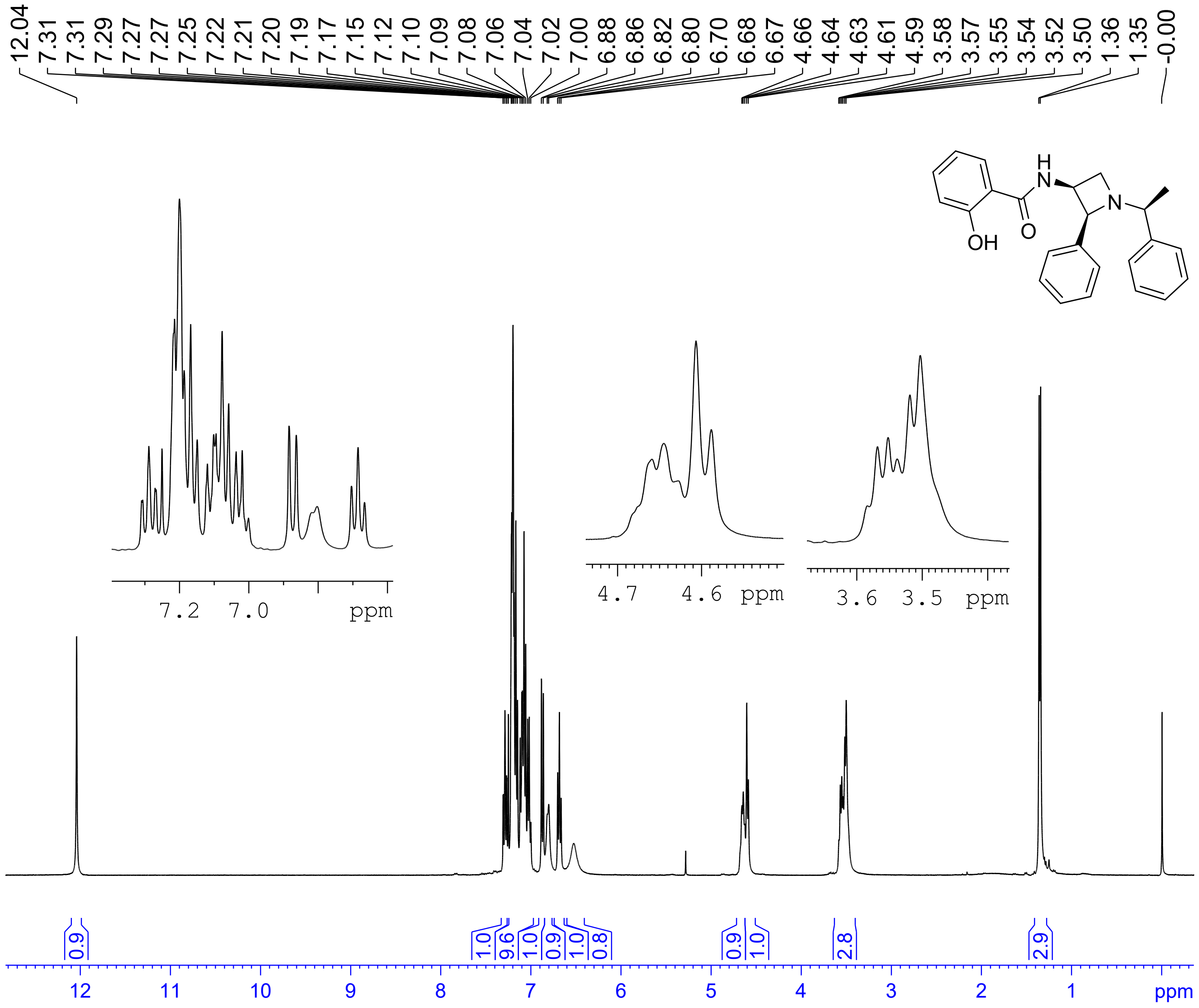

3j ¹H NMR

```
NAME      3j ZX-oOH-CisPhamide-1
EXPNO                    1
PROCNO                   1
Date_             20141126
Time                 20.36
INSTRUM              spect
PROBHD   5 mm PABBO BB-
PULPROG               zg30
TD                   65536
SOLVENT              CDCl3
NS                      16
DS                       2
SWH              8223.685 Hz
FIDRES           0.125483 Hz
AQ              3.9846387 sec
RG                     114
DW                  60.800 usec
DE                    6.50 usec
TE                   295.3 K
D1              1.00000000 sec
TD0                      1

======== CHANNEL f1 ========
NUC1                    1H
P1                   13.80 usec
PL1                  -1.00 dB
PL1W           13.18669796 W
SFO1           400.1724712 MHz
SI                   32768
SF             400.1700078 MHz
WDW                     EM
SSB                      0
LB                    0.30 Hz
GB                       0
PC                    1.00
```

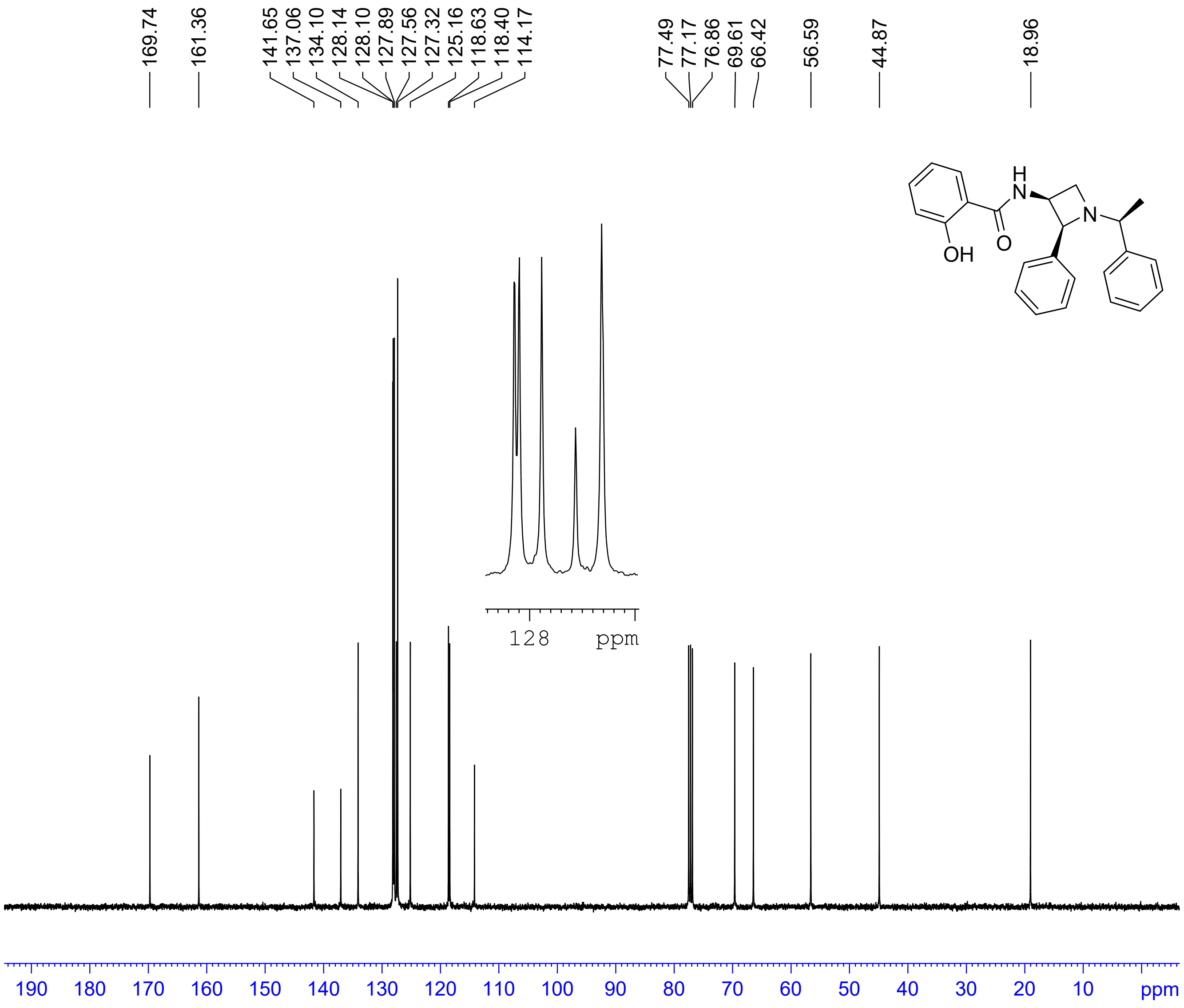

3j ^{13}C NMR

```
NAME        3j ZX-oOH-CisPhamide-C13
EXPNO                    1
PROCNO                   1
Date_             20141125
Time                 17.12
INSTRUM              spect
PROBHD      5 mm PABBO BB-
PULPROG            zgpg30
TD                  65536
SOLVENT             CDCl3
NS                    176
DS                      4
SWH             24038.461 Hz
FIDRES           0.366798 Hz
AQ              1.3631988 sec
RG                    203
DW                 20.800 usec
DE                   6.50 usec
TE                  295.8 K
D1             2.00000000 sec
D11            0.03000000 sec
TD0                     1

======== CHANNEL f1 ========
NUC1                  13C
P1                   8.50 usec
PL1                 -2.00 dB
PL1W          57.32743073 W
SFO1         100.6328888 MHz

======== CHANNEL f2 ========
CPDPRG2           waltz16
NUC2                   1H
PCPD2               80.00 usec
PL2                 -1.00 dB
PL12                14.26 dB
PL13                14.46 dB
PL2W          13.18669796 W
PL12W          0.39276794 W
PL13W          0.37509048 W
SFO2         400.1716007 MHz
SI                  32768
SF           100.6228270 MHz
WDW                    EM
SSB                     0
LB                   1.00 Hz
GB                      0
PC                   1.40
```

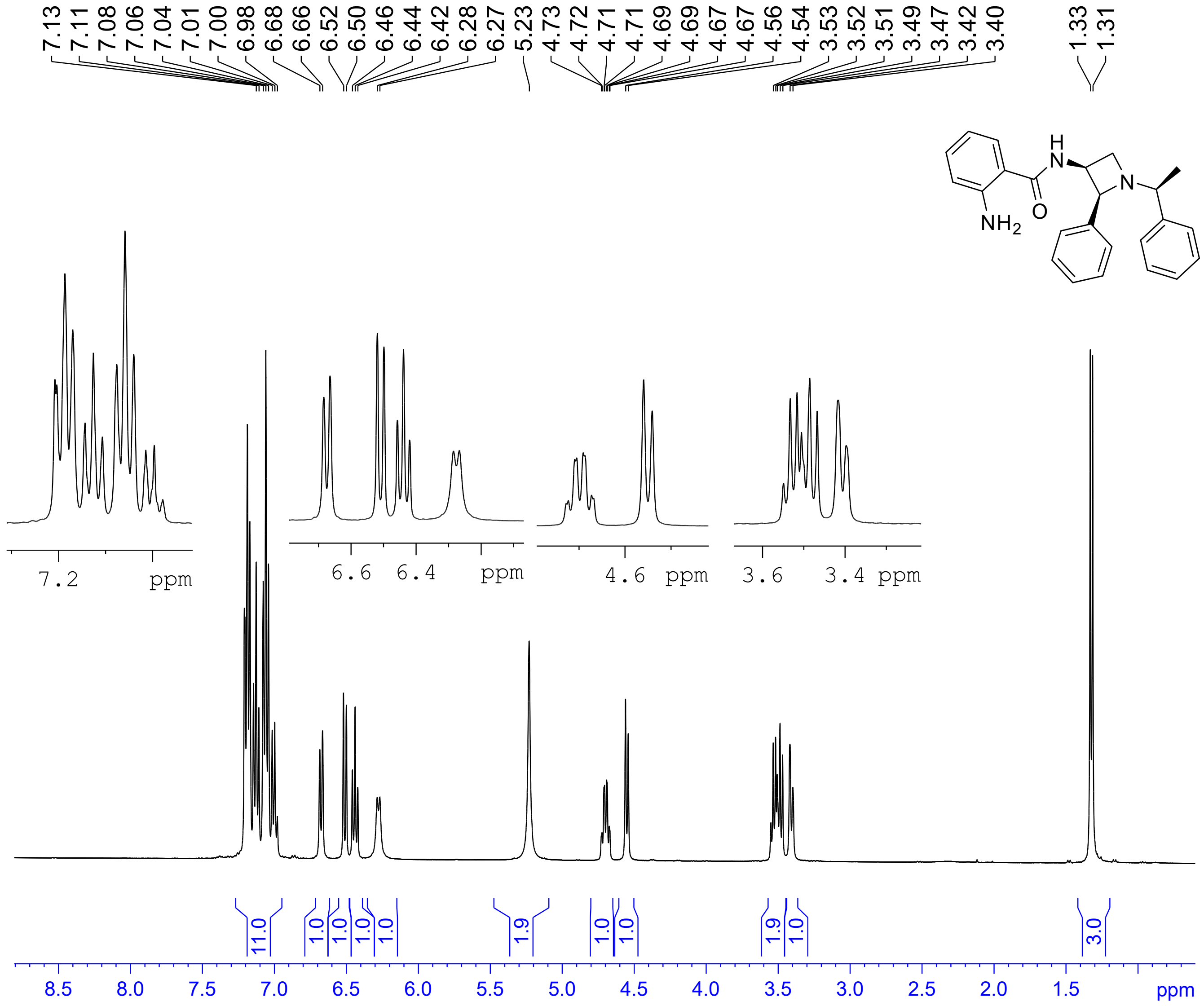

NAME	3k ZX-oNH2-CisPhamide	
EXPNO	1	
PROCNO	1	
Date_	20150114	
Time	10.01	
INSTRUM	spect	
PROBHD	5 mm PABBO BB-	
PULPROG	zg30	
TD	65536	
SOLVENT	CDCl3	
NS	16	
DS	2	
SWH	8223.685	Hz
FIDRES	0.125483	Hz
AQ	3.9846387	sec
RG	28.5	
DW	60.800	usec
DE	6.50	usec
TE	292.8	K
D1	1.00000000	sec
TD0	1	
======== CHANNEL f1 ========		
NUC1	1H	
P1	13.80	usec
PL1	-1.00	dB
PL1W	13.18669796	W
SFO1	400.1724712	MHz
SI	32768	
SF	400.1700242	MHz
WDW	EM	
SSB	0	
LB	0.30	Hz
GB	0	
PC	1.00	

3k ^{1}H NMR

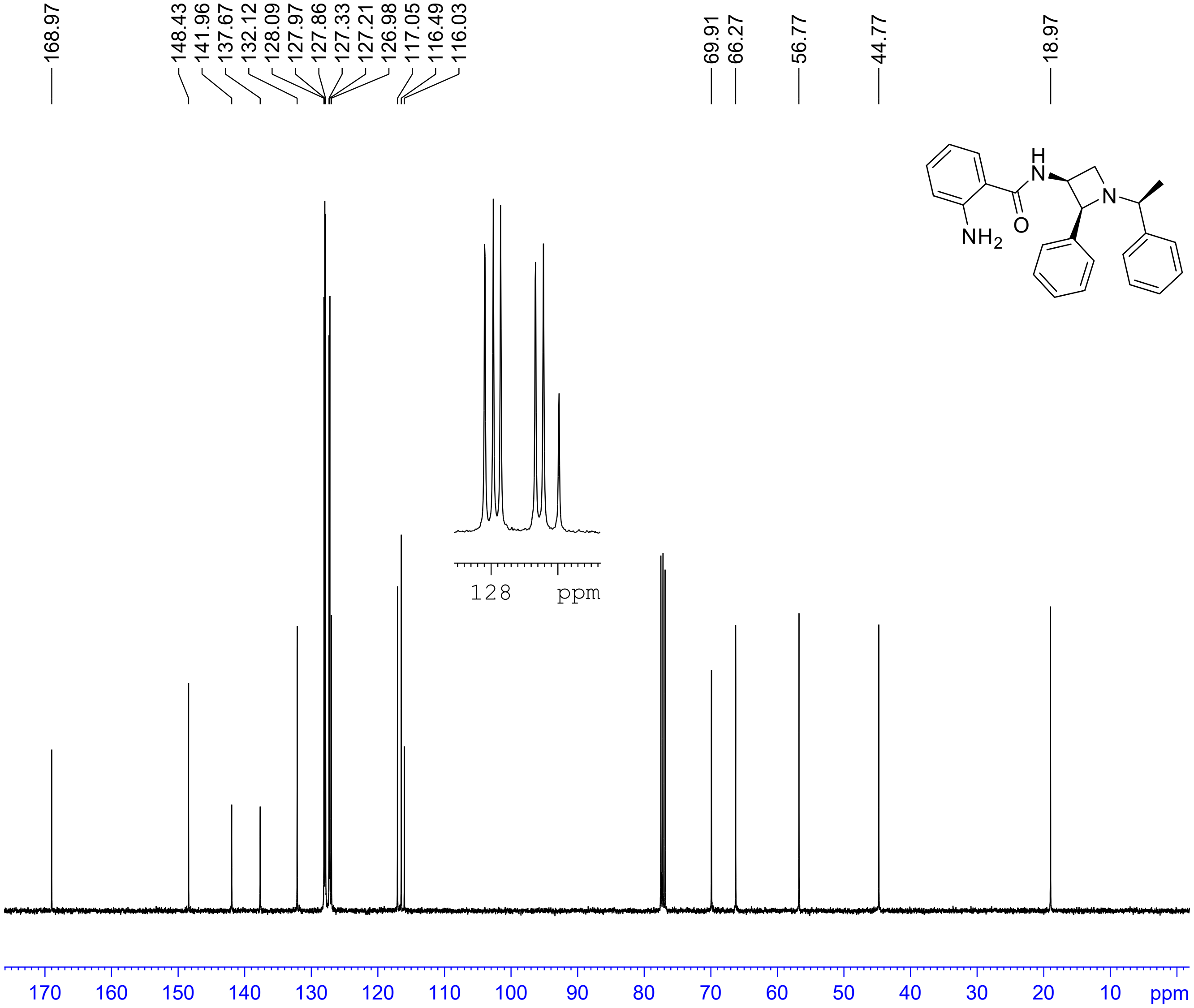

NAME	3k ZX-oNH2-CisPhamide-C13
EXPNO	1
PROCNO	1
Date_	20150114
Time	12.49
INSTRUM	spect
PROBHD	5 mm PABBO BB-
PULPROG	zgpg30
TD	65536
SOLVENT	CDCl3
NS	250
DS	4
SWH	24038.461 Hz
FIDRES	0.366798 Hz
AQ	1.3631988 sec
RG	203
DW	20.800 usec
DE	6.50 usec
TE	294.3 K
D1	2.00000000 sec
D11	0.03000000 sec
TD0	1
======== CHANNEL f1 ========	
NUC1	13C
P1	8.50 usec
PL1	-2.00 dB
PL1W	57.32743073 W
SFO1	100.6328888 MHz
======== CHANNEL f2 ========	
CPDPRG2	waltz16
NUC2	1H
PCPD2	80.00 usec
PL2	-1.00 dB
PL12	14.26 dB
PL13	14.46 dB
PL2W	13.18669796 W
PL12W	0.39276794 W
PL13W	0.37509048 W
SFO2	400.1716007 MHz
SI	32768
SF	100.6228270 MHz
WDW	EM
SSB	0
LB	1.00 Hz
GB	0
PC	1.40

3k ^{13}C NMR

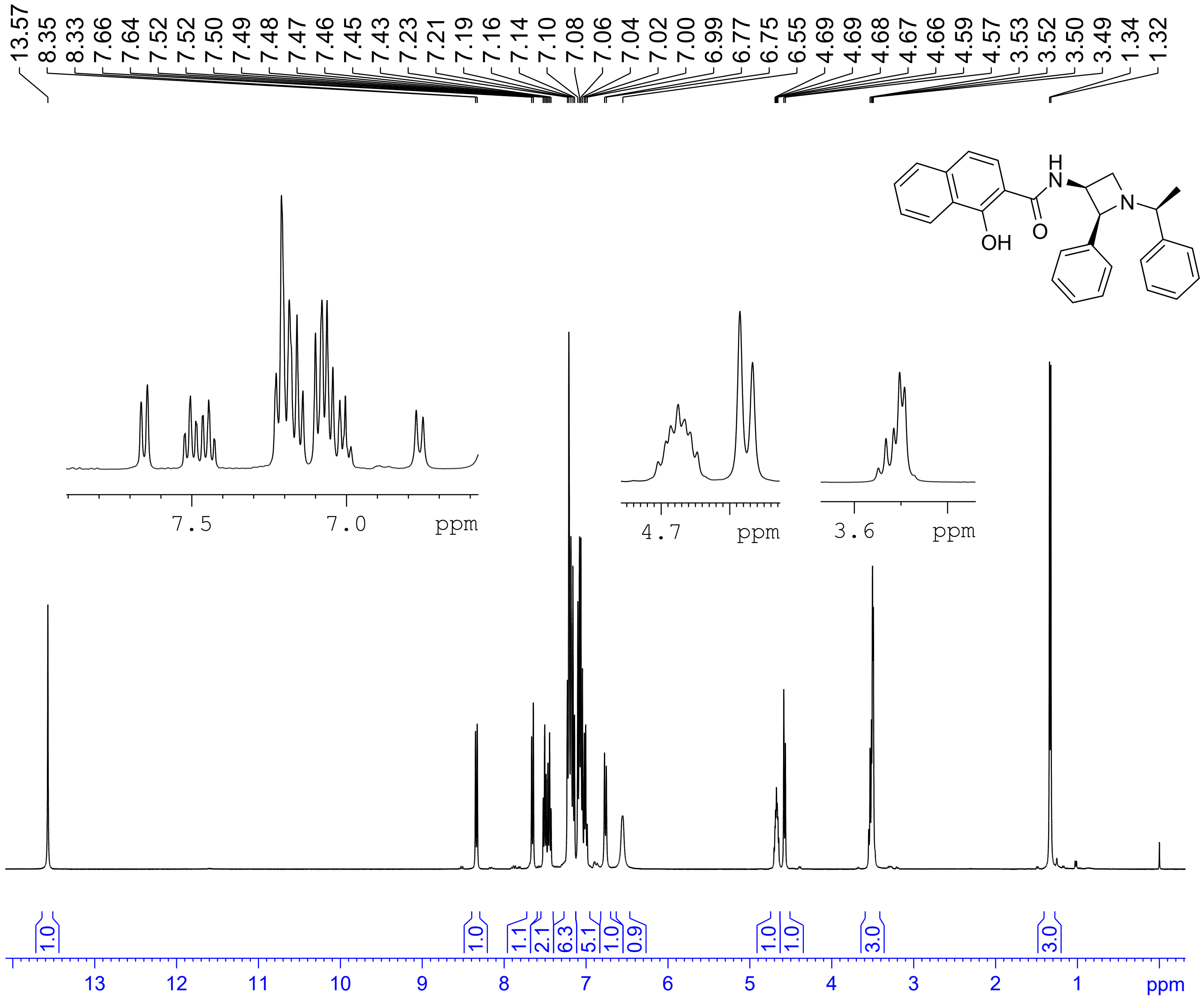

3l ¹H NMR

```
NAME       3l ZX-1OH-2naphCisamide
EXPNO                    1
PROCNO                   1
Date_             20141202
Time                 16.52
INSTRUM              spect
PROBHD     5 mm PABBO BB-
PULPROG               zg30
TD                   65536
SOLVENT              CDCl3
NS                      16
DS                       2
SWH               8223.685 Hz
FIDRES            0.125483 Hz
AQ               3.9846387 sec
RG                      36
DW                  60.800 usec
DE                    6.50 usec
TE                   291.9 K
D1              1.00000000 sec
TD0                      1

======== CHANNEL f1 ========
NUC1                    1H
P1                   13.80 usec
PL1                  -1.00 dB
PL1W           13.18669796 W
SFO1           400.1724712 MHz
SI                   32768
SF             400.1700234 MHz
WDW                     EM
SSB                      0
LB                    0.30 Hz
GB                       0
PC                    1.00
```

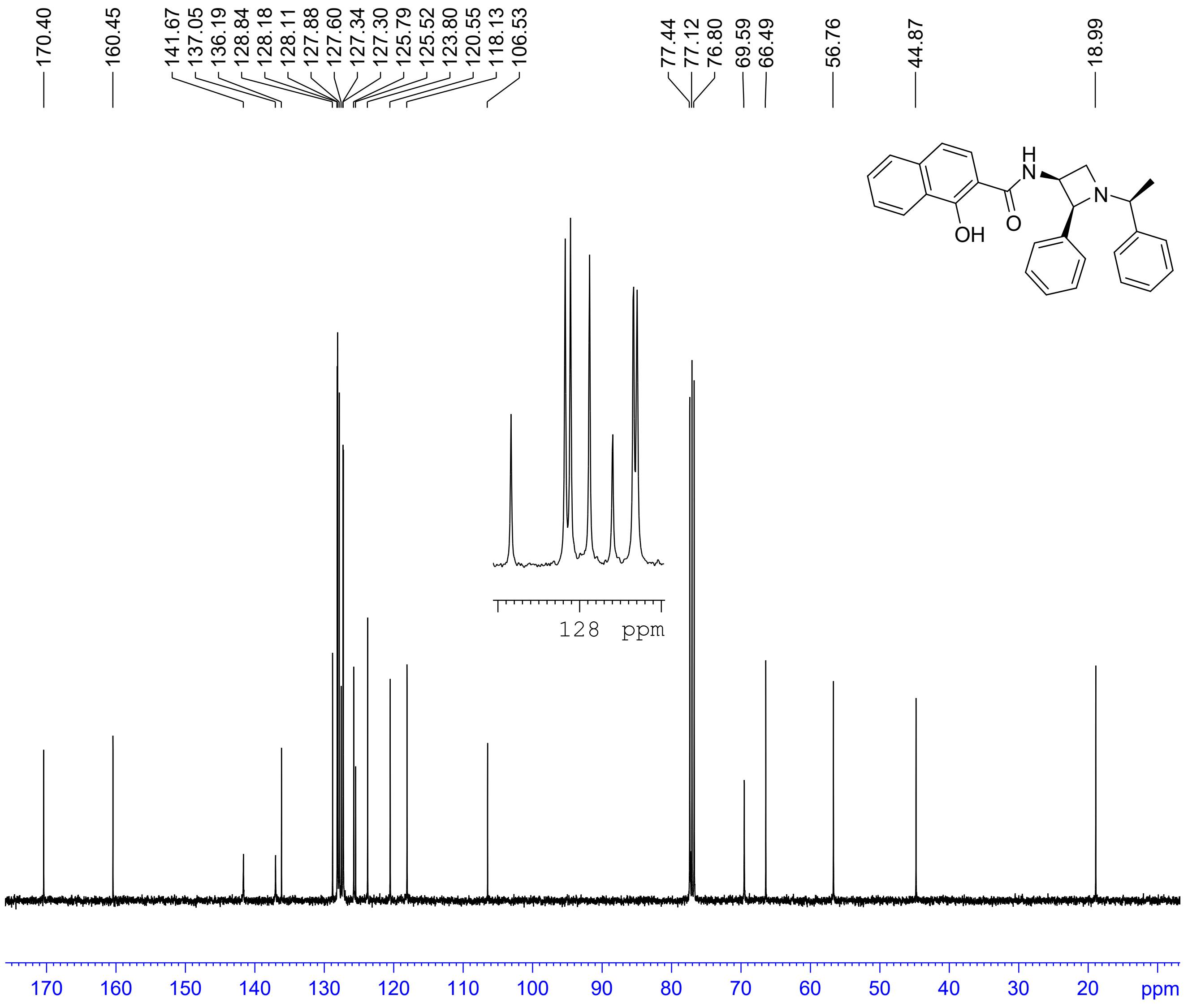

BRUKER

```
NAME       3l ZX-1OH-2naphCisamide-C13
EXPNO                1
PROCNO               1
Date_         20141202
Time             16.58
INSTRUM          spect
PROBHD   5 mm PABBO BB-
PULPROG         zgpg30
TD               65536
SOLVENT          CDCl3
NS                 178
DS                   4
SWH          24038.461 Hz
FIDRES        0.366798 Hz
AQ           1.3631988 sec
RG                 203
DW              20.800 usec
DE                6.50 usec
TE               292.6 K
D1          2.00000000 sec
D11         0.03000000 sec
TD0                  1

======== CHANNEL f1 ========
NUC1               13C
P1                8.50 usec
PL1              -2.00 dB
PL1W       57.32743073 W
SFO1       100.6328888 MHz

======== CHANNEL f2 ========
CPDPRG2        waltz16
NUC2                1H
PCPD2            80.00 usec
PL2              -1.00 dB
PL12             14.26 dB
PL13             14.46 dB
PL2W       13.18669796 W
PL12W       0.39276794 W
PL13W       0.37509048 W
SFO2       400.1716007 MHz
SI               32768
SF         100.6228270 MHz
WDW                 EM
SSB                  0
LB                1.00 Hz
GB                   0
PC                1.40
```

3l ^{13}C NMR

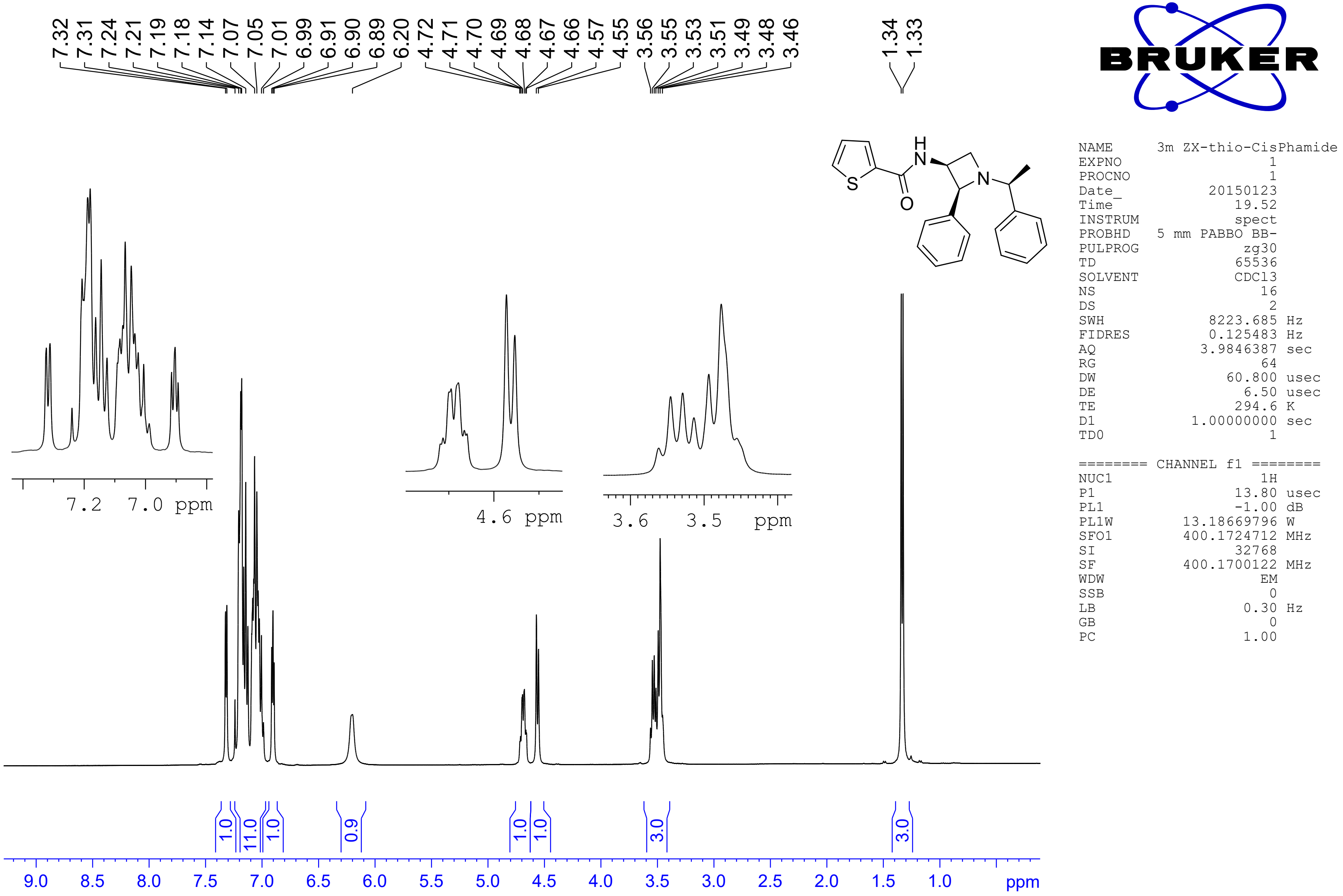
7.32 7.31 7.24 7.21 7.19 7.18 7.14 7.07 7.05 7.01 6.99 6.91 6.90 6.89 6.20 4.72 4.71 4.70 4.69 4.68 4.67 4.66 4.57 4.55 3.56 3.55 3.53 3.51 3.49 3.48 3.46 1.34 1.33
BRUKER
NAME 3m ZX-thio-CisPhamide
EXPNO 1
PROCNO 1
Date_ 20150123
Time 19.52
INSTRUM spect
PROBHD 5 mm PABBO BB-
PULPROG zg30
TD 65536
SOLVENT CDCl3
NS 16
DS 2
SWH 8223.685 Hz
FIDRES 0.125483 Hz
AQ 3.9846387 sec
RG 64
DW 60.800 usec
DE 6.50 usec
TE 294.6 K
D1 1.00000000 sec
TD0 1
======== CHANNEL f1 ========
NUC1 1H
P1 13.80 usec
PL1 -1.00 dB
PL1W 13.18669796 W
SFO1 400.1724712 MHz
SI 32768
SF 400.1700122 MHz
WDW EM
SSB 0
LB 0.30 Hz
GB 0
PC 1.00
7.2 7.0 ppm
4.6 ppm
3.6 3.5 ppm
1.0 11.0 1.0 0.9 1.0 1.0 3.0 3.0
9.0 8.5 8.0 7.5 7.0 6.5 6.0 5.5 5.0 4.5 4.0 3.5 3.0 2.5 2.0 1.5 1.0 ppm

3m ^{1}H NMR

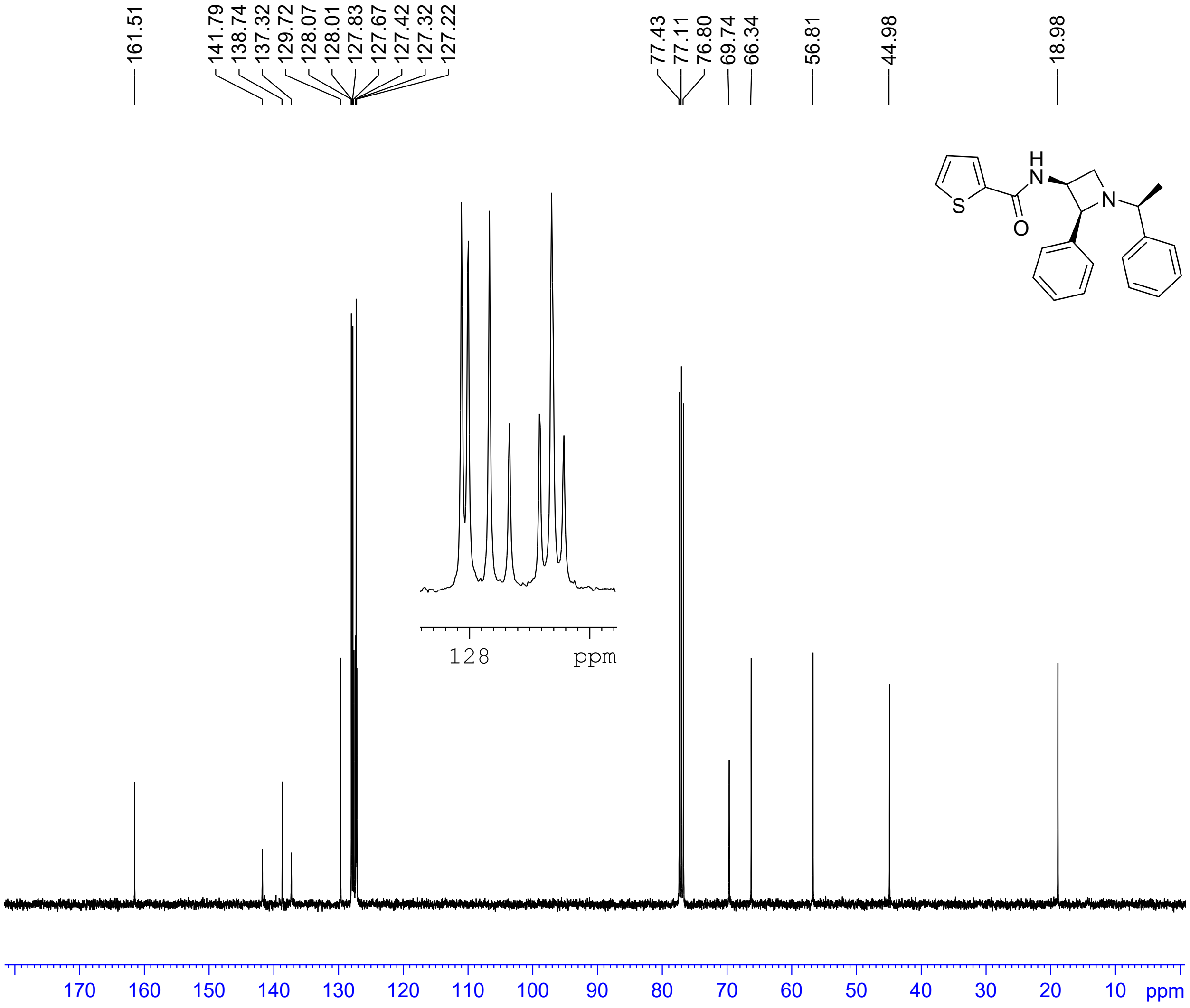


```
NAME       3m ZX-thio-CisPhamide-C13
EXPNO                      1
PROCNO                     1
Date_               20150123
Time                   20.58
INSTRUM                spect
PROBHD      5 mm PABBO BB-
PULPROG               zgpg30
TD                     65536
SOLVENT                CDCl3
NS                       200
DS                         4
SWH                24038.461 Hz
FIDRES              0.366798 Hz
AQ                 1.3631988 sec
RG                       203
DW                    20.800 usec
DE                      6.50 usec
TE                     295.1 K
D1                2.00000000 sec
D11               0.03000000 sec
TD0                        1

======== CHANNEL f1 ========
NUC1                     13C
P1                      8.50 usec
PL1                    -2.00 dB
PL1W             57.32743073 W
SFO1             100.6328888 MHz

======== CHANNEL f2 ========
CPDPRG2              waltz16
NUC2                      1H
PCPD2                  80.00 usec
PL2                    -1.00 dB
PL12                   14.26 dB
PL13                   14.46 dB
PL2W             13.18669796 W
PL12W             0.39276794 W
PL13W             0.37509048 W
SFO2             400.1716007 MHz
SI                     32768
SF               100.6228270 MHz
WDW                       EM
SSB                        0
LB                      1.00 Hz
GB                         0
PC                      1.40
```

3m ^{13}C NMR

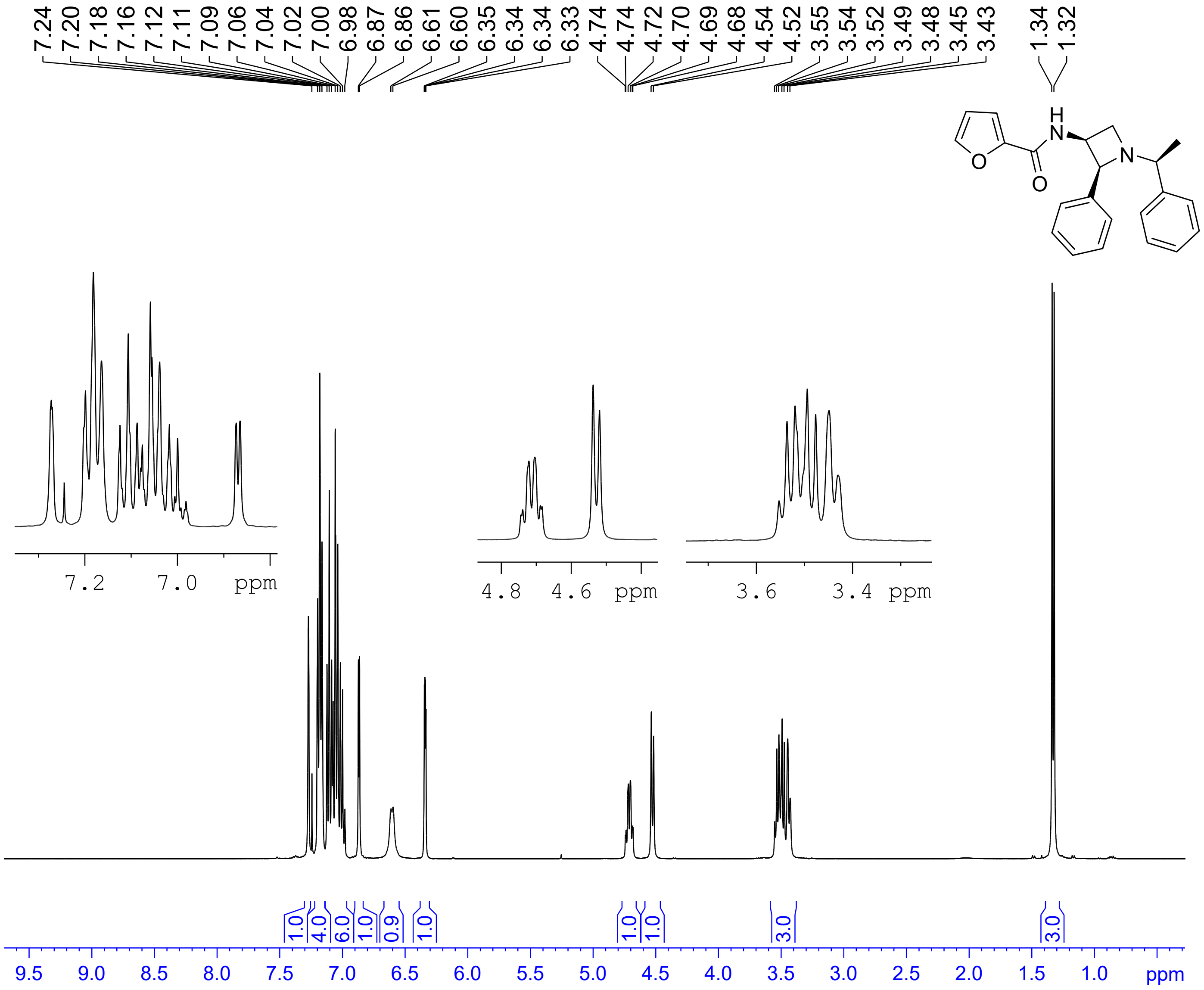


```
NAME       3n ZX-furanCisPhamide
EXPNO                  1
PROCNO                 1
Date_           20141122
Time               15.31
INSTRUM            spect
PROBHD   5 mm PABBO BB-
PULPROG             zg30
TD                 65536
SOLVENT            CDCl3
NS                    16
DS                     2
SWH             8223.685 Hz
FIDRES          0.125483 Hz
AQ             3.9846387 sec
RG                  80.6
DW                60.800 usec
DE                  6.50 usec
TE                 298.0 K
D1            1.00000000 sec
TD0                    1

======== CHANNEL f1 ========
NUC1                  1H
P1                 13.80 usec
PL1                -1.00 dB
PL1W         13.18669796 W
SFO1         400.1724712 MHz
SI                 32768
SF           400.1700100 MHz
WDW                   EM
SSB                    0
LB                  0.30 Hz
GB                     0
PC                  1.00
```

3n ^{1}H NMR

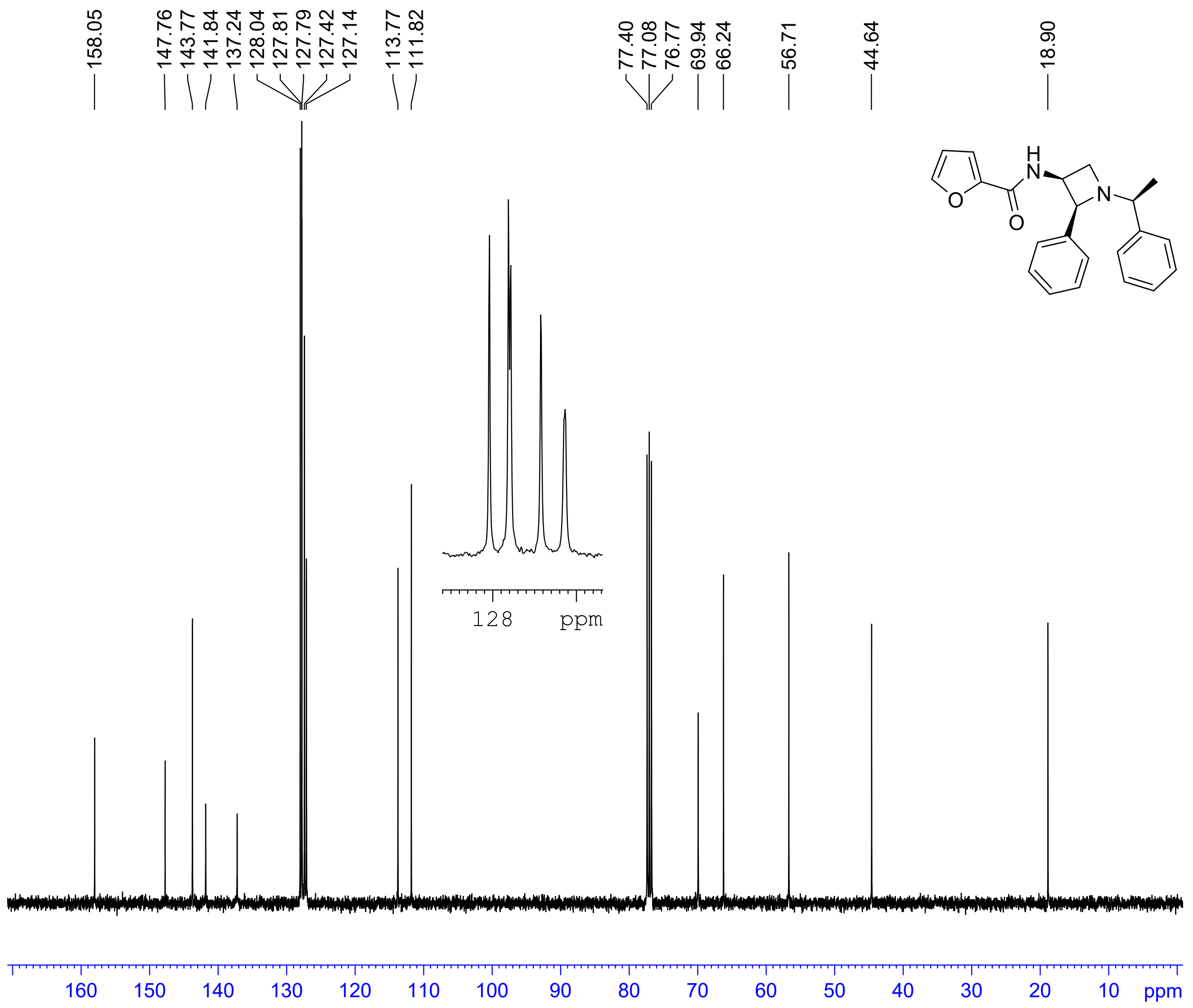

3n ^{13}C NMR

```
NAME      3n ZX-furanCisPhamide-C13
EXPNO                     1
PROCNO                    1
Date_              20141122
Time                  15.46
INSTRUM               spect
PROBHD     5 mm PABBO BB-
PULPROG             zgpg30
TD                    65536
SOLVENT               CDCl3
NS                      173
DS                        4
SWH               24038.461 Hz
FIDRES             0.366798 Hz
AQ                1.3631988 sec
RG                      203
DW                   20.800 usec
DE                     6.50 usec
TE                    298.3 K
D1               2.00000000 sec
D11              0.03000000 sec
TD0                       1

======== CHANNEL f1 ========
NUC1                    13C
P1                     8.50 usec
PL1                   -2.00 dB
PL1W            57.32743073 W
SFO1            100.6328888 MHz

======== CHANNEL f2 ========
CPDPRG2             waltz16
NUC2                     1H
PCPD2                 80.00 usec
PL2                   -1.00 dB
PL12                  14.26 dB
PL13                  14.46 dB
PL2W            13.18669796 W
PL12W            0.39276794 W
PL13W            0.37509048 W
SFO2            400.1716007 MHz
SI                    32768
SF              100.6228270 MHz
WDW                      EM
SSB                       0
LB                     1.00 Hz
GB                        0
PC                     1.40
```

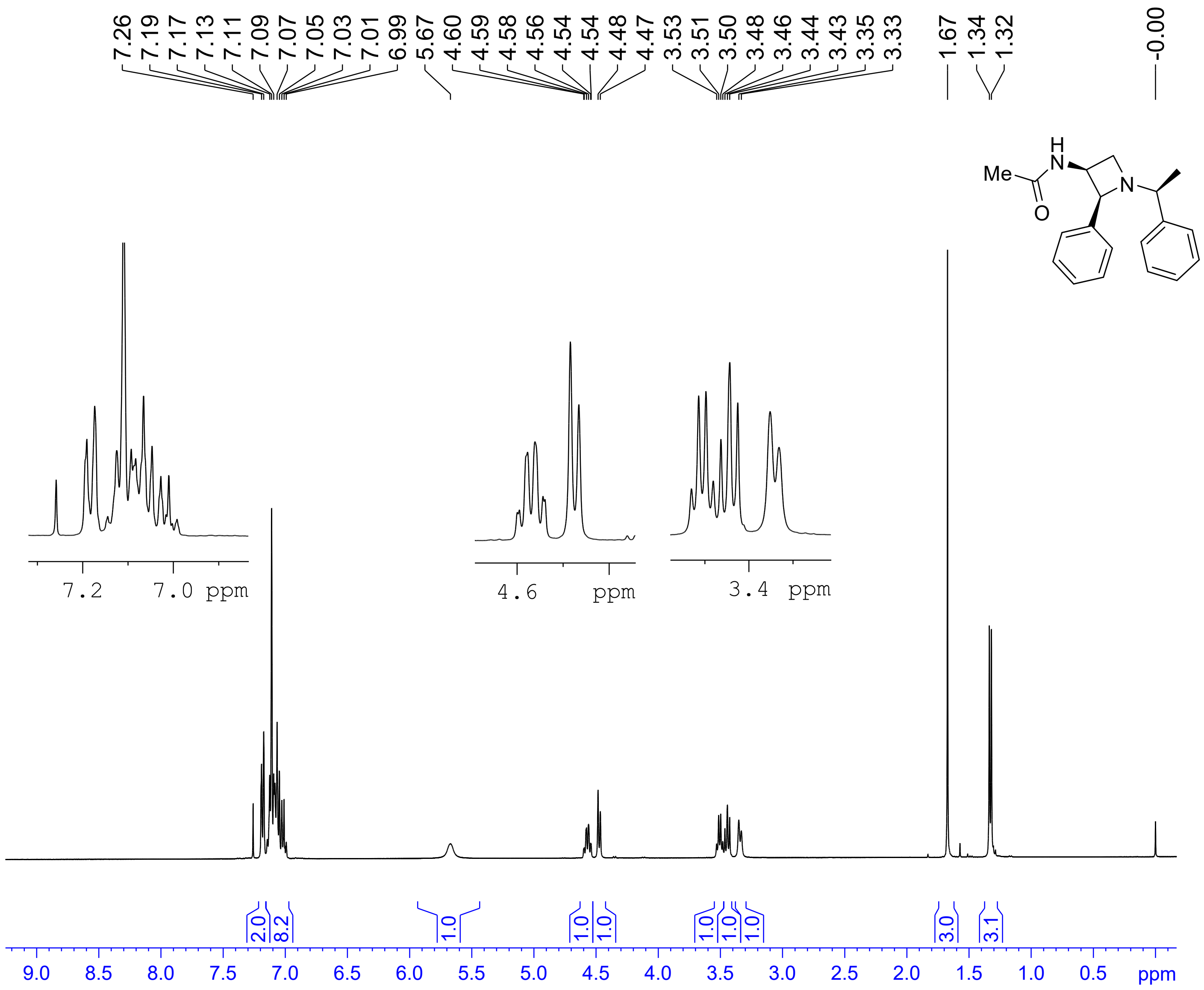

NAME	3o ZX-Me-CisPhamide
EXPNO	1
PROCNO	1
Date_	20150130
Time	11.30
INSTRUM	spect
PROBHD	5 mm PABBO BB-
PULPROG	zg30
TD	65536
SOLVENT	CDCl3
NS	16
DS	2
SWH	8223.685 Hz
FIDRES	0.125483 Hz
AQ	3.9846387 sec
RG	101
DW	60.800 usec
DE	6.50 usec
TE	292.9 K
D1	1.00000000 sec
TD0	1
======== CHANNEL f1 ========	
NUC1	1H
P1	13.80 usec
PL1	-1.00 dB
PL1W	13.18669796 W
SFO1	400.1724712 MHz
SI	32768
SF	400.1700043 MHz
WDW	EM
SSB	0
LB	0.30 Hz
GB	0
PC	1.00

3o ^{1}H NMR

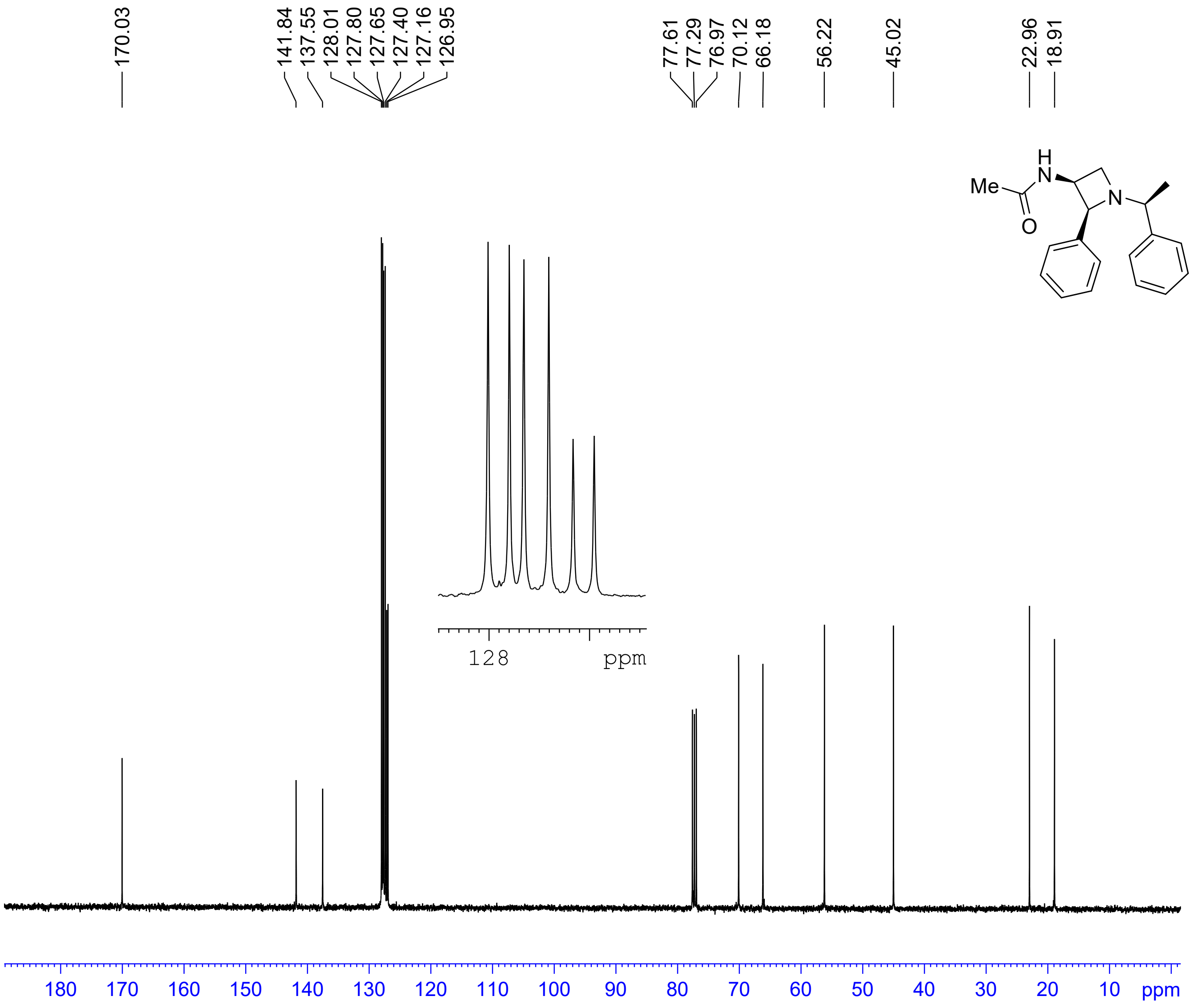


```
NAME       3o ZX-Me-CisPhamide-C13
EXPNO                    1
PROCNO                   1
Date_             20150202
Time                 22.16
INSTRUM              spect
PROBHD     5 mm PABBO BB-
PULPROG             zgpg30
TD                   65536
SOLVENT              CDCl3
NS                      62
DS                       4
SWH              24038.461 Hz
FIDRES            0.366798 Hz
AQ               1.3631988 sec
RG                     203
DW                  20.800 usec
DE                    6.50 usec
TE                   291.9 K
D1              2.00000000 sec
D11             0.03000000 sec
TD0                      1

======== CHANNEL f1 ========
NUC1                   13C
P1                    8.50 usec
PL1                  -2.00 dB
PL1W           57.32743073 W
SFO1           100.6328888 MHz

======== CHANNEL f2 ========
CPDPRG2            waltz16
NUC2                    1H
PCPD2                80.00 usec
PL2                  -1.00 dB
PL12                 14.26 dB
PL13                 14.46 dB
PL2W           13.18669796 W
PL12W           0.39276794 W
PL13W           0.37509048 W
SFO2           400.1716007 MHz
SI                   32768
SF             100.6228270 MHz
WDW                     EM
SSB                      0
LB                    1.00 Hz
GB                       0
PC                    1.40
```

3o ^{13}C NMR

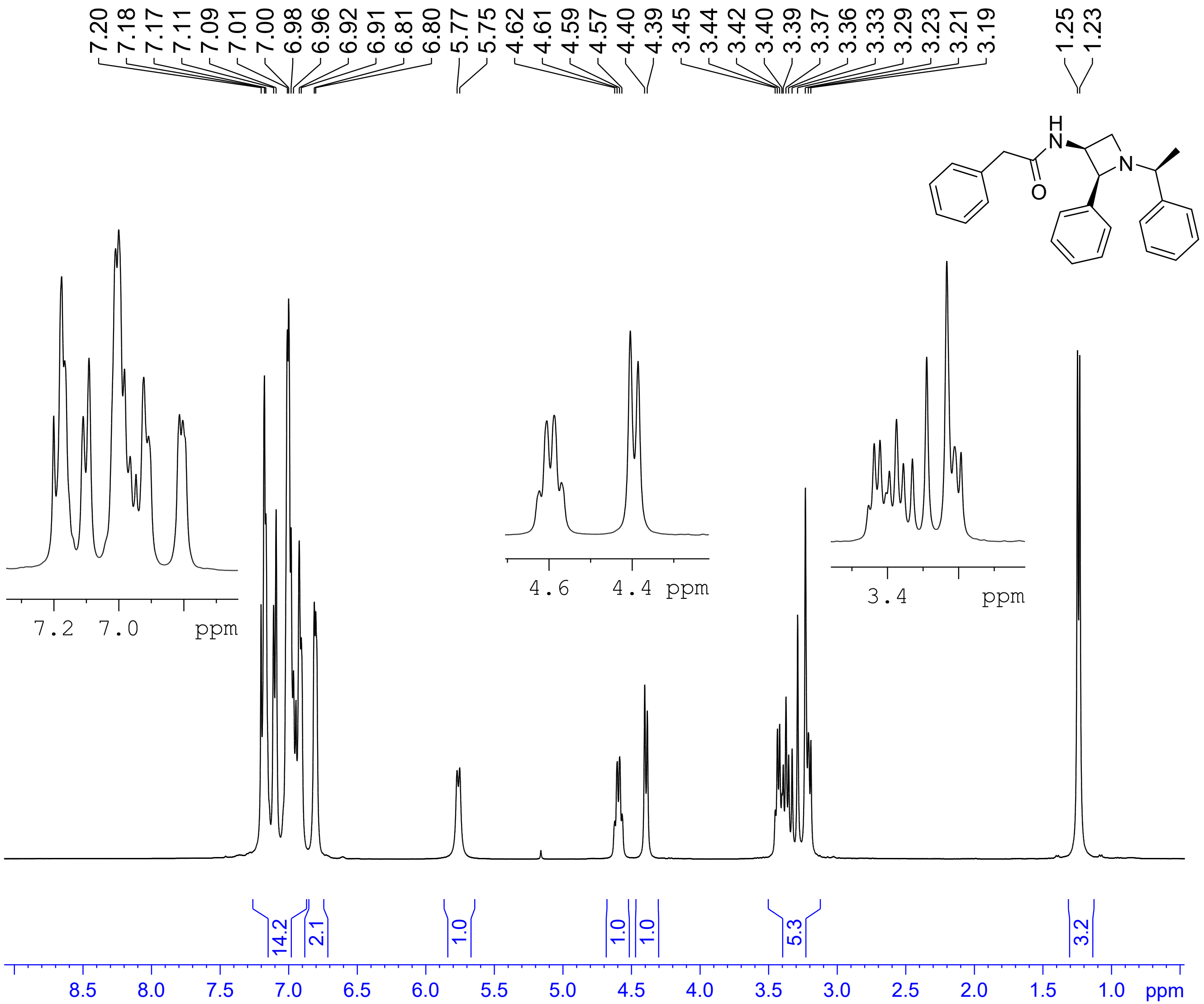

NAME	3p ZX-Bn-CisPhamide
EXPNO	1
PROCNO	1
Date_	20141112
Time	21.30
INSTRUM	spect
PROBHD	5 mm PABBO BB-
PULPROG	zg30
TD	65536
SOLVENT	CDCl3
NS	16
DS	2
SWH	8223.685 Hz
FIDRES	0.125483 Hz
AQ	3.9846387 sec
RG	20.2
DW	60.800 usec
DE	6.50 usec
TE	292.1 K
D1	1.00000000 sec
TD0	1

======== CHANNEL f1 ========

NUC1	1H
P1	13.80 usec
PL1	-1.00 dB
PL1W	13.18669796 W
SFO1	400.1724712 MHz
SI	32768
SF	400.1700279 MHz
WDW	EM
SSB	0
LB	0.30 Hz
GB	0
PC	1.00

3p ^{1}H NMR

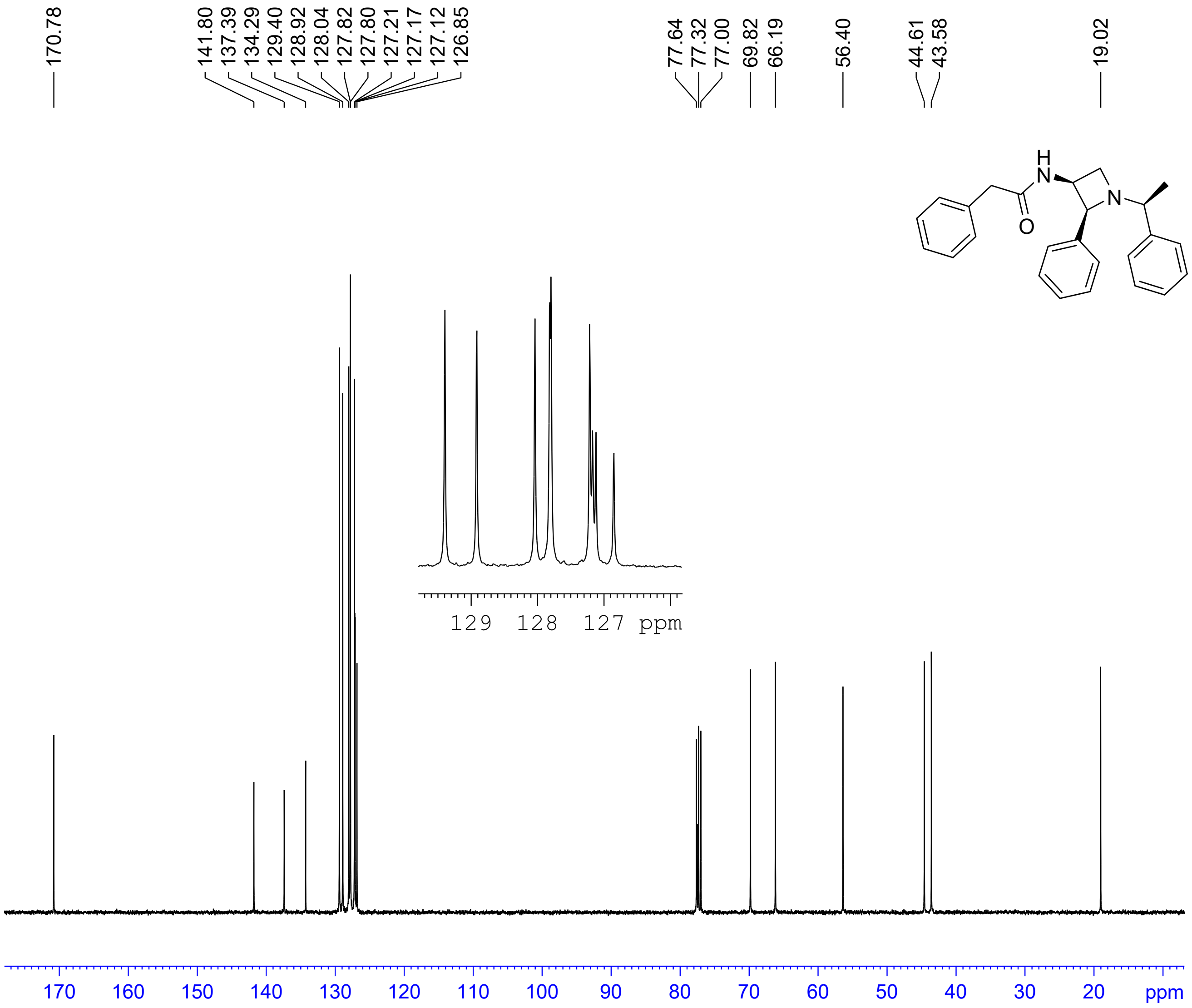

3p ^{13}C NMR

```
NAME       3p ZX-Bn-CisPhamide-C13
EXPNO                    1
PROCNO                   1
Date_             20141112
Time                 21.33
INSTRUM              spect
PROBHD     5 mm PABBO BB-
PULPROG            zgpg30
TD                   65536
SOLVENT              CDCl3
NS                     168
DS                       4
SWH              24038.461 Hz
FIDRES            0.366798 Hz
AQ               1.3631988 sec
RG                     203
DW                  20.800 usec
DE                    6.50 usec
TE                   292.6 K
D1              2.00000000 sec
D11             0.03000000 sec
TD0                      1

======== CHANNEL f1 ========
NUC1                   13C
P1                    8.50 usec
PL1                  -2.00 dB
PL1W           57.32743073 W
SFO1           100.6328888 MHz

======== CHANNEL f2 ========
CPDPRG2            waltz16
NUC2                    1H
PCPD2                80.00 usec
PL2                  -1.00 dB
PL12                 14.26 dB
PL13                 14.46 dB
PL2W           13.18669796 W
PL12W           0.39276794 W
PL13W           0.37509048 W
SFO2           400.1716007 MHz
SI                   32768
SF             100.6228270 MHz
WDW                     EM
SSB                      0
LB                    1.00 Hz
GB                       0
PC                    1.40
```

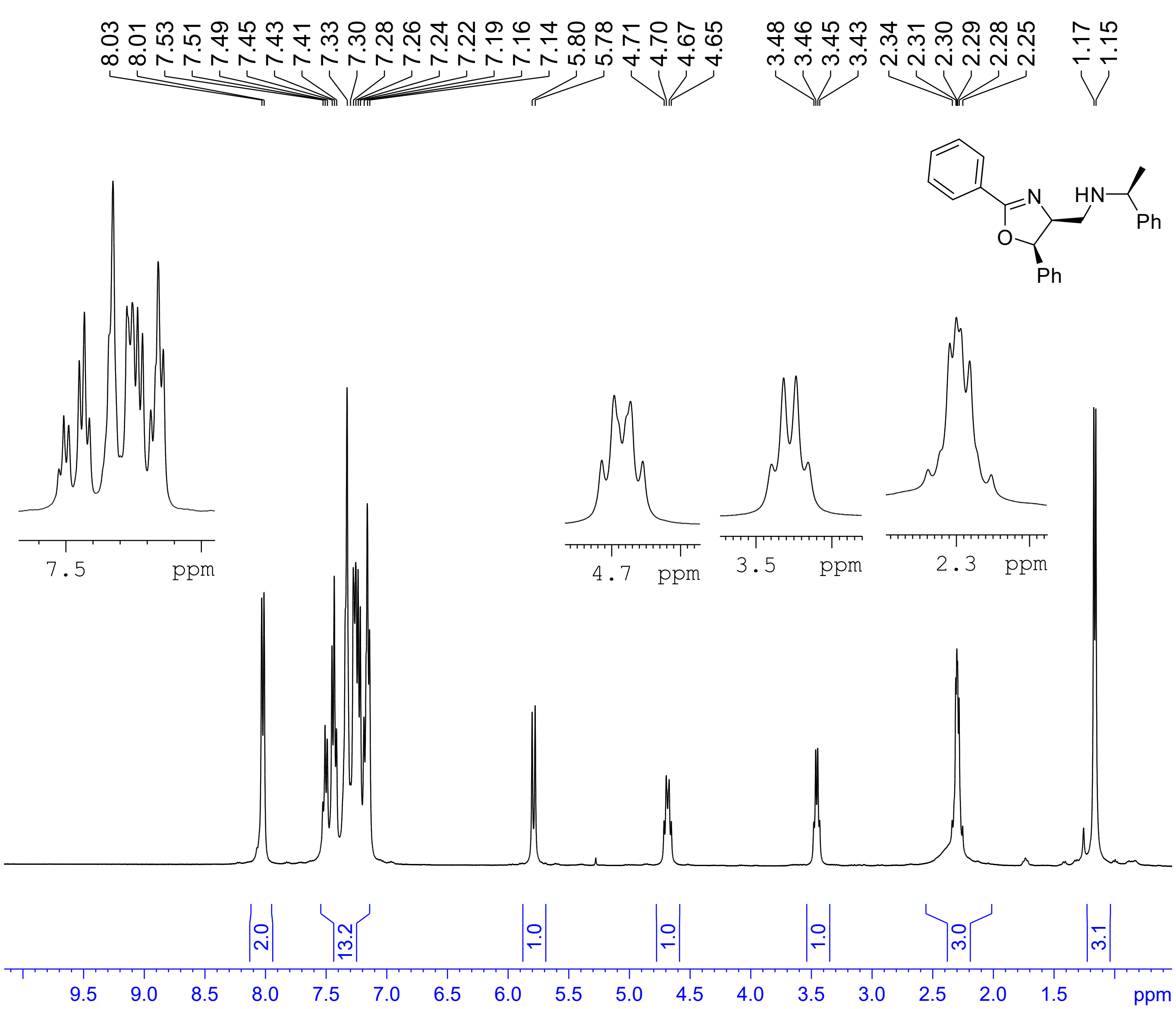

4a ^{1}H NMR

```
NAME       4a ZX-Ph-oxazoline
EXPNO                   1
PROCNO                  1
Date_            20141205
Time                11.10
INSTRUM             spect
PROBHD     5 mm PABBO BB-
PULPROG              zg30
TD                  65536
SOLVENT             CDCl3
NS                     16
DS                      2
SWH              8223.685 Hz
FIDRES           0.125483 Hz
AQ              3.9846387 sec
RG                   90.5
DW                 60.800 usec
DE                   6.50 usec
TE                  292.2 K
D1             1.00000000 sec
TD0                     1

======== CHANNEL f1 ========
NUC1                   1H
P1                  13.80 usec
PL1                 -1.00 dB
PL1W          13.18669796 W
SFO1          400.1724712 MHz
SI                  32768
SF            400.1700089 MHz
WDW                    EM
SSB                     0
LB                   0.30 Hz
GB                      0
PC                   1.00
```

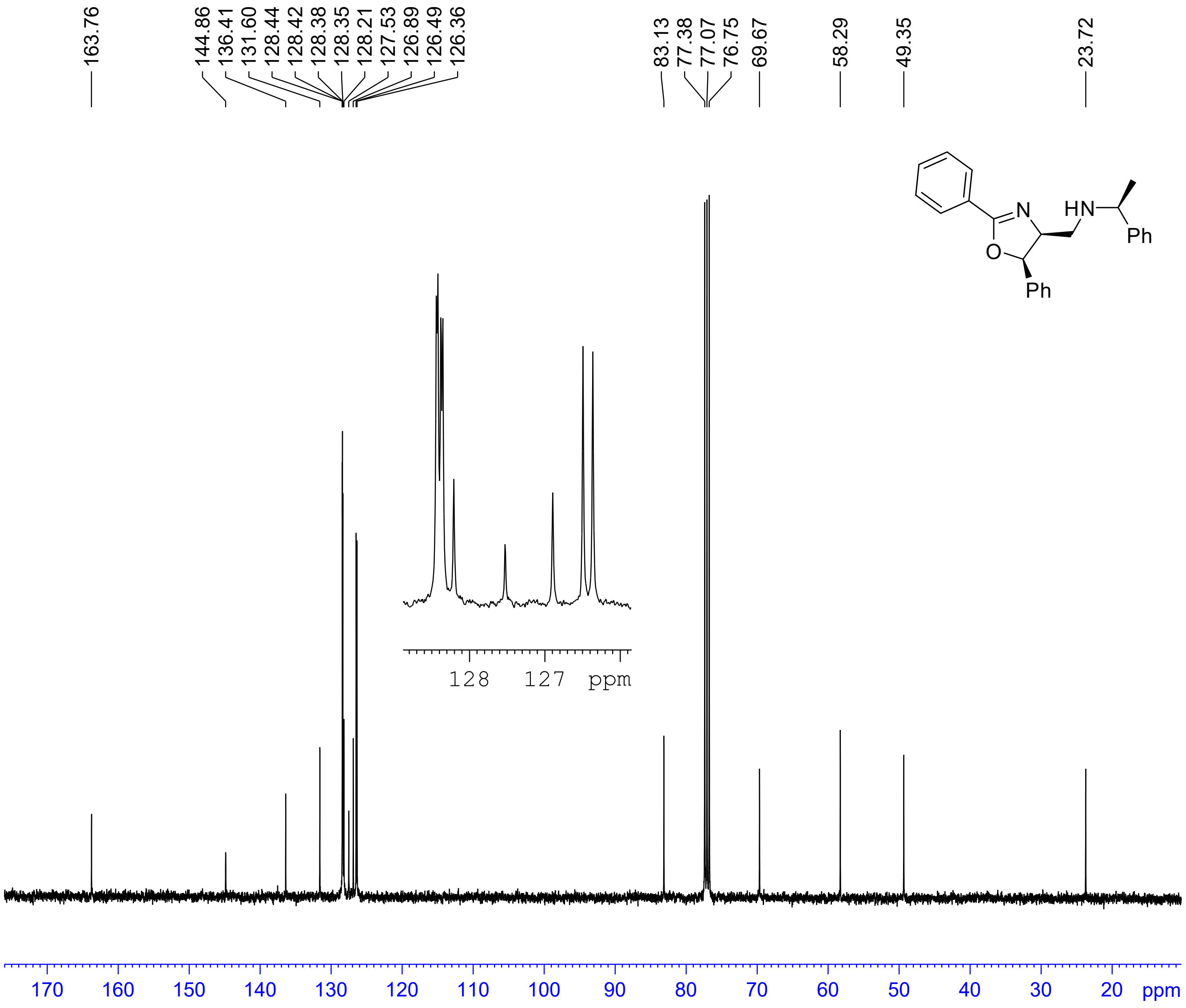

4a ^{13}C NMR

```
NAME      4a ZX-Ph-oxazoline-C13
EXPNO                     1
PROCNO                    1
Date_              20141205
Time                  11.14
INSTRUM               spect
PROBHD     5 mm PABBO BB-
PULPROG              zgpg30
TD                    65536
SOLVENT               CDCl3
NS                      216
DS                        4
SWH              24038.461 Hz
FIDRES            0.366798 Hz
AQ               1.3631988 sec
RG                      203
DW                   20.800 usec
DE                     6.50 usec
TE                    292.8 K
D1               2.00000000 sec
D11              0.03000000 sec
TD0                       1

======== CHANNEL f1 ========
NUC1                    13C
P1                     8.50 usec
PL1                   -2.00 dB
PL1W            57.32743073 W
SFO1            100.6328888 MHz

======== CHANNEL f2 ========
CPDPRG2             waltz16
NUC2                     1H
PCPD2                 80.00 usec
PL2                   -1.00 dB
PL12                  14.26 dB
PL13                  14.46 dB
PL2W            13.18669796 W
PL12W            0.39276794 W
PL13W            0.37509048 W
SFO2            400.1716007 MHz
SI                    32768
SF              100.6228270 MHz
WDW                      EM
SSB                       0
LB                     1.00 Hz
GB                        0
PC                     1.40
```

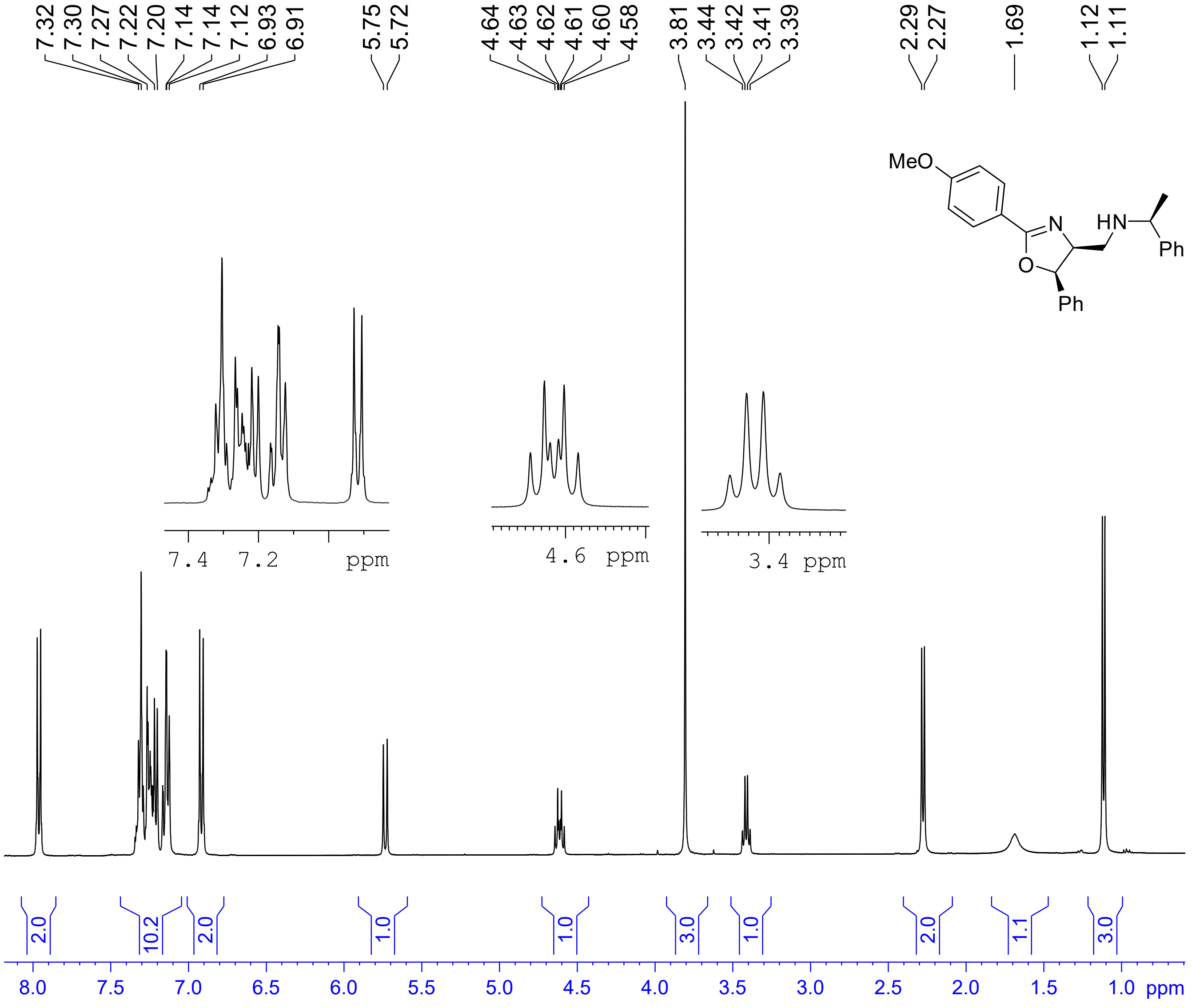

BRUKER

```
NAME       4b ZX-pOME-oxazoline
EXPNO                     1
PROCNO                    1
Date_              20150402
Time                  10.37
INSTRUM               spect
PROBHD     5 mm PABBO BB-
PULPROG                zg30
TD                    65536
SOLVENT               CDCl3
NS                       16
DS                        2
SWH                8223.685 Hz
FIDRES             0.125483 Hz
AQ                3.9846387 sec
RG                     28.5
DW                   60.800 usec
DE                     6.50 usec
TE                    293.3 K
D1               1.00000000 sec
TD0                       1

======== CHANNEL f1 ========
NUC1                     1H
P1                    13.80 usec
PL1                   -1.00 dB
PL1W            13.18669796 W
SFO1            400.1724712 MHz
SI                    32768
SF              400.1700168 MHz
WDW                      EM
SSB                       0
LB                     0.30 Hz
GB                        0
PC                     1.00
```

4b ^{1}H NMR

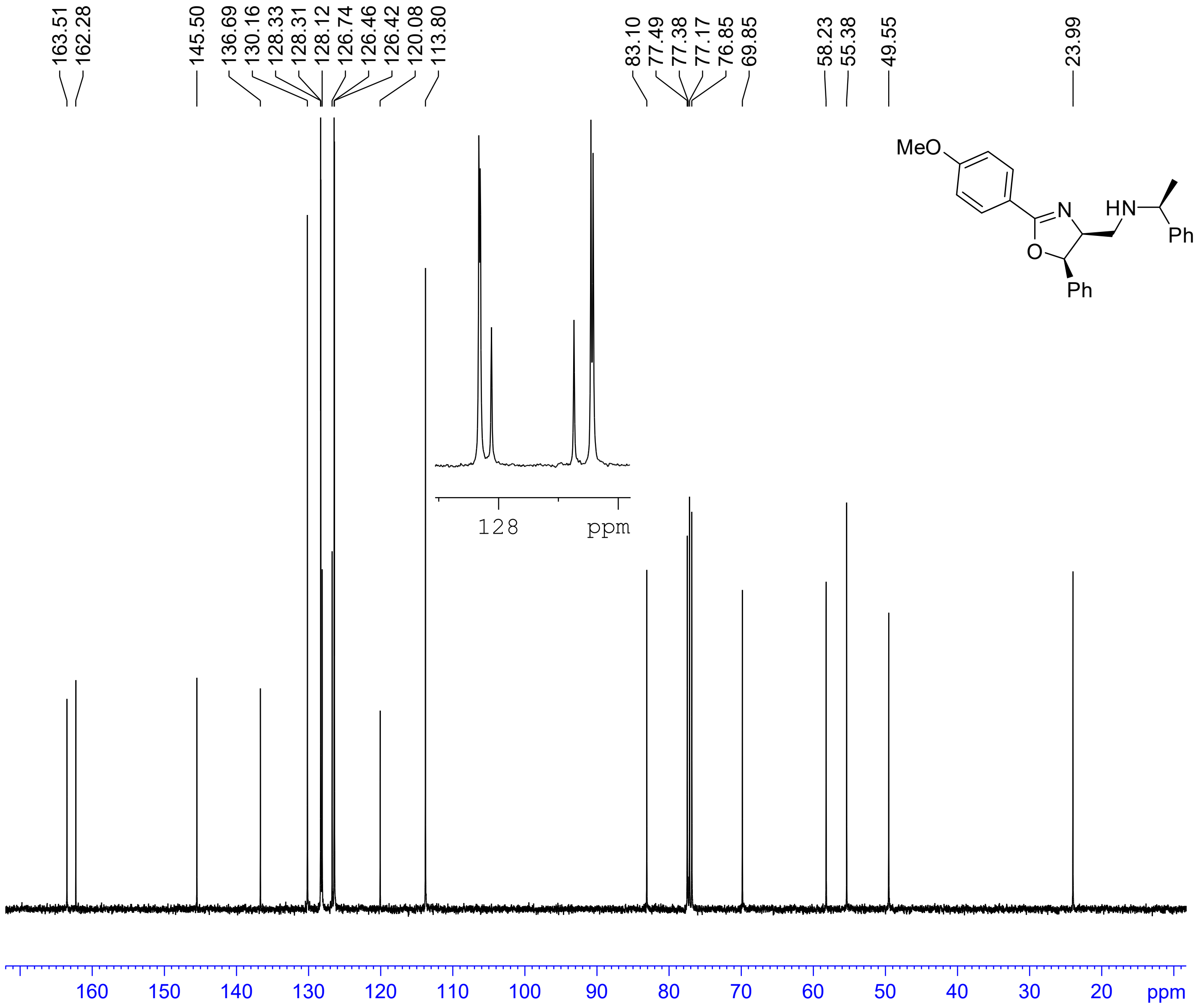


```
NAME       4b ZX-pOME-oxazoline-C13
EXPNO                    1
PROCNO                   1
Date_             20150402
Time                 14.38
INSTRUM              spect
PROBHD     5 mm PABBO BB-
PULPROG            zgpg30
TD                   65536
SOLVENT              CDCl3
NS                     150
DS                       4
SWH              24038.461 Hz
FIDRES            0.366798 Hz
AQ               1.3631988 sec
RG                     203
DW                  20.800 usec
DE                    6.50 usec
TE                   294.8 K
D1              2.00000000 sec
D11             0.03000000 sec
TD0                      1

======== CHANNEL f1 ========
NUC1                   13C
P1                    8.50 usec
PL1                  -2.00 dB
PL1W           57.32743073 W
SFO1           100.6328888 MHz

======== CHANNEL f2 ========
CPDPRG2            waltz16
NUC2                    1H
PCPD2                80.00 usec
PL2                  -1.00 dB
PL12                 14.26 dB
PL13                 14.46 dB
PL2W           13.18669796 W
PL12W           0.39276794 W
PL13W           0.37509048 W
SFO2           400.1716007 MHz
SI                   32768
SF             100.6228270 MHz
WDW                     EM
SSB                      0
LB                    1.00 Hz
GB                       0
PC                    1.40
```

4b ^{13}C NMR

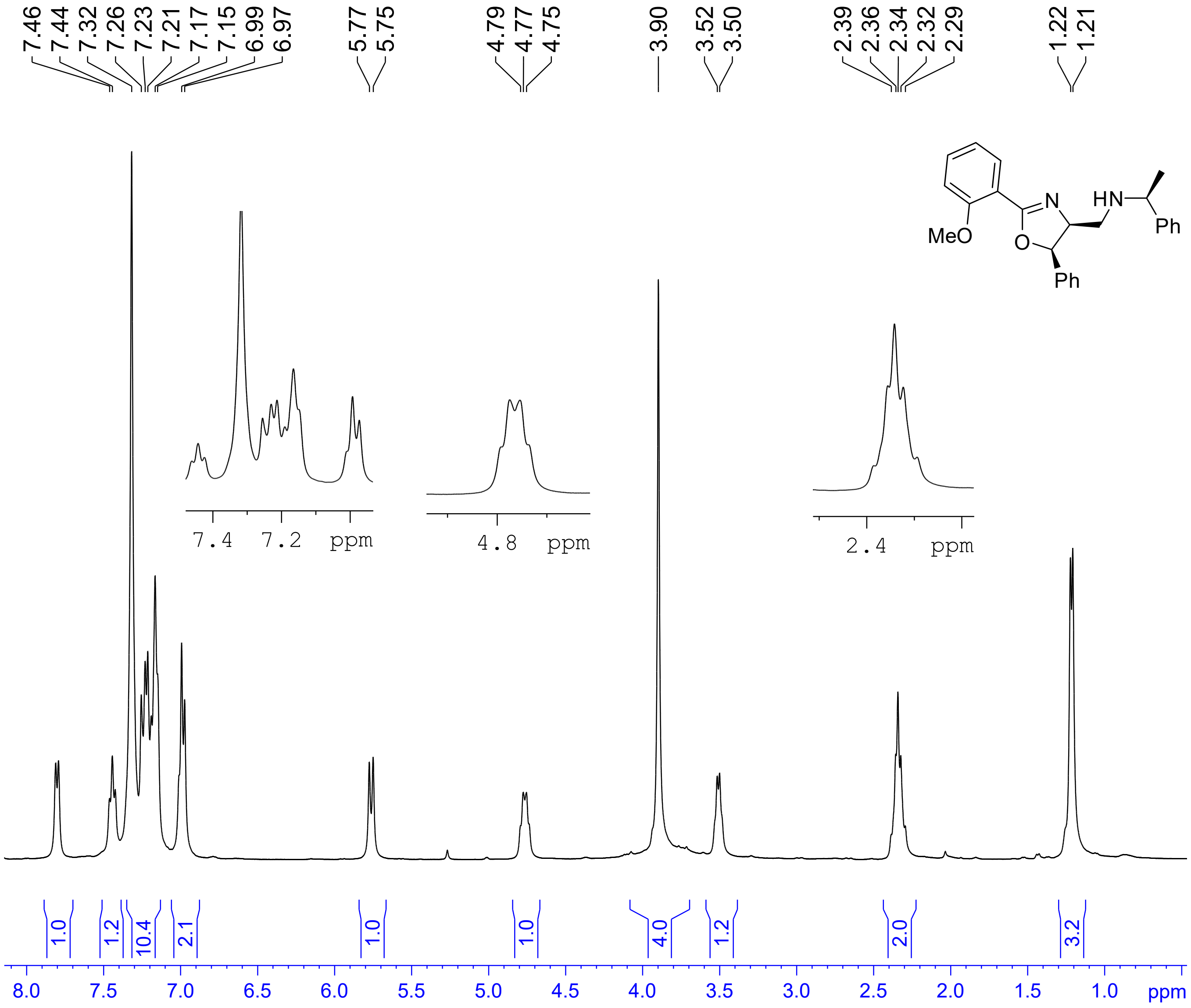

4c ^{1}H NMR

NAME	4c ZX-oOMe-oxazoline
EXPNO	1
PROCNO	1
Date_	20141112
Time	15.46
INSTRUM	spect
PROBHD	5 mm PABBO BB-
PULPROG	zg30
TD	65536
SOLVENT	CDCl3
NS	16
DS	2
SWH	8223.685 Hz
FIDRES	0.125483 Hz
AQ	3.9846387 sec
RG	64
DW	60.800 usec
DE	6.50 usec
TE	293.5 K
D1	1.00000000 sec
TD0	1

======== CHANNEL f1 ========

NUC1	1H
P1	13.80 usec
PL1	-1.00 dB
PL1W	13.18669796 W
SFO1	400.1724712 MHz
SI	32768
SF	400.1700067 MHz
WDW	EM
SSB	0
LB	0.30 Hz
GB	0
PC	1.00

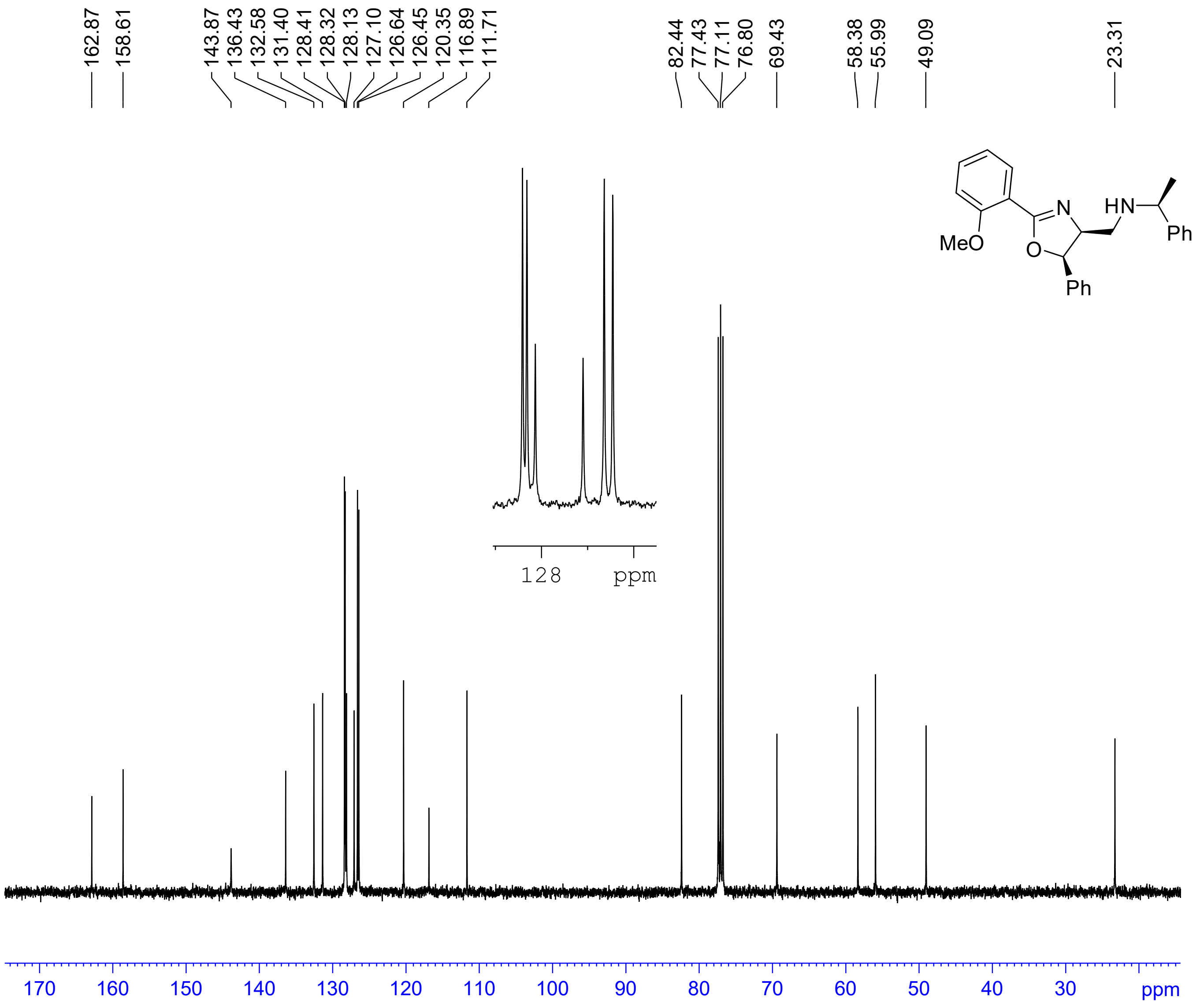

4c ^{13}C NMR

```
NAME         4c ZX-oOMe-oxazoline-C13
EXPNO                       1
PROCNO                      1
Date_                20141112
Time                    16.32
INSTRUM                 spect
PROBHD       5 mm PABBO BB-
PULPROG               zgpg30
TD                      65536
SOLVENT                 CDCl3
NS                        204
DS                          4
SWH                 24038.461 Hz
FIDRES               0.366798 Hz
AQ                  1.3631988 sec
RG                        203
DW                     20.800 usec
DE                       6.50 usec
TE                      293.8 K
D1                 2.00000000 sec
D11                0.03000000 sec
TD0                         1

======== CHANNEL f1 ========
NUC1                      13C
P1                       8.50 usec
PL1                     -2.00 dB
PL1W              57.32743073 W
SFO1              100.6328888 MHz

======== CHANNEL f2 ========
CPDPRG2               waltz16
NUC2                       1H
PCPD2                   80.00 usec
PL2                     -1.00 dB
PL12                    14.26 dB
PL13                    14.46 dB
PL2W              13.18669796 W
PL12W              0.39276794 W
PL13W              0.37509048 W
SFO2              400.1716007 MHz
SI                      32768
SF                100.6228270 MHz
WDW                        EM
SSB                         0
LB                       1.00 Hz
GB                          0
PC                       1.40
```

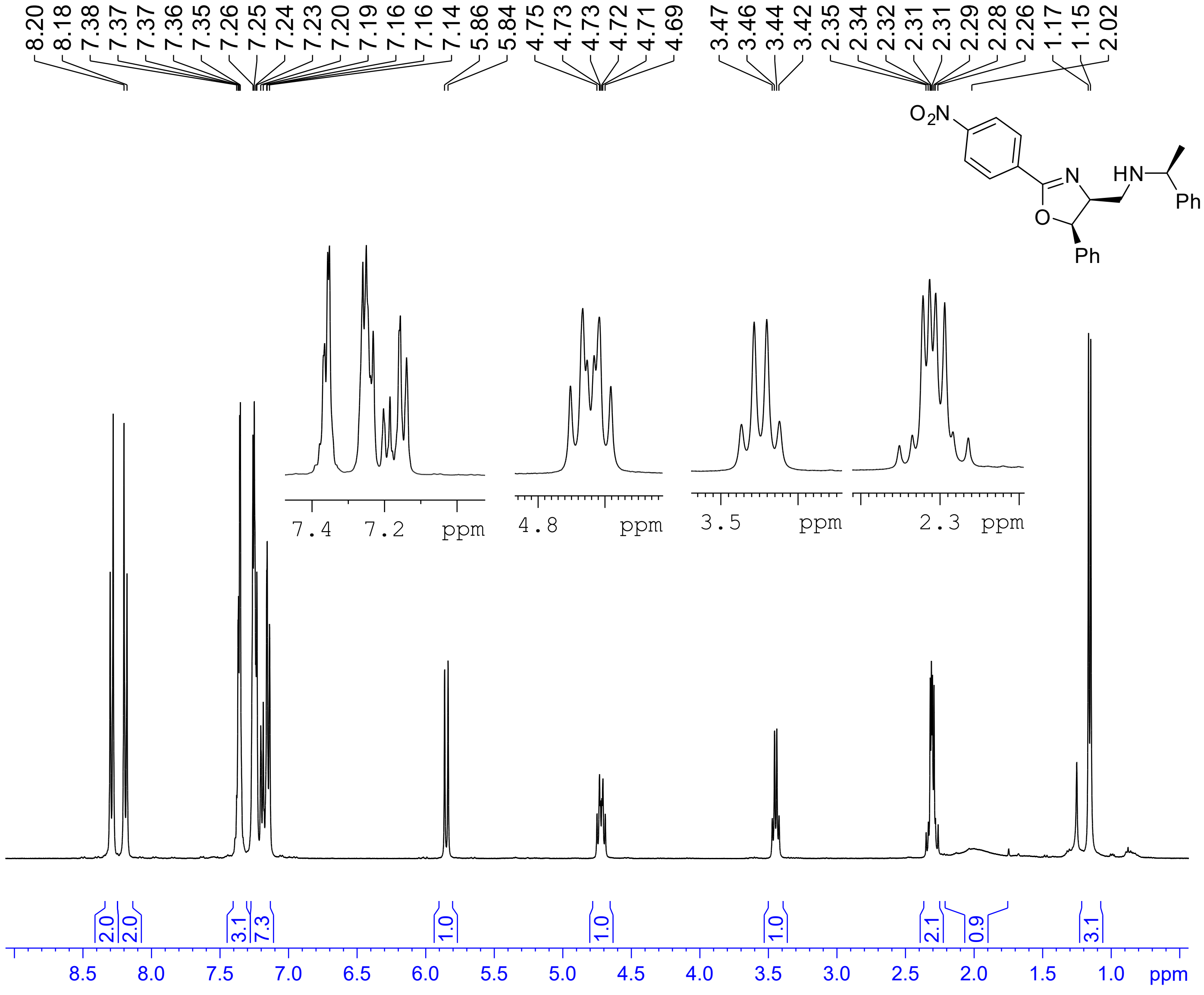


```
NAME       4d ZX-pNO2-oxazoline-1
EXPNO                     1
PROCNO                    1
Date_              20141220
Time                   8.46
INSTRUM               spect
PROBHD       5 mm PABBO BB-
PULPROG                zg30
TD                    65536
SOLVENT               CDCl3
NS                       16
DS                        2
SWH                8223.685 Hz
FIDRES             0.125483 Hz
AQ                3.9846387 sec
RG                      144
DW                   60.800 usec
DE                     6.50 usec
TE                    292.2 K
D1               1.00000000 sec
TD0                       1

======== CHANNEL f1 ========
NUC1                     1H
P1                    13.80 usec
PL1                   -1.00 dB
PL1W            13.18669796 W
SFO1            400.1724712 MHz
SI                    32768
SF              400.1700042 MHz
WDW                      EM
SSB                       0
LB                     0.30 Hz
GB                        0
PC                     1.00
```

4d ¹H NMR

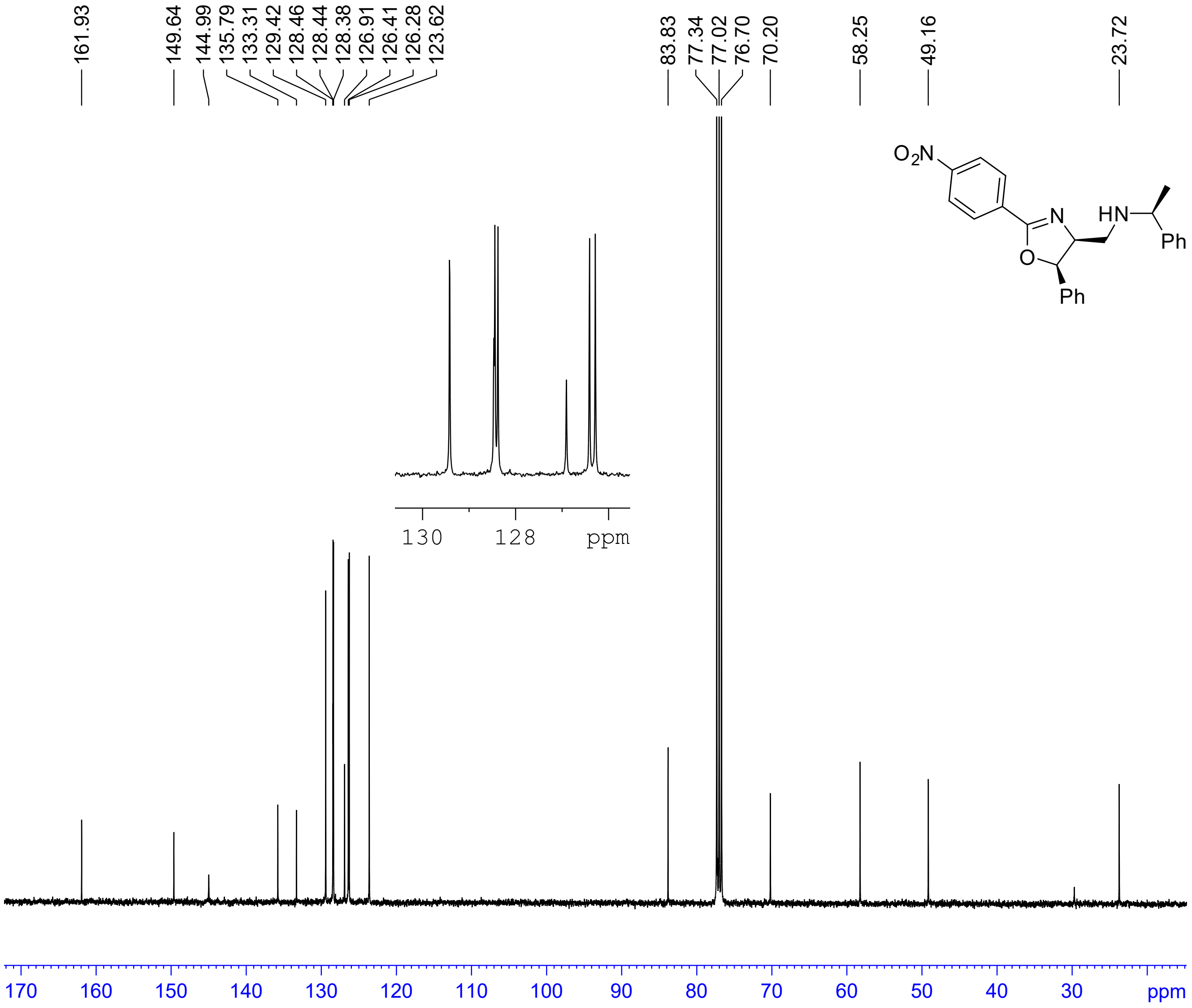

4d ^{13}C NMR

```
NAME      4d ZX-pNO2-oxazoline-C13-1
EXPNO                  1
PROCNO                 1
Date_           20141220
Time               13.08
INSTRUM            spect
PROBHD    5 mm PABBO BB-
PULPROG          zgpg30
TD                 65536
SOLVENT            CDCl3
NS                  1500
DS                     4
SWH           24038.461 Hz
FIDRES         0.366798 Hz
AQ            1.3631988 sec
RG                   203
DW                20.800 usec
DE                  6.50 usec
TE                 295.8 K
D1            2.00000000 sec
D11           0.03000000 sec
TD0                    1

======== CHANNEL f1 ========
NUC1                 13C
P1                  8.50 usec
PL1                -2.00 dB
PL1W         57.32743073 W
SFO1         100.6328888 MHz

======== CHANNEL f2 ========
CPDPRG2          waltz16
NUC2                  1H
PCPD2              80.00 usec
PL2                -1.00 dB
PL12               14.26 dB
PL13               14.46 dB
PL2W         13.18669796 W
PL12W         0.39276794 W
PL13W         0.37509048 W
SFO2         400.1716007 MHz
SI                 32768
SF           100.6228274 MHz
WDW                   EM
SSB                    0
LB                  1.00 Hz
GB                     0
PC                  1.40
```

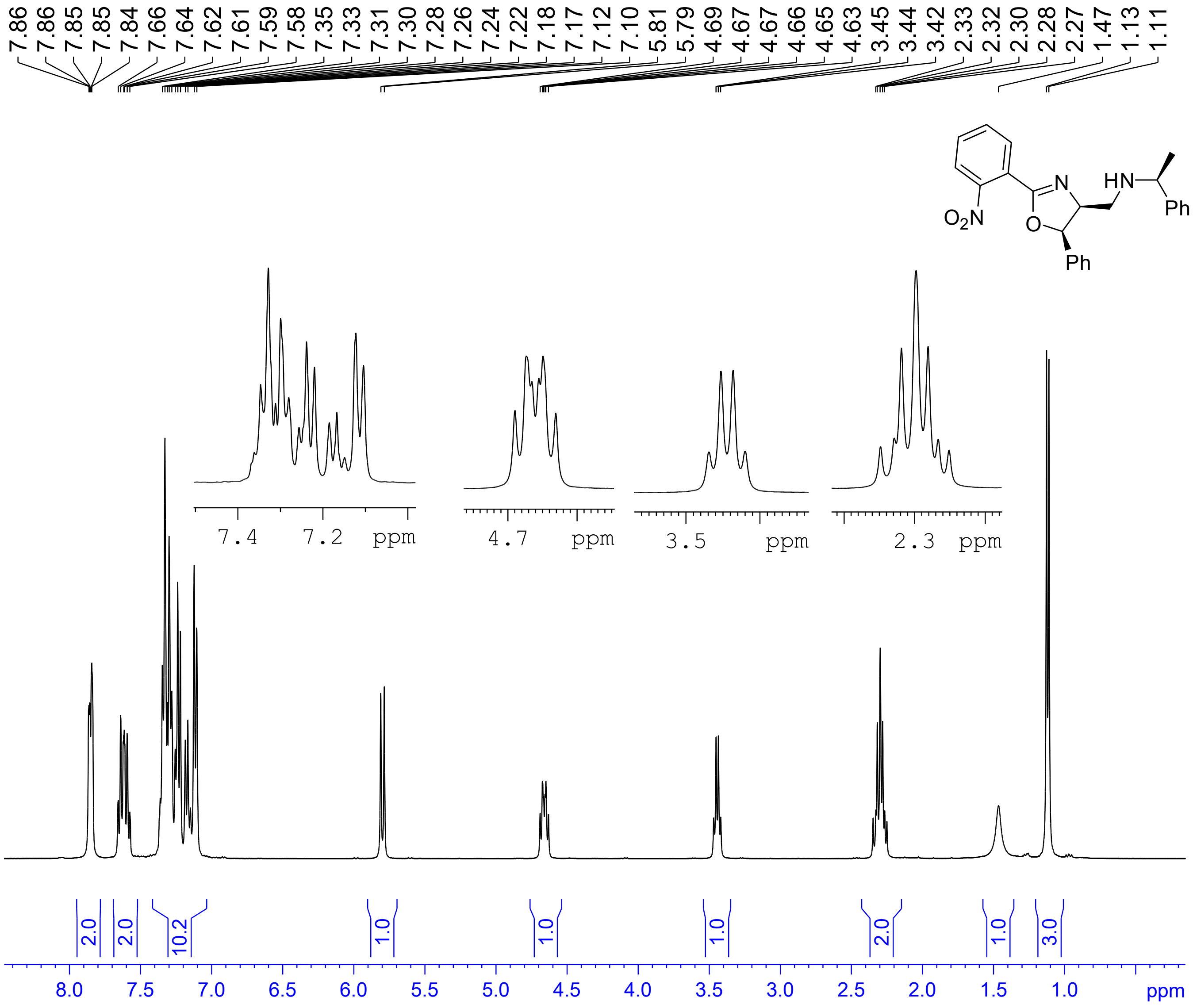

4e ¹H NMR

```
NAME       4e ZX-oNO2-oxazoline
EXPNO                     1
PROCNO                    1
Date_              20150127
Time                  11.44
INSTRUM               spect
PROBHD      5 mm PABBO BB-
PULPROG                zg30
TD                    65536
SOLVENT               CDCl3
NS                       16
DS                        2
SWH                8223.685 Hz
FIDRES             0.125483 Hz
AQ                3.9846387 sec
RG                       36
DW                   60.800 usec
DE                     6.50 usec
TE                    293.7 K
D1               1.00000000 sec
TD0                       1

======== CHANNEL f1 ========
NUC1                     1H
P1                    13.80 usec
PL1                   -1.00 dB
PL1W            13.18669796 W
SFO1            400.1724712 MHz
SI                    32768
SF              400.1700094 MHz
WDW                      EM
SSB                       0
LB                     0.30 Hz
GB                        0
PC                     1.00
```

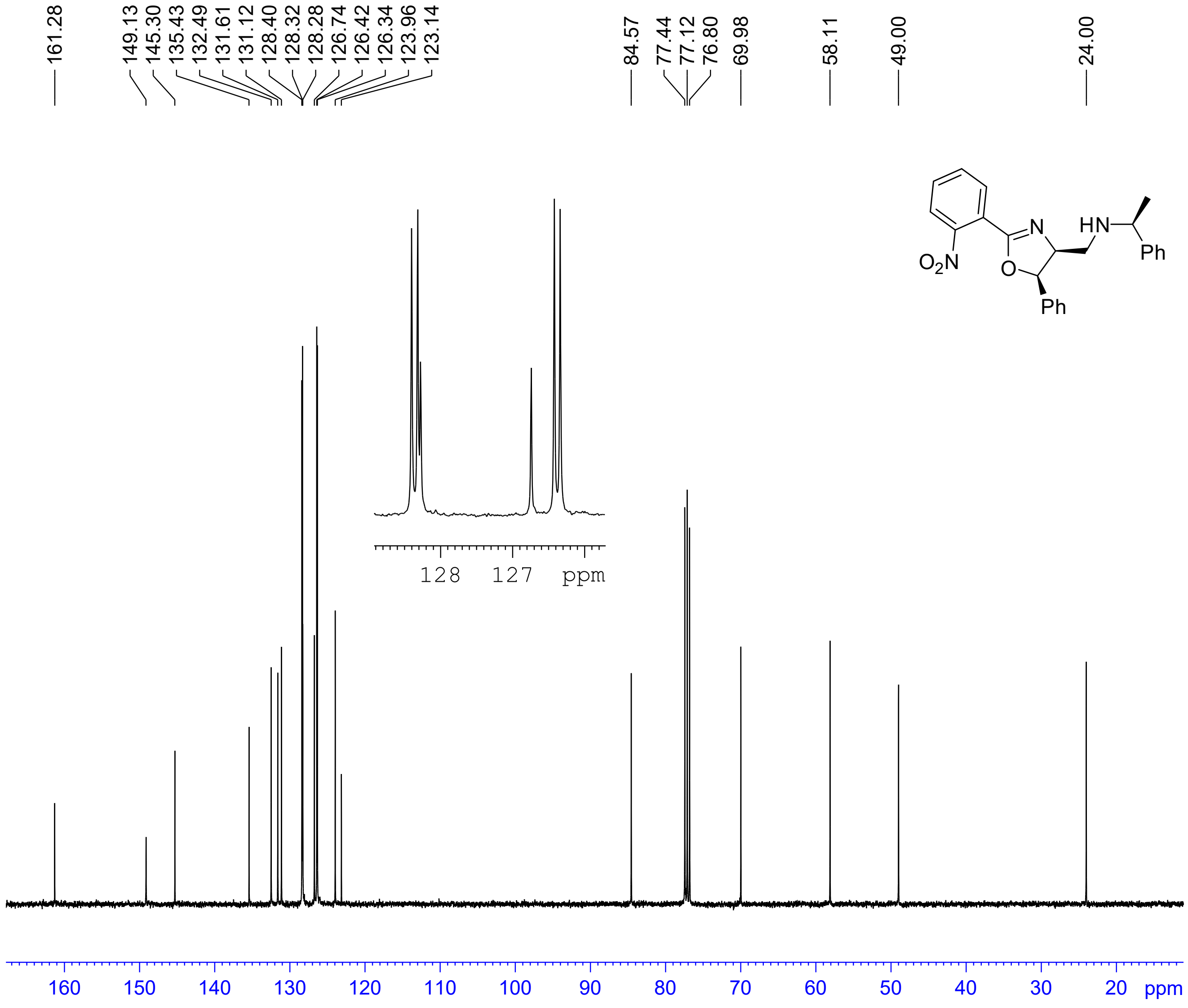

NAME	4e ZX-oNO2-oxazoline-C13
EXPNO	1
PROCNO	1
Date_	20150127
Time	18.57
INSTRUM	spect
PROBHD	5 mm PABBO BB-
PULPROG	zgpg30
TD	65536
SOLVENT	CDCl3
NS	250
DS	4
SWH	24038.461 Hz
FIDRES	0.366798 Hz
AQ	1.3631988 sec
RG	203
DW	20.800 usec
DE	6.50 usec
TE	295.1 K
D1	2.00000000 sec
D11	0.03000000 sec
TD0	1
======== CHANNEL f1 ========	
NUC1	13C
P1	8.50 usec
PL1	-2.00 dB
PL1W	57.32743073 W
SFO1	100.6328888 MHz
======== CHANNEL f2 ========	
CPDPRG2	waltz16
NUC2	1H
PCPD2	80.00 usec
PL2	-1.00 dB
PL12	14.26 dB
PL13	14.46 dB
PL2W	13.18669796 W
PL12W	0.39276794 W
PL13W	0.37509048 W
SFO2	400.1716007 MHz
SI	32768
SF	100.6228270 MHz
WDW	EM
SSB	0
LB	1.00 Hz
GB	0
PC	1.40

4e ^{13}C NMR

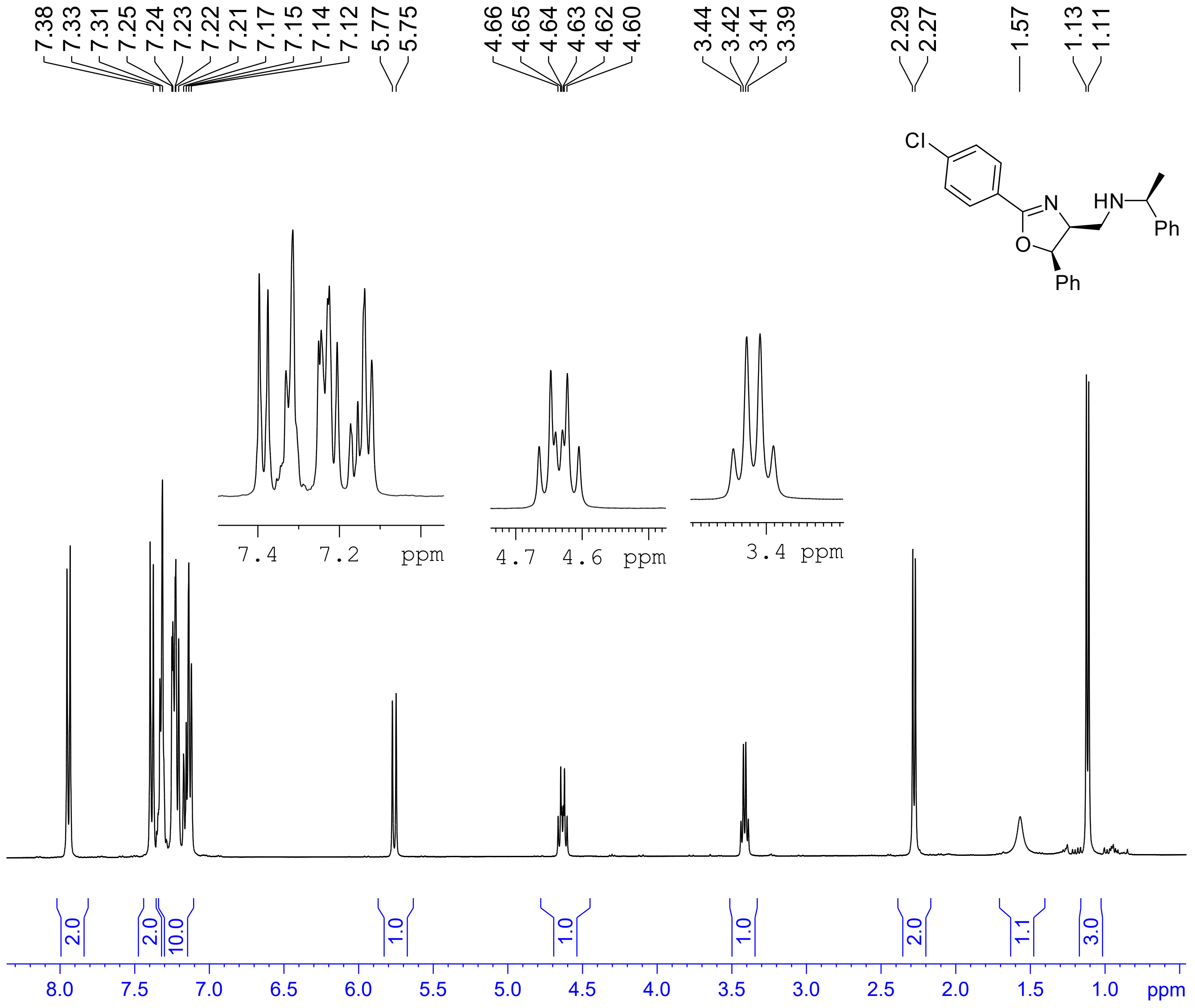

BRUKER

NAME	4f ZX-pCl-oxazoline-3
EXPNO	1
PROCNO	1
Date_	20150122
Time	17.42
INSTRUM	spect
PROBHD	5 mm PABBO BB-
PULPROG	zg30
TD	65536
SOLVENT	CDCl3
NS	16
DS	2
SWH	8223.685 Hz
FIDRES	0.125483 Hz
AQ	3.9846387 sec
RG	32
DW	60.800 usec
DE	6.50 usec
TE	294.9 K
D1	1.00000000 sec
TD0	1

======== CHANNEL f1 ========

NUC1	1H
P1	13.80 usec
PL1	-1.00 dB
PL1W	13.18669796 W
SFO1	400.1724712 MHz
SI	32768
SF	400.1700166 MHz
WDW	EM
SSB	0
LB	0.30 Hz
GB	0
PC	1.00

4f ^{1}H NMR

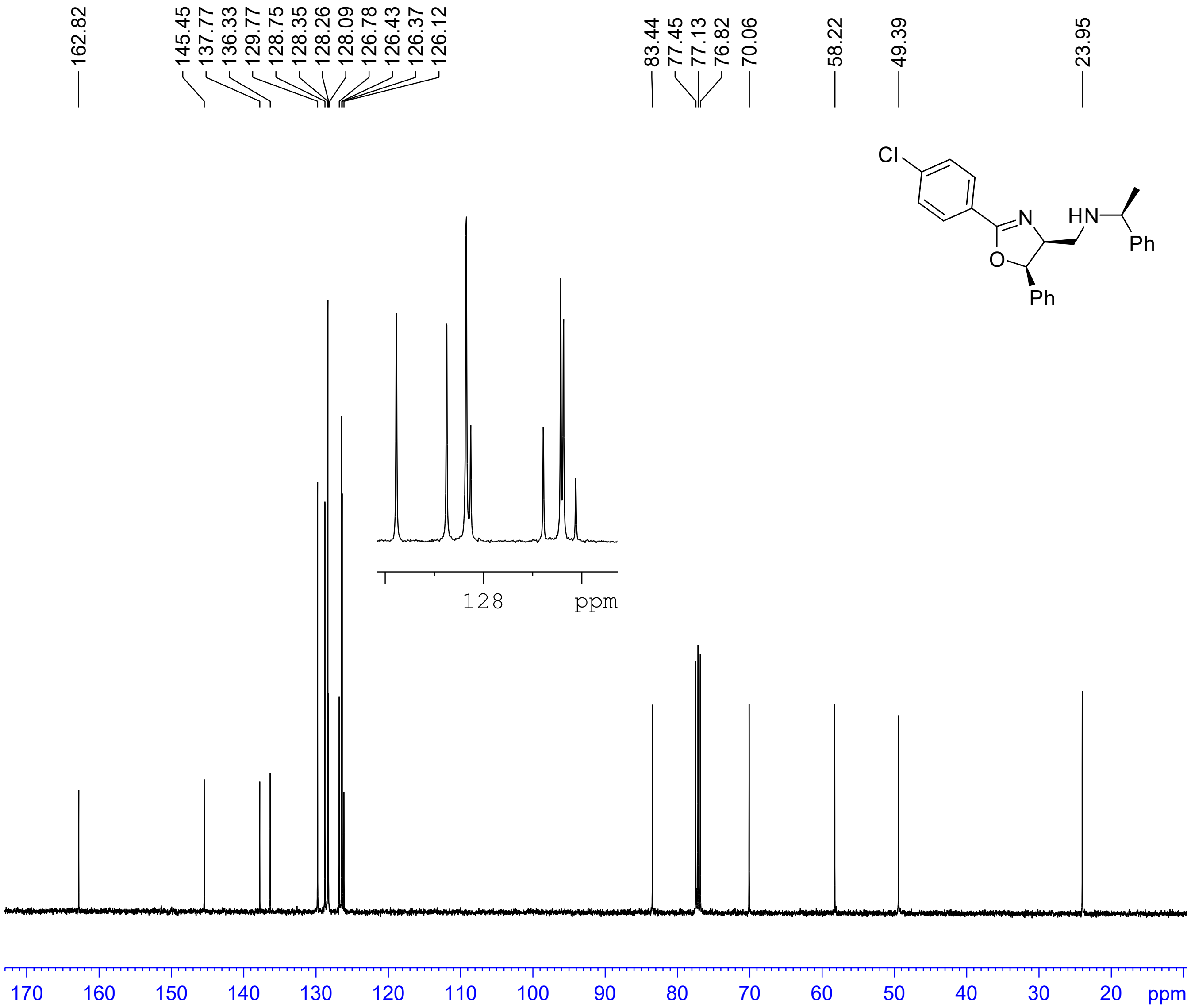

4f ^{13}C NMR

```
NAME       4f ZX-pCl-oxazoline-C13
EXPNO                     1
PROCNO                    1
Date_              20150123
Time                  14.02
INSTRUM               spect
PROBHD     5 mm PABBO BB-
PULPROG             zgpg30
TD                    65536
SOLVENT               CDCl3
NS                      153
DS                        4
SWH               24038.461 Hz
FIDRES             0.366798 Hz
AQ                1.3631988 sec
RG                      203
DW                   20.800 usec
DE                     6.50 usec
TE                    296.2 K
D1               2.00000000 sec
D11              0.03000000 sec
TD0                       1

======== CHANNEL f1 ========
NUC1                    13C
P1                     8.50 usec
PL1                   -2.00 dB
PL1W            57.32743073 W
SFO1            100.6328888 MHz

======== CHANNEL f2 ========
CPDPRG2             waltz16
NUC2                     1H
PCPD2                 80.00 usec
PL2                   -1.00 dB
PL12                  14.26 dB
PL13                  14.46 dB
PL2W            13.18669796 W
PL12W            0.39276794 W
PL13W            0.37509048 W
SFO2            400.1716007 MHz
SI                    32768
SF              100.6228270 MHz
WDW                      EM
SSB                       0
LB                     1.00 Hz
GB                        0
PC                     1.40
```

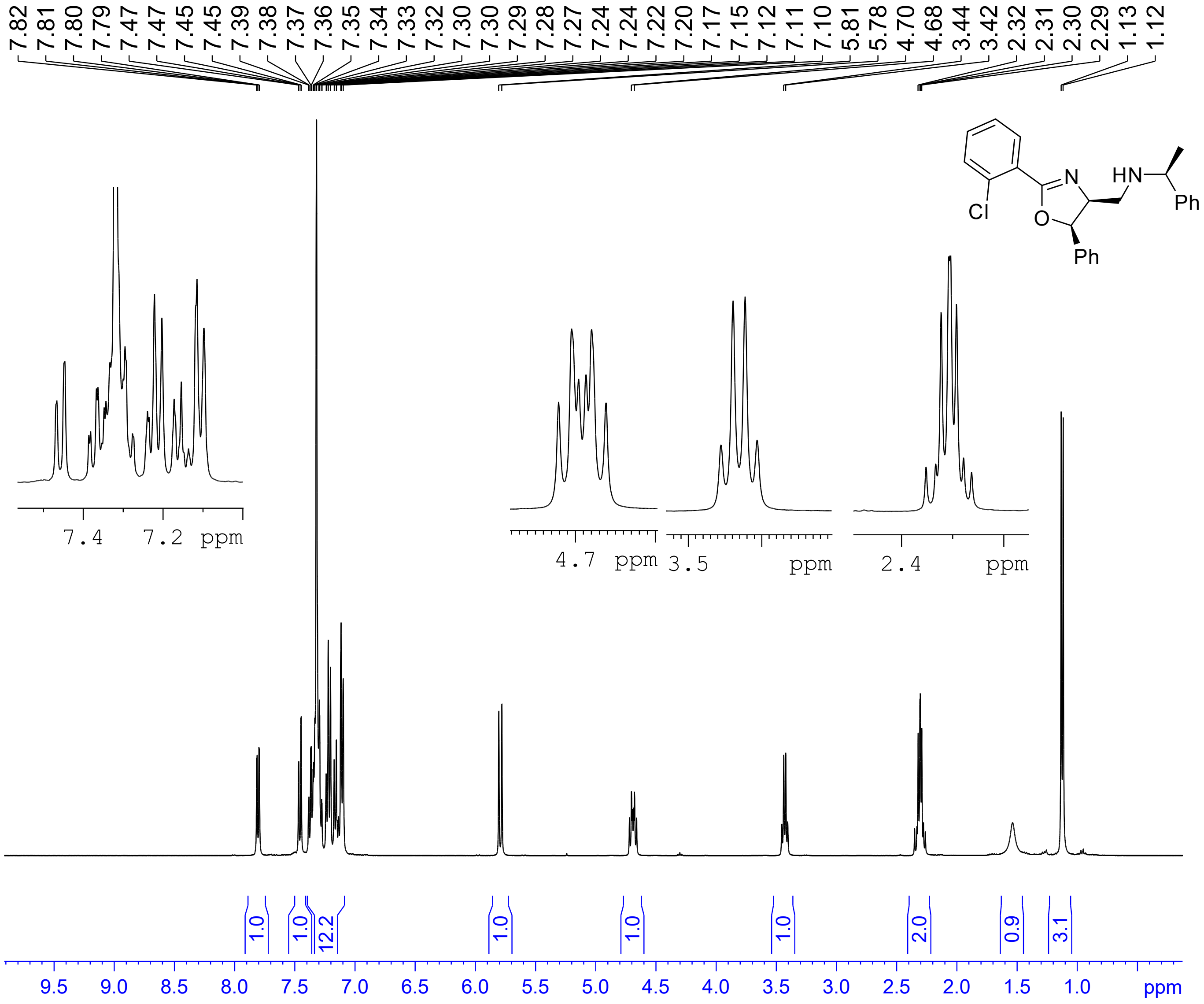

BRUKER

NAME	4g ZX-oCl-oxazoline
EXPNO	1
PROCNO	1
Date_	20150122
Time	13.51
INSTRUM	spect
PROBHD	5 mm PABBO BB-
PULPROG	zg30
TD	65536
SOLVENT	CDCl3
NS	16
DS	2
SWH	8223.685 Hz
FIDRES	0.125483 Hz
AQ	3.9846387 sec
RG	36
DW	60.800 usec
DE	6.50 usec
TE	297.2 K
D1	1.00000000 sec
TD0	1
======== CHANNEL f1 ========	
NUC1	1H
P1	13.80 usec
PL1	-1.00 dB
PL1W	13.18669796 W
SFO1	400.1724712 MHz
SI	32768
SF	400.1700140 MHz
WDW	EM
SSB	0
LB	0.30 Hz
GB	0
PC	1.00

4g ^{1}H NMR

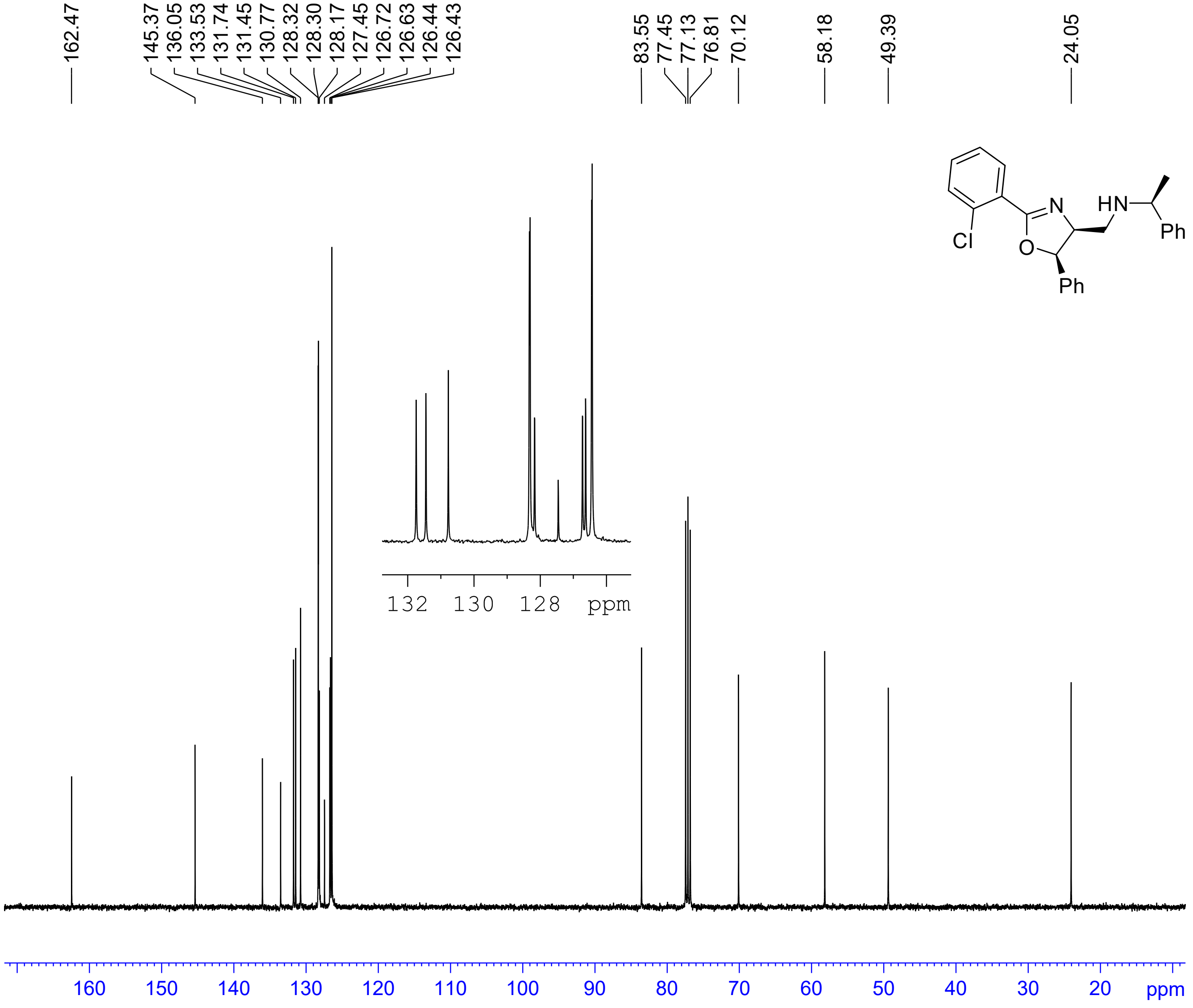

4g ^{13}C NMR

```
NAME       4g ZX-oCl-oxazoline-C13
EXPNO                     1
PROCNO                    1
Date_              20150122
Time                  18.22
INSTRUM               spect
PROBHD     5 mm PABBO BB-
PULPROG              zgpg30
TD                    65536
SOLVENT               CDCl3
NS                      250
DS                        4
SWH               24038.461 Hz
FIDRES             0.366798 Hz
AQ                1.3631988 sec
RG                      203
DW                   20.800 usec
DE                     6.50 usec
TE                    296.0 K
D1               2.00000000 sec
D11              0.03000000 sec
TD0                       1

======== CHANNEL f1 ========
NUC1                    13C
P1                     8.50 usec
PL1                   -2.00 dB
PL1W            57.32743073 W
SFO1            100.6328888 MHz

======== CHANNEL f2 ========
CPDPRG2             waltz16
NUC2                     1H
PCPD2                 80.00 usec
PL2                   -1.00 dB
PL12                  14.26 dB
PL13                  14.46 dB
PL2W            13.18669796 W
PL12W            0.39276794 W
PL13W            0.37509048 W
SFO2            400.1716007 MHz
SI                    32768
SF              100.6228270 MHz
WDW                      EM
SSB                       0
LB                     1.00 Hz
GB                        0
PC                     1.40
```

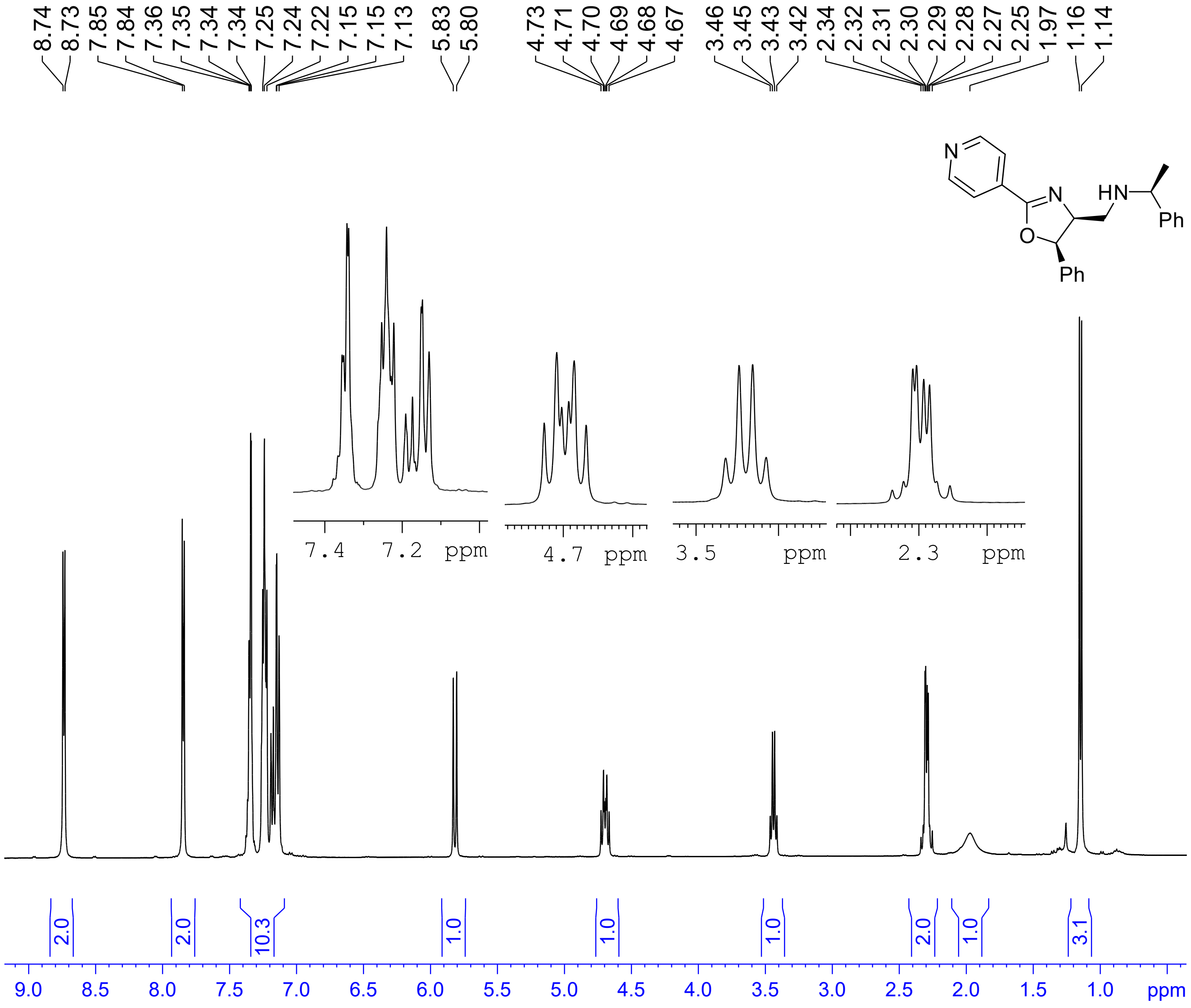


```
NAME       4h ZX-pPy-oxazoline-1
EXPNO                    1
PROCNO                   1
Date_             20150115
Time                  9.31
INSTRUM              spect
PROBHD     5 mm PABBO BB-
PULPROG               zg30
TD                   65536
SOLVENT              CDCl3
NS                      16
DS                       2
SWH               8223.685 Hz
FIDRES            0.125483 Hz
AQ               3.9846387 sec
RG                      64
DW                  60.800 usec
DE                    6.50 usec
TE                   292.9 K
D1              1.00000000 sec
TD0                      1

======== CHANNEL f1 ========
NUC1                    1H
P1                   13.80 usec
PL1                  -1.00 dB
PL1W           13.18669796 W
SFO1           400.1724712 MHz
SI                   32768
SF             400.1700029 MHz
WDW                     EM
SSB                      0
LB                    0.30 Hz
GB                       0
PC                    1.00
```

4h ^{1}H NMR

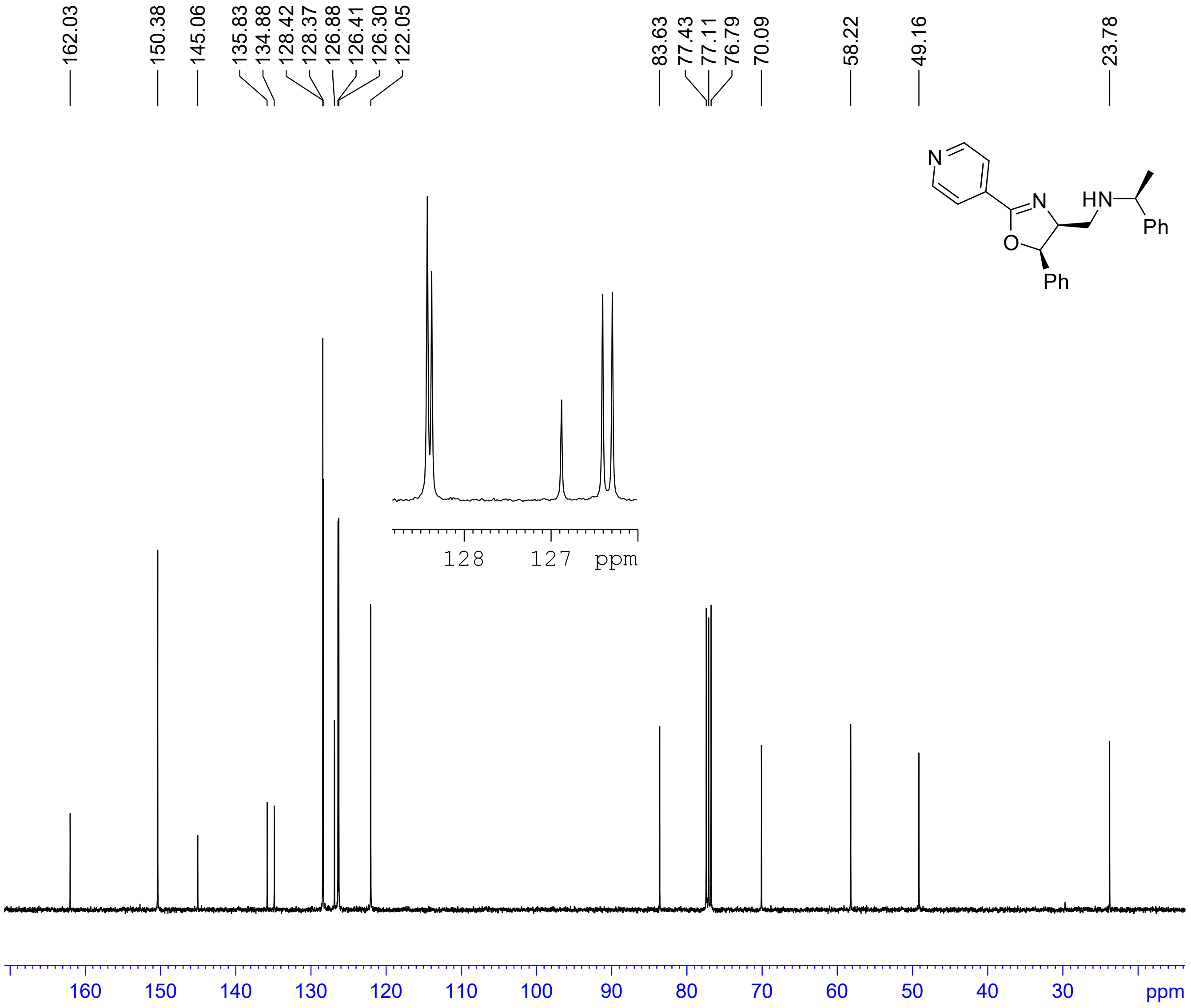

4h ^{13}C NMR

BRUKER

```
NAME        4h ZX-pPy-oxazoline-C13
EXPNO                 1
PROCNO                1
Date_          20150115
Time              17.42
INSTRUM           spect
PROBHD    5 mm PABBO BB-
PULPROG          zgpg30
TD                65536
SOLVENT           CDCl3
NS                  250
DS                    4
SWH           24038.461 Hz
FIDRES         0.366798 Hz
AQ            1.3631988 sec
RG                  203
DW               20.800 usec
DE                 6.50 usec
TE                293.5 K
D1           2.00000000 sec
D11          0.03000000 sec
TD0                   1

======== CHANNEL f1 ========
NUC1                13C
P1                 8.50 usec
PL1               -2.00 dB
PL1W        57.32743073 W
SFO1        100.6328888 MHz

======== CHANNEL f2 ========
CPDPRG2         waltz16
NUC2                 1H
PCPD2             80.00 usec
PL2               -1.00 dB
PL12              14.26 dB
PL13              14.46 dB
PL2W        13.18669796 W
PL12W        0.39276794 W
PL13W        0.37509048 W
SFO2        400.1716007 MHz
SI                32768
SF          100.6228270 MHz
WDW                  EM
SSB                   0
LB                 1.00 Hz
GB                    0
PC                 1.40
```

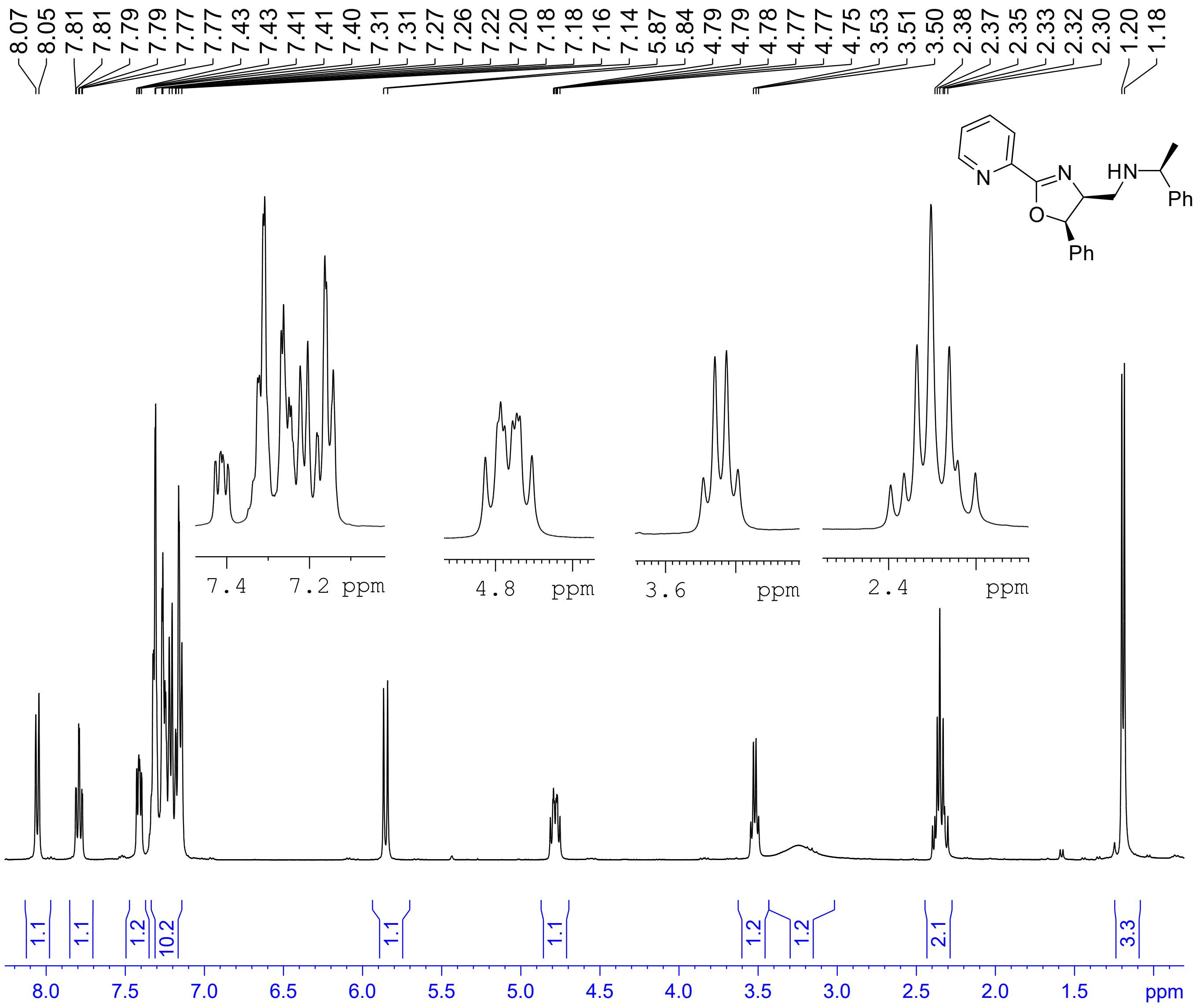


```
NAME       4i ZX-oPy-oxazoline
EXPNO                    1
PROCNO                   1
Date_             20150203
Time                 16.19
INSTRUM              spect
PROBHD     5 mm PABBO BB-
PULPROG               zg30
TD                   65536
SOLVENT              CDCl3
NS                      16
DS                       2
SWH               8223.685 Hz
FIDRES            0.125483 Hz
AQ               3.9846387 sec
RG                     101
DW                  60.800 usec
DE                    6.50 usec
TE                   294.7 K
D1              1.00000000 sec
TD0                      1

======== CHANNEL f1 ========
NUC1                    1H
P1                   13.80 usec
PL1                  -1.00 dB
PL1W           13.18669796 W
SFO1           400.1724712 MHz
SI                   32768
SF             400.1700031 MHz
WDW                     EM
SSB                      0
LB                    0.30 Hz
GB                       0
PC                    1.00
```

4i ^{1}H NMR

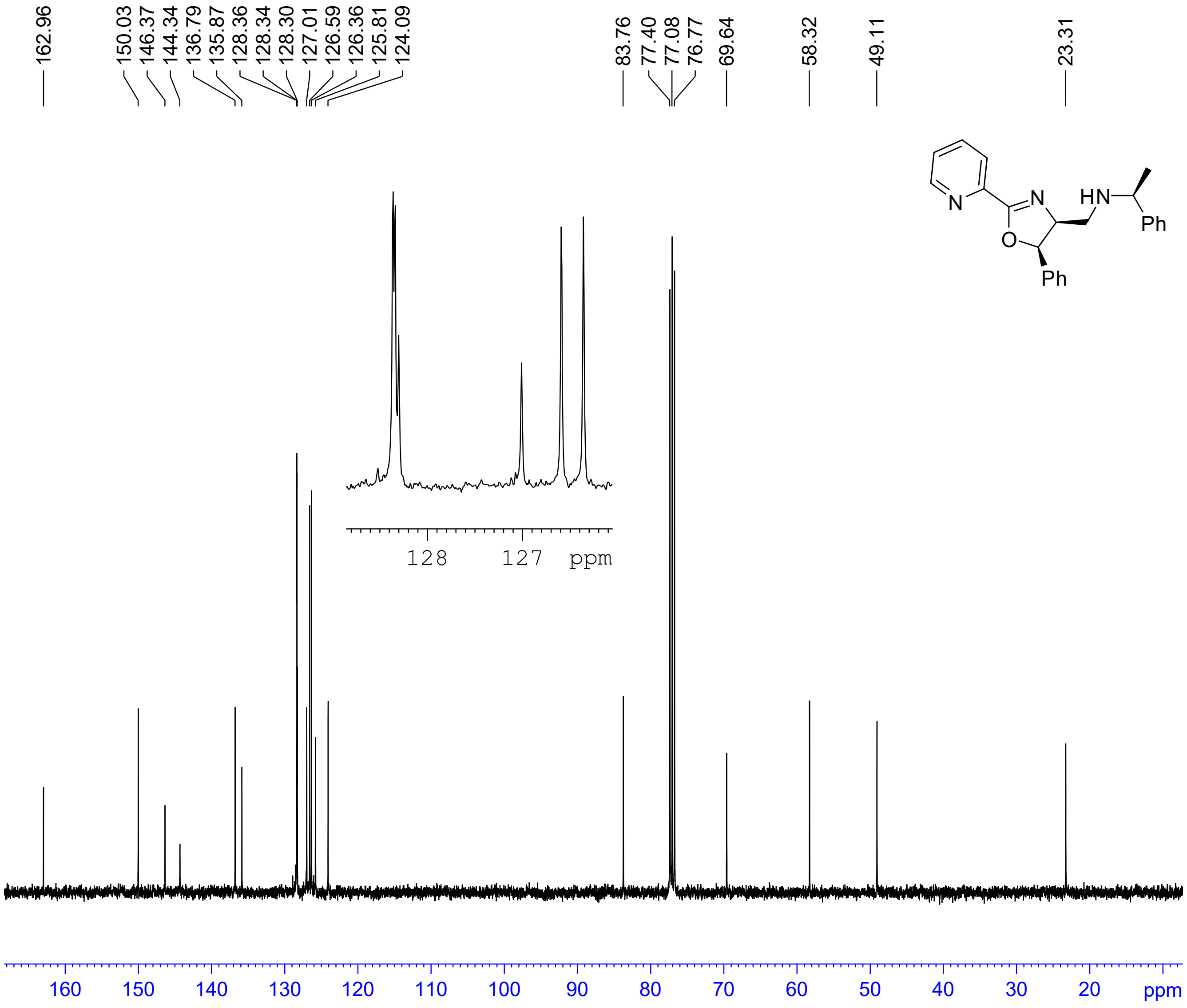


```
NAME      4i ZX-oPy-oxazoline C13
EXPNO                       1
PROCNO                      1
Date_                20150203
Time                    22.01
INSTRUM                 spect
PROBHD      5 mm PABBO BB-
PULPROG               zgpg30
TD                      65536
SOLVENT                 CDCl3
NS                        167
DS                          4
SWH             24038.461 Hz
FIDRES           0.366798 Hz
AQ              1.3631988 sec
RG                        203
DW                 20.800 usec
DE                  6.50 usec
TE                  293.8 K
D1             2.00000000 sec
D11            0.03000000 sec
TD0                         1

======== CHANNEL f1 ========
NUC1                      13C
P1                  8.50 usec
PL1                 -2.00 dB
PL1W            57.32743073 W
SFO1          100.6328888 MHz

======== CHANNEL f2 ========
CPDPRG2               waltz16
NUC2                       1H
PCPD2              80.00 usec
PL2                 -1.00 dB
PL12                14.26 dB
PL13                14.46 dB
PL2W            13.18669796 W
PL12W            0.39276794 W
PL13W            0.37509048 W
SFO2          400.1716007 MHz
SI                      32768
SF            100.6228270 MHz
WDW                        EM
SSB                         0
LB                    1.00 Hz
GB                          0
PC                       1.40
```

4i ^{13}C NMR

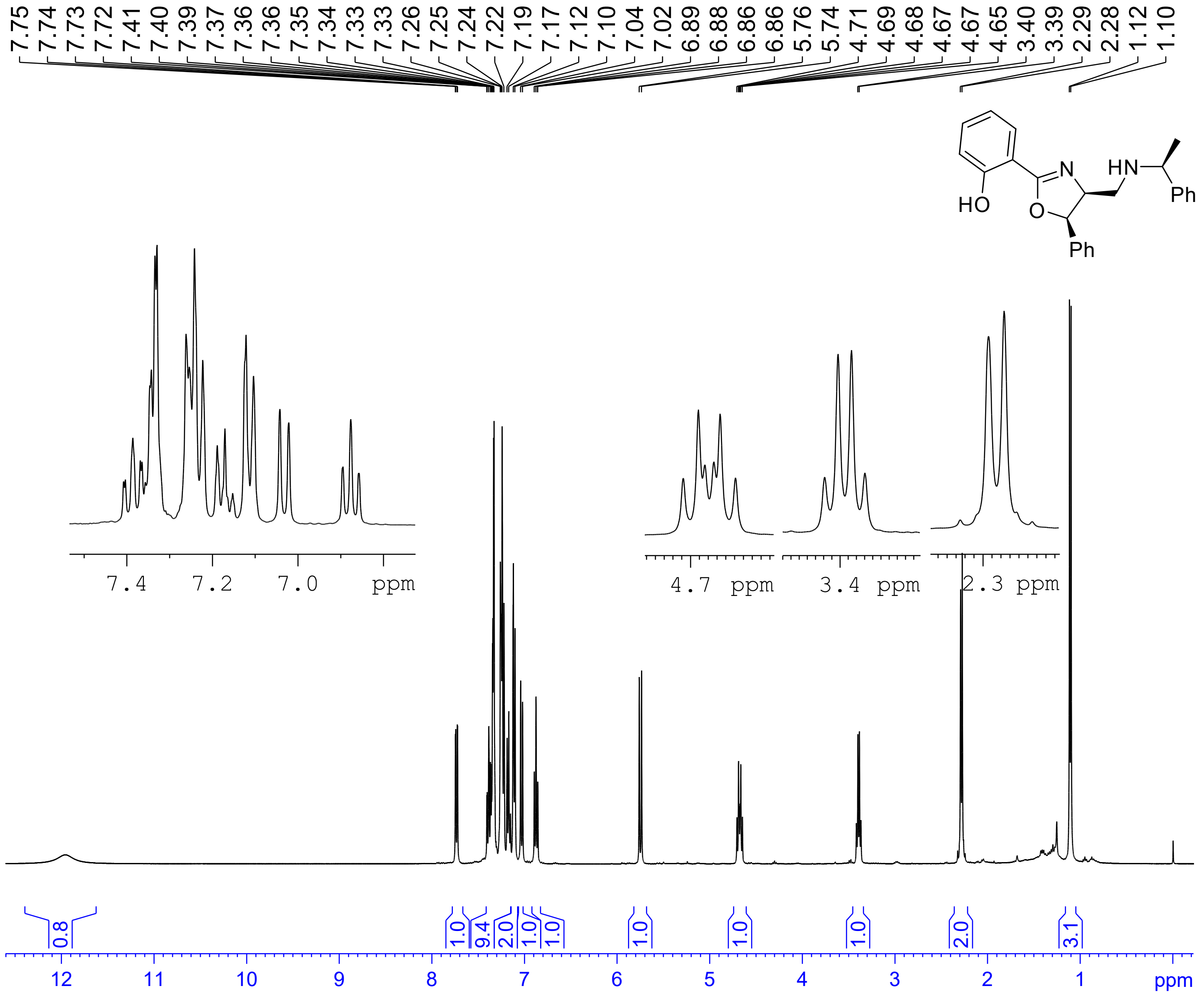

4j ^{1}H NMR

```
NAME       4j ZX-oOH-oxazoline-1
EXPNO                      1
PROCNO                     1
Date_               20141211
Time                   20.33
INSTRUM                spect
PROBHD     5 mm PABBO BB-
PULPROG                 zg30
TD                     65536
SOLVENT                CDCl3
NS                        16
DS                         2
SWH                 8223.685 Hz
FIDRES              0.125483 Hz
AQ                 3.9846387 sec
RG                        32
DW                    60.800 usec
DE                      6.50 usec
TE                     292.4 K
D1                1.00000000 sec
TD0                        1

======== CHANNEL f1 ========
NUC1                      1H
P1                     13.80 usec
PL1                    -1.00 dB
PL1W             13.18669796 W
SFO1             400.1724712 MHz
SI                     32768
SF               400.1700204 MHz
WDW                       EM
SSB                        0
LB                      0.30 Hz
GB                         0
PC                      1.00
```

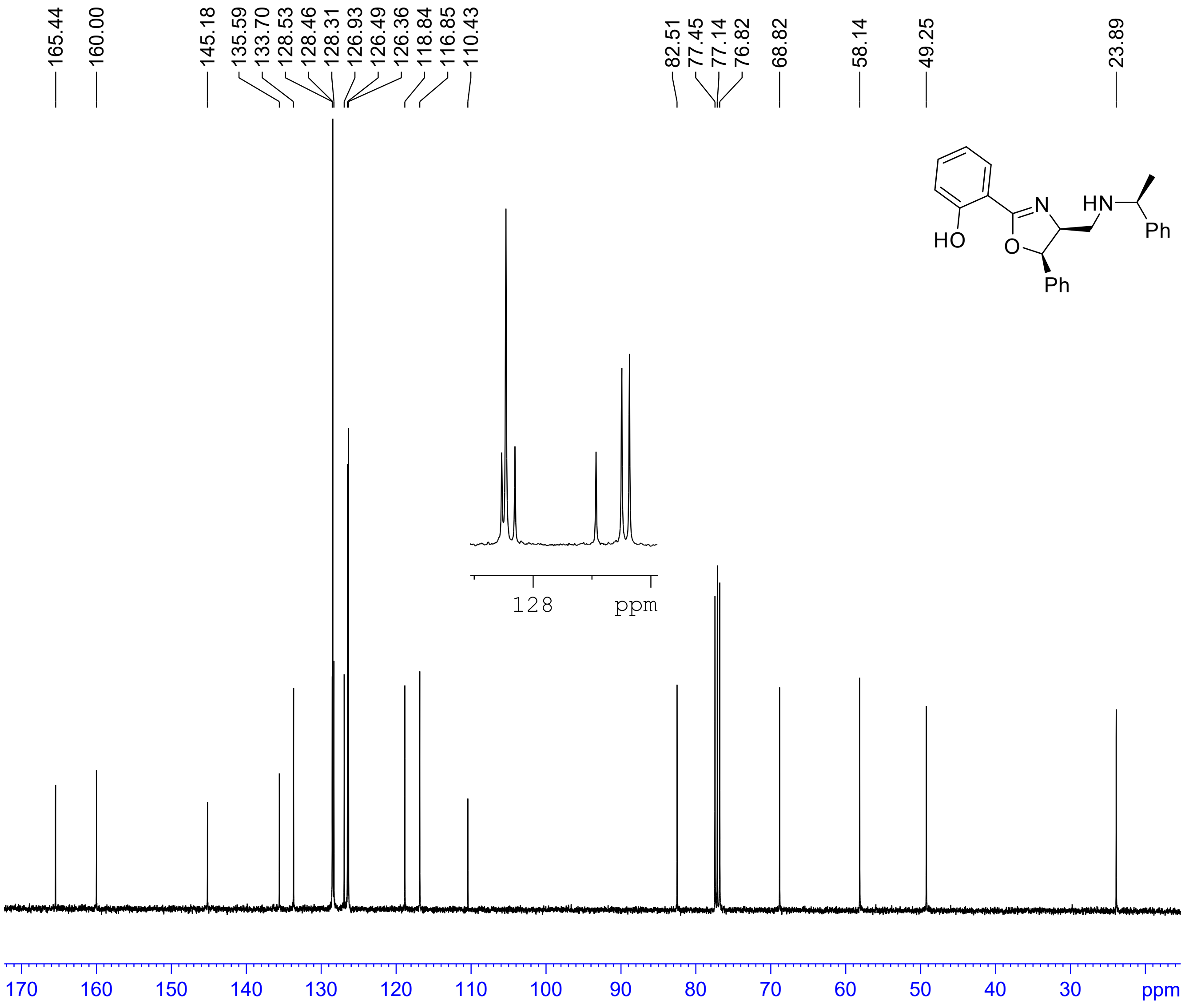

NAME	4j ZX-oOH-oxazoline-C13
EXPNO	1
PROCNO	1
Date_	20141211
Time	20.50
INSTRUM	spect
PROBHD	5 mm PABBO BB-
PULPROG	zgpg30
TD	65536
SOLVENT	CDCl3
NS	133
DS	4
SWH	24038.461 Hz
FIDRES	0.366798 Hz
AQ	1.3631988 sec
RG	203
DW	20.800 usec
DE	6.50 usec
TE	292.9 K
D1	2.00000000 sec
D11	0.03000000 sec
TD0	1

======== CHANNEL f1 ========

NUC1	13C
P1	8.50 usec
PL1	-2.00 dB
PL1W	57.32743073 W
SFO1	100.6328888 MHz

======== CHANNEL f2 ========

CPDPRG2	waltz16
NUC2	1H
PCPD2	80.00 usec
PL2	-1.00 dB
PL12	14.26 dB
PL13	14.46 dB
PL2W	13.18669796 W
PL12W	0.39276794 W
PL13W	0.37509048 W
SFO2	400.1716007 MHz
SI	32768
SF	100.6228270 MHz
WDW	EM
SSB	0
LB	1.00 Hz
GB	0
PC	1.40

4j ^{13}C NMR

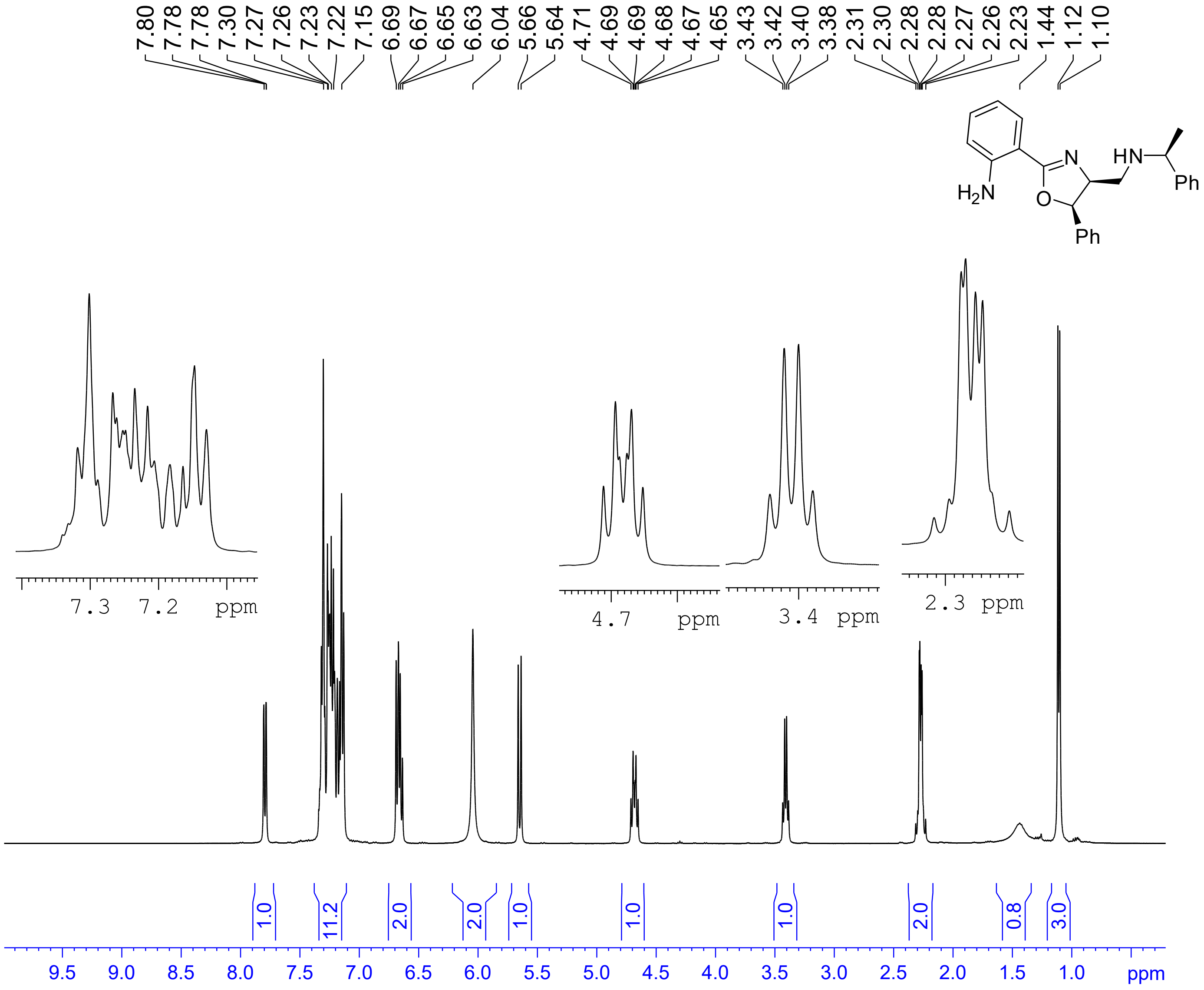


```
NAME       4k ZX-oNH2-oxazoline
EXPNO                     1
PROCNO                    1
Date_              20150122
Time                   9.08
INSTRUM               spect
PROBHD    5 mm PABBO BB-
PULPROG                zg30
TD                    65536
SOLVENT               CDCl3
NS                       16
DS                        2
SWH               8223.685 Hz
FIDRES            0.125483 Hz
AQ               3.9846387 sec
RG                       32
DW                   60.800 usec
DE                     6.50 usec
TE                    293.6 K
D1               1.00000000 sec
TD0                       1

======== CHANNEL f1 ========
NUC1                     1H
P1                    13.80 usec
PL1                   -1.00 dB
PL1W            13.18669796 W
SFO1            400.1724712 MHz
SI                    32768
SF              400.1700284 MHz
WDW                      EM
SSB                       0
LB                     0.30 Hz
GB                        0
PC                     1.00
```

4k ^{1}H NMR

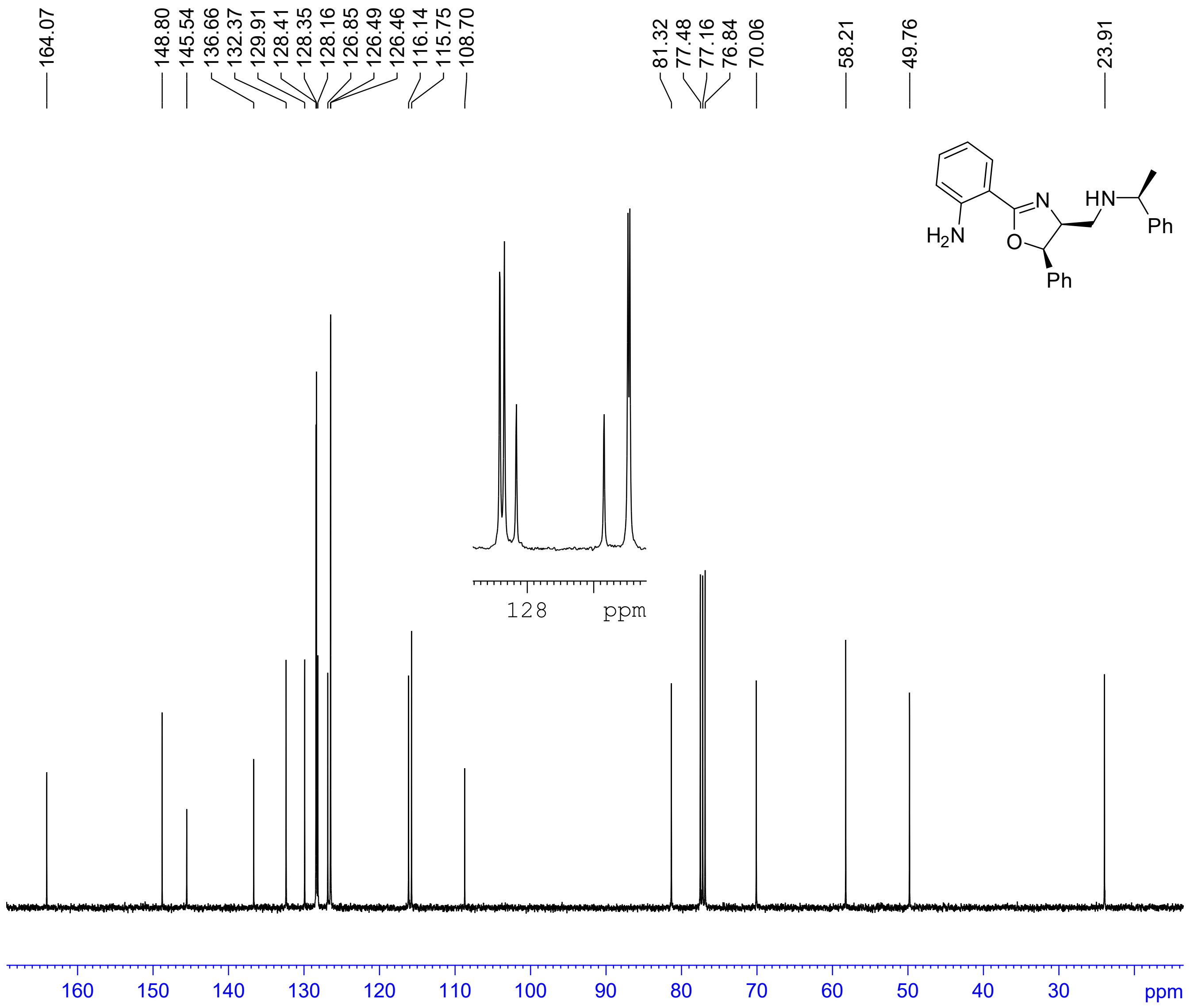


```
NAME          4k ZX-oNH2-oxazoline-C13
EXPNO                        1
PROCNO                       1
Date_                 20150122
Time                     18.40
INSTRUM                  spect
PROBHD        5 mm PABBO BB-
PULPROG                zgpg30
TD                       65536
SOLVENT                  CDCl3
NS                         150
DS                           4
SWH                  24038.461 Hz
FIDRES                0.366798 Hz
AQ                   1.3631988 sec
RG                         203
DW                      20.800 usec
DE                        6.50 usec
TE                       295.8 K
D1                  2.00000000 sec
D11                 0.03000000 sec
TD0                          1

======== CHANNEL f1 ========
NUC1                       13C
P1                        8.50 usec
PL1                      -2.00 dB
PL1W               57.32743073 W
SFO1               100.6328888 MHz

======== CHANNEL f2 ========
CPDPRG2                waltz16
NUC2                        1H
PCPD2                    80.00 usec
PL2                      -1.00 dB
PL12                     14.26 dB
PL13                     14.46 dB
PL2W               13.18669796 W
PL12W               0.39276794 W
PL13W               0.37509048 W
SFO2               400.1716007 MHz
SI                       32768
SF                 100.6228270 MHz
WDW                         EM
SSB                          0
LB                        1.00 Hz
GB                           0
PC                        1.40
```

4k ^{13}C NMR

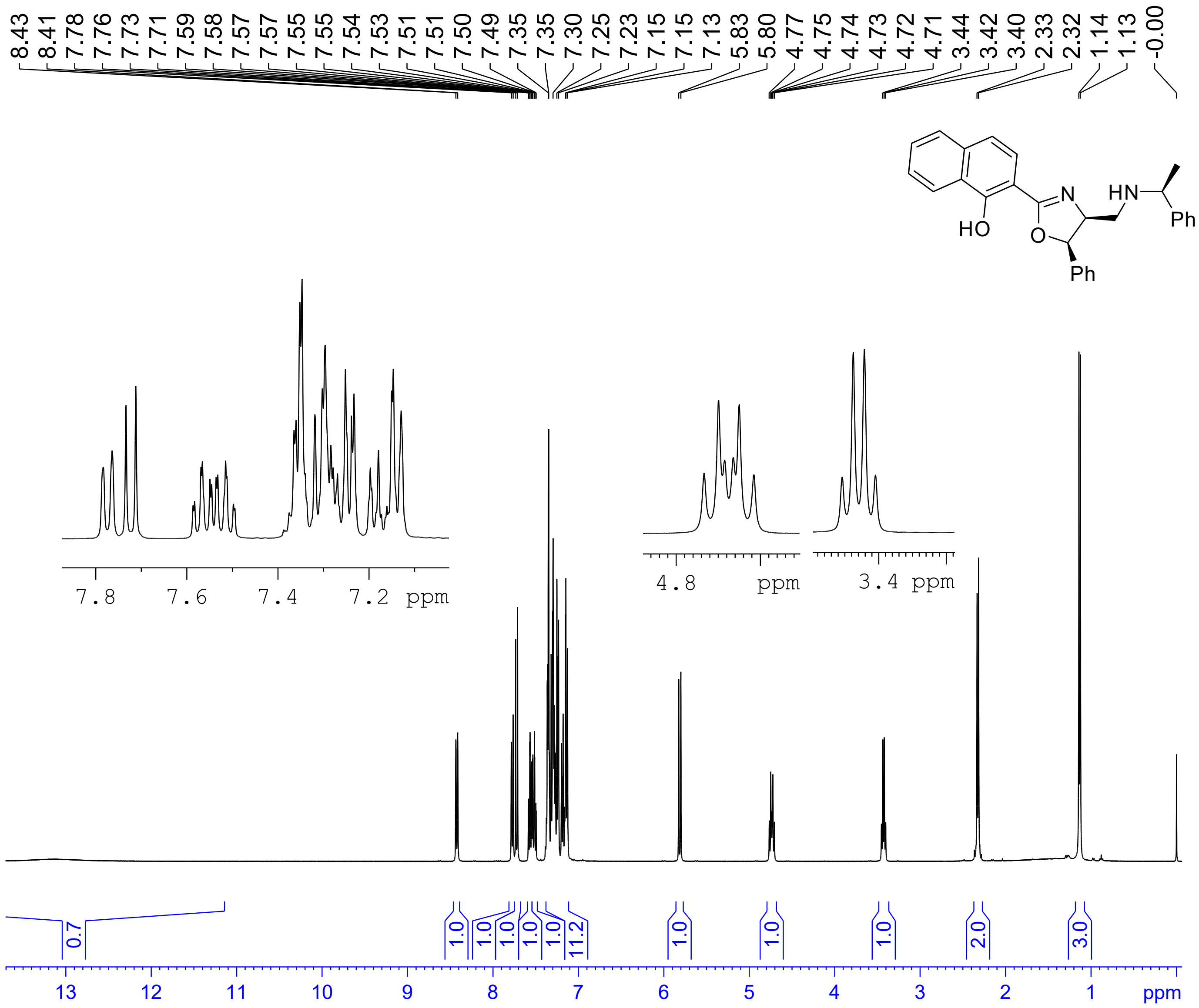

NAME 41 ZX-1OH-2naphthyl-oxazoline
EXPNO 1
PROCNO 1
Date_ 20141124
Time 17.11
INSTRUM spect
PROBHD 5 mm PABBO BB-
PULPROG zg30
TD 65536
SOLVENT CDCl3
NS 16
DS 2
SWH 8223.685 Hz
FIDRES 0.125483 Hz
AQ 3.9846387 sec
RG 114
DW 60.800 usec
DE 6.50 usec
TE 295.6 K
D1 1.00000000 sec
TD0 1

======== CHANNEL f1 ========
NUC1 1H
P1 13.80 usec
PL1 -1.00 dB
PL1W 13.18669796 W
SFO1 400.1724712 MHz
SI 32768
SF 400.1700124 MHz
WDW EM
SSB 0
LB 0.30 Hz
GB 0
PC 1.00

4l ^{1}H NMR

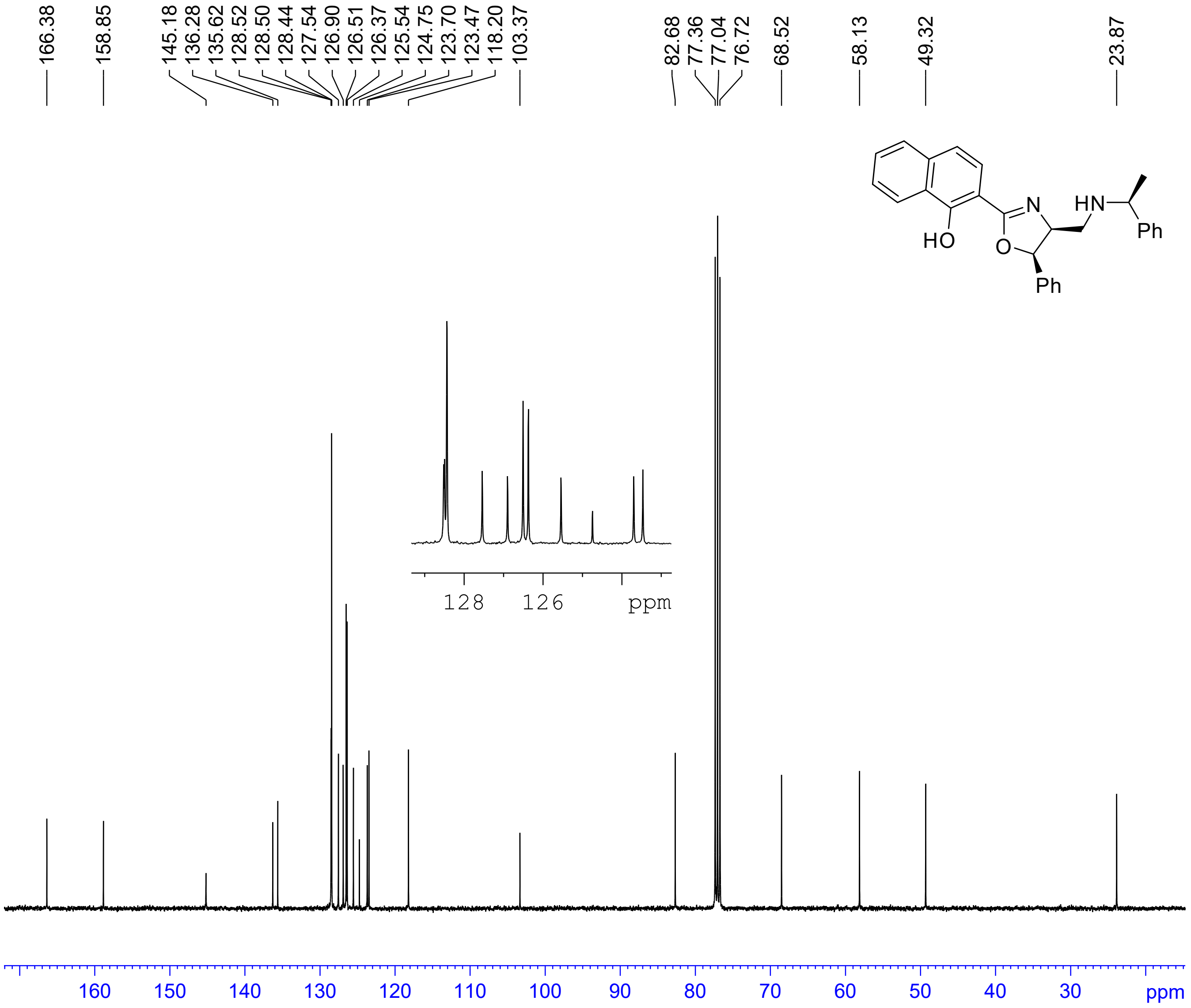

4l ^{13}C NMR

```
NAME      4l ZX-1OH-2naphthyl-oxazoline-C13
EXPNO                    1
PROCNO                   1
Date_             20141124
Time                 18.04
INSTRUM              spect
PROBHD    5 mm PABBO BB-
PULPROG            zgpg30
TD                  65536
SOLVENT             CDCl3
NS                   1024
DS                      4
SWH             24038.461 Hz
FIDRES           0.366798 Hz
AQ              1.3631988 sec
RG                    203
DW                 20.800 usec
DE                   6.50 usec
TE                  296.6 K
D1             2.00000000 sec
D11            0.03000000 sec
TD0                     1

======== CHANNEL f1 ========
NUC1                  13C
P1                   8.50 usec
PL1                 -2.00 dB
PL1W          57.32743073 W
SFO1          100.6328888 MHz

======== CHANNEL f2 ========
CPDPRG2           waltz16
NUC2                   1H
PCPD2               80.00 usec
PL2                 -1.00 dB
PL12                14.26 dB
PL13                14.46 dB
PL2W          13.18669796 W
PL12W          0.39276794 W
PL13W          0.37509048 W
SFO2          400.1716007 MHz
SI                  32768
SF            100.6228270 MHz
WDW                    EM
SSB                     0
LB                   1.00 Hz
GB                      0
PC                   1.40
```

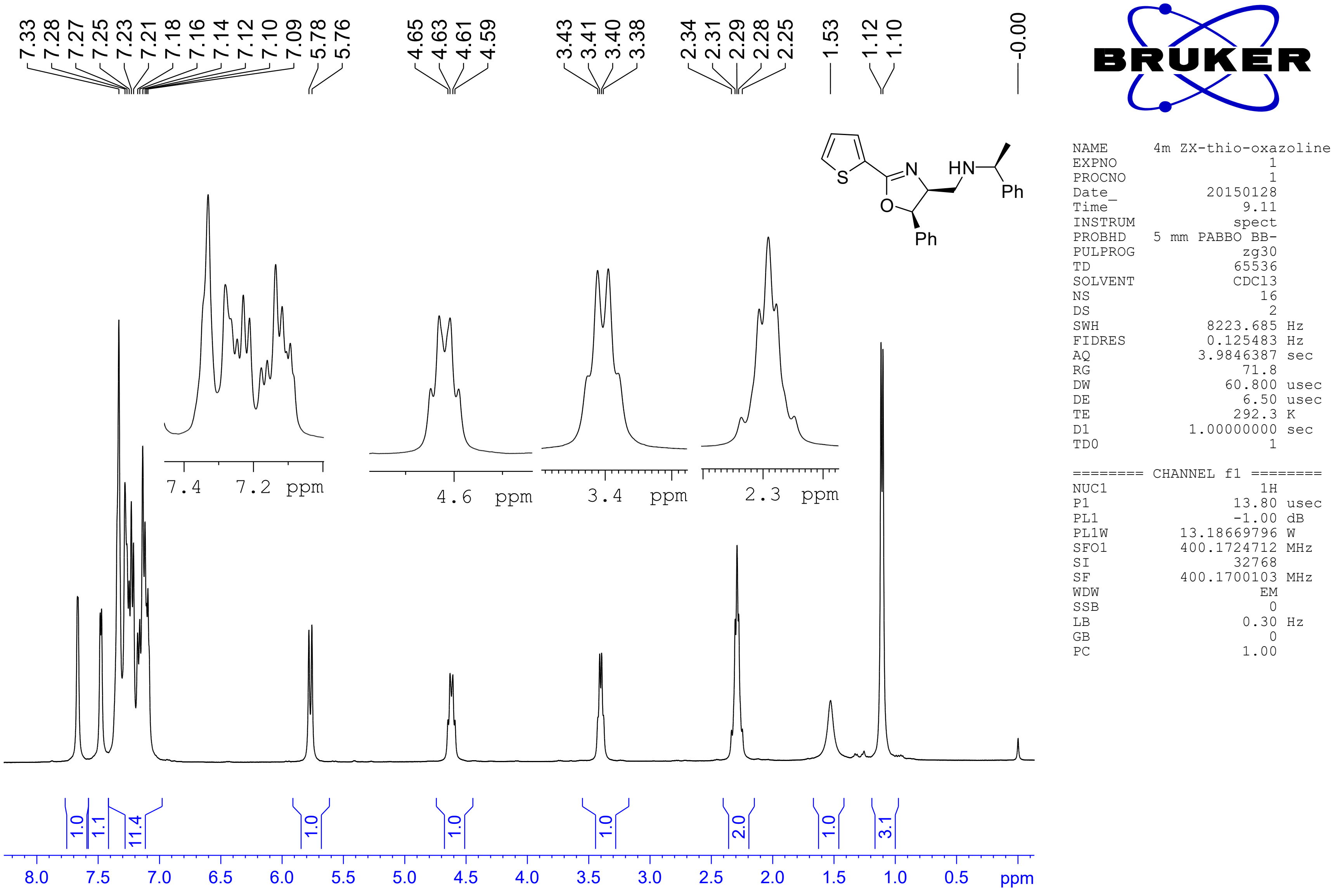
7.33 7.28 7.27 7.25 7.23 7.21 7.18 7.16 7.14 7.12 7.10 7.09 5.78 5.76
4.65 4.63 4.61 4.59
3.43 3.41 3.40 3.38
2.34 2.31 2.29 2.28 2.25
1.53
1.12 1.10
-0.00
HN
Ph
N
S
O
Ph
NAME 4m ZX-thio-oxazoline
EXPNO 1
PROCNO 1
Date_ 20150128
Time 9.11
INSTRUM spect
PROBHD 5 mm PABBO BB-
PULPROG zg30
TD 65536
SOLVENT CDCl3
NS 16
DS 2
SWH 8223.685 Hz
FIDRES 0.125483 Hz
AQ 3.9846387 sec
RG 71.8
DW 60.800 usec
DE 6.50 usec
TE 292.3 K
D1 1.00000000 sec
TD0 1
======== CHANNEL f1 ========
NUC1 1H
P1 13.80 usec
PL1 -1.00 dB
PL1W 13.18669796 W
SFO1 400.1724712 MHz
SI 32768
SF 400.1700103 MHz
WDW EM
SSB 0
LB 0.30 Hz
GB 0
PC 1.00
7.4 7.2 ppm
4.6 ppm
3.4 ppm
2.3 ppm
1.0 1.1 11.4 1.0 1.0 1.0 2.0 1.0 3.1
8.0 7.5 7.0 6.5 6.0 5.5 5.0 4.5 4.0 3.5 3.0 2.5 2.0 1.5 1.0 0.5 ppm

4m ^{1}H NMR

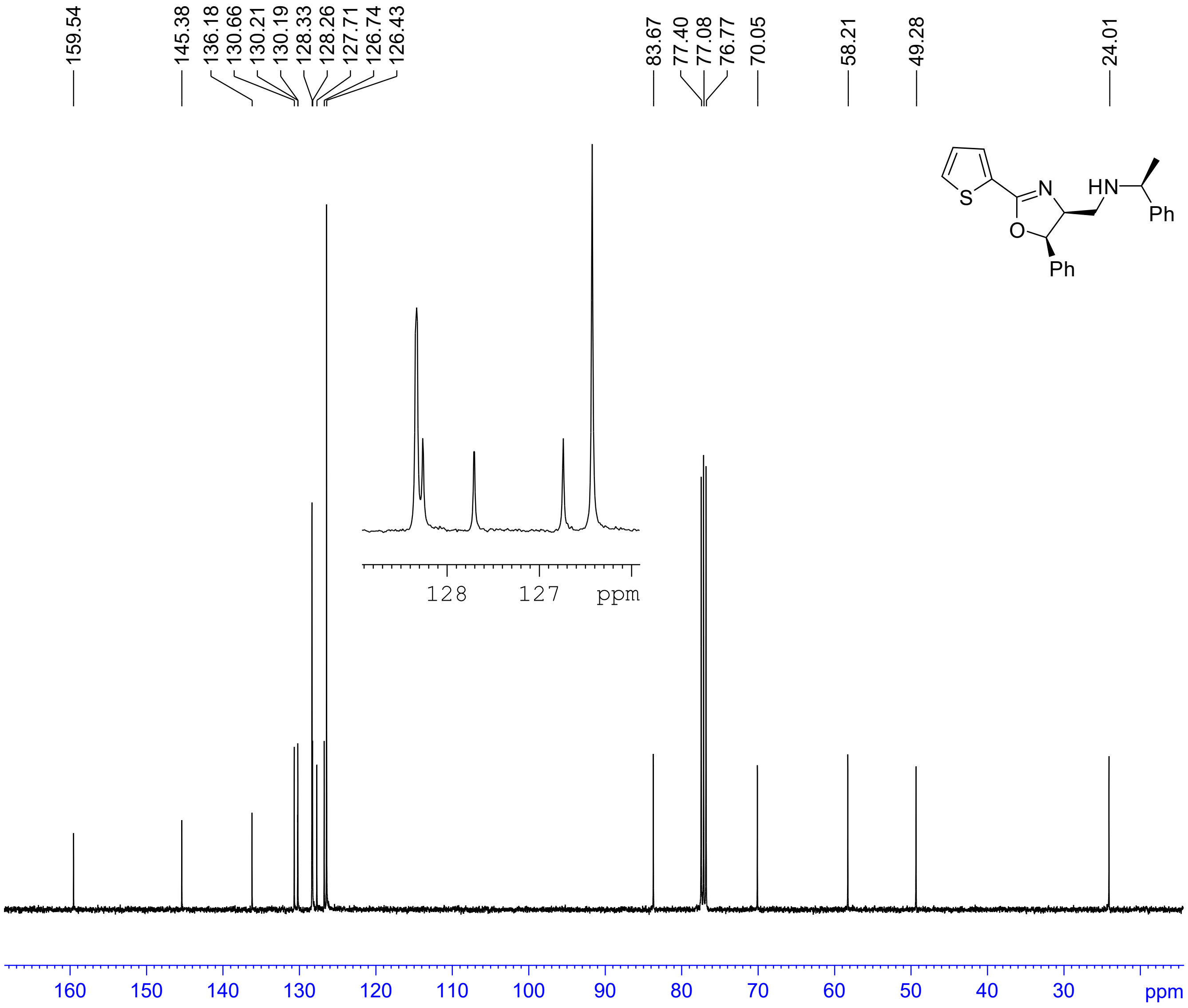

4m ^{13}C NMR

```
NAME        4m ZX-thio-oxazoline-C13
EXPNO                      1
PROCNO                     1
Date_               20150128
Time                   11.25
INSTRUM                spect
PROBHD      5 mm PABBO BB-
PULPROG              zgpg30
TD                     65536
SOLVENT                CDCl3
NS                       249
DS                         4
SWH                24038.461 Hz
FIDRES              0.366798 Hz
AQ                 1.3631988 sec
RG                       203
DW                    20.800 usec
DE                      6.50 usec
TE                     292.8 K
D1                2.00000000 sec
D11               0.03000000 sec
TD0                        1

======== CHANNEL f1 ========
NUC1                     13C
P1                      8.50 usec
PL1                    -2.00 dB
PL1W             57.32743073 W
SFO1             100.6328888 MHz

======== CHANNEL f2 ========
CPDPRG2              waltz16
NUC2                      1H
PCPD2                  80.00 usec
PL2                    -1.00 dB
PL12                   14.26 dB
PL13                   14.46 dB
PL2W             13.18669796 W
PL12W             0.39276794 W
PL13W             0.37509048 W
SFO2             400.1716007 MHz
SI                     32768
SF               100.6228270 MHz
WDW                       EM
SSB                        0
LB                      1.00 Hz
GB                         0
PC                      1.40
```

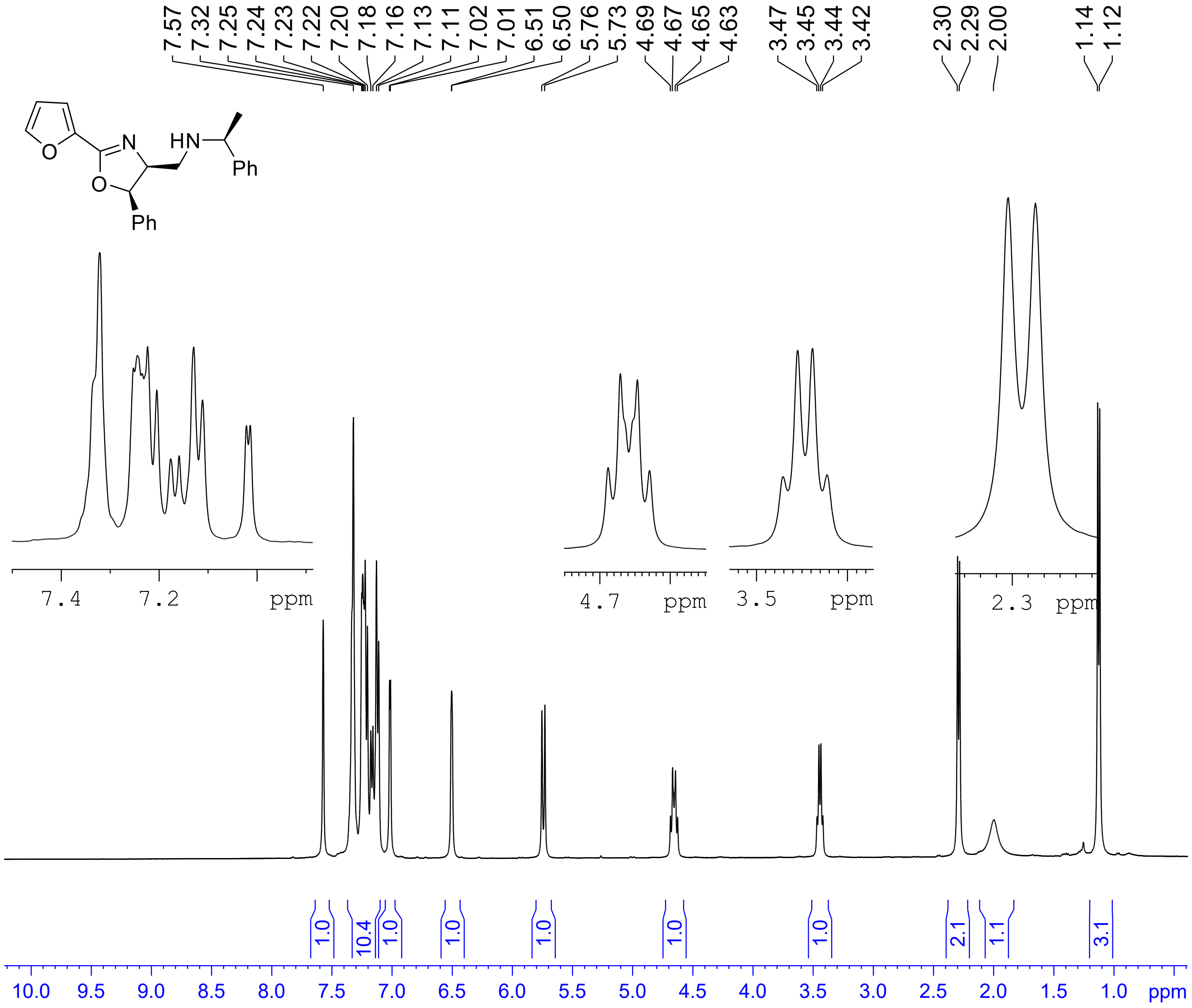

4n ^{1}H NMR

NAME	4n ZX-furan-oxazoline
EXPNO	1
PROCNO	1
Date_	20150113
Time	10.15
INSTRUM	spect
PROBHD	5 mm PABBO BB-
PULPROG	zg30
TD	65536
SOLVENT	CDCl3
NS	16
DS	2
SWH	8223.685 Hz
FIDRES	0.125483 Hz
AQ	3.9846387 sec
RG	40.3
DW	60.800 usec
DE	6.50 usec
TE	292.8 K
D1	1.00000000 sec
TD0	1

======== CHANNEL f1 ========

NUC1	1H
P1	13.80 usec
PL1	-1.00 dB
PL1W	13.18669796 W
SFO1	400.1724712 MHz
SI	32768
SF	400.1700050 MHz
WDW	EM
SSB	0
LB	0.30 Hz
GB	0
PC	1.00

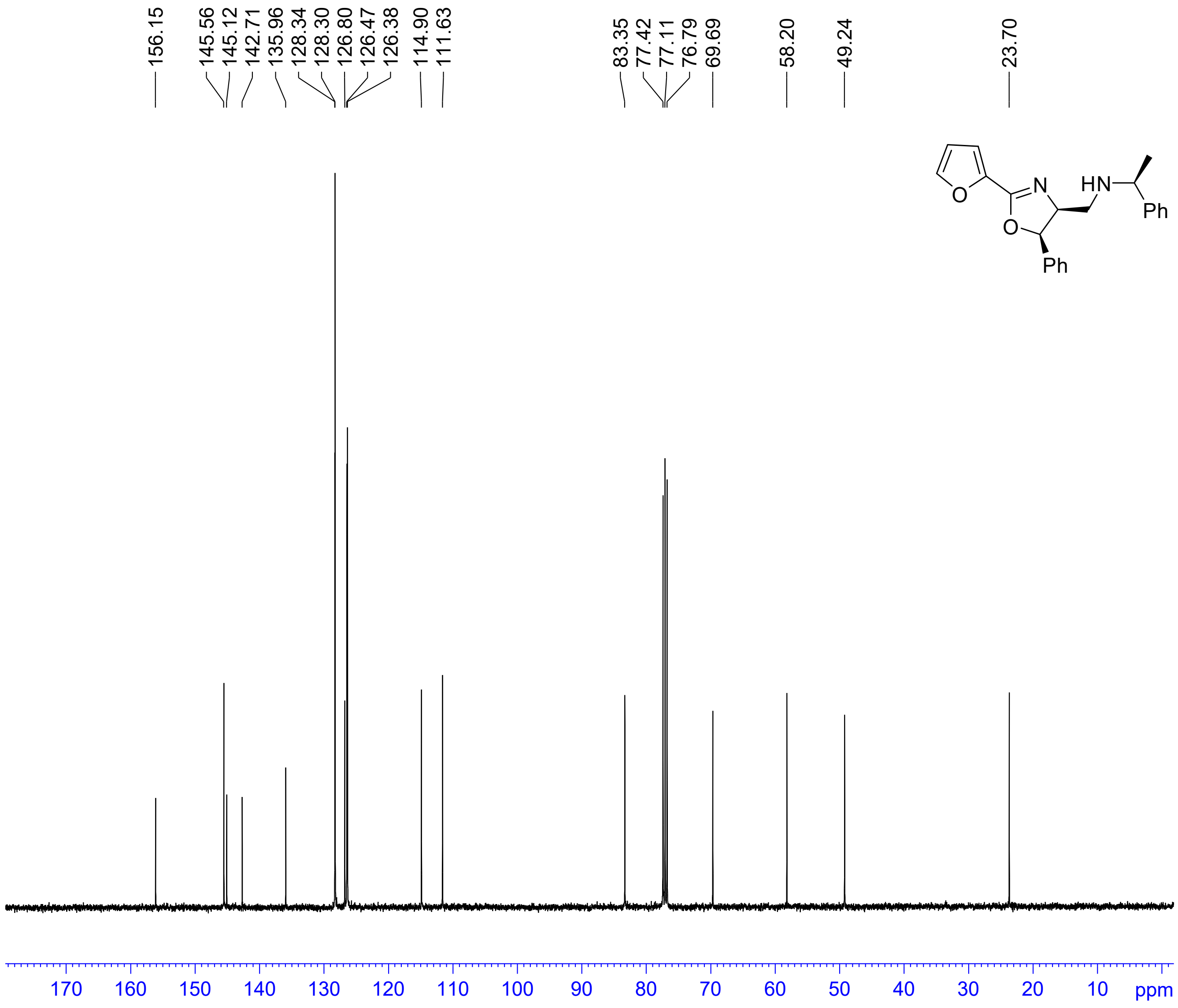

4n ^{13}C NMR

```
NAME         4n ZX-furan-oxazoline-C13
EXPNO                    1
PROCNO                   1
Date_             20150113
Time                 12.45
INSTRUM              spect
PROBHD     5 mm PABBO BB-
PULPROG            zgpg30
TD                   65536
SOLVENT              CDCl3
NS                     250
DS                       4
SWH              24038.461 Hz
FIDRES            0.366798 Hz
AQ               1.3631988 sec
RG                     203
DW                  20.800 usec
DE                    6.50 usec
TE                   295.5 K
D1              2.00000000 sec
D11             0.03000000 sec
TD0                      1

======== CHANNEL f1 ========
NUC1                   13C
P1                    8.50 usec
PL1                  -2.00 dB
PL1W           57.32743073 W
SFO1           100.6328888 MHz

======== CHANNEL f2 ========
CPDPRG2            waltz16
NUC2                    1H
PCPD2                80.00 usec
PL2                  -1.00 dB
PL12                 14.26 dB
PL13                 14.46 dB
PL2W           13.18669796 W
PL12W           0.39276794 W
PL13W           0.37509048 W
SFO2           400.1716007 MHz
SI                   32768
SF             100.6228270 MHz
WDW                     EM
SSB                      0
LB                    1.00 Hz
GB                       0
PC                    1.40
```

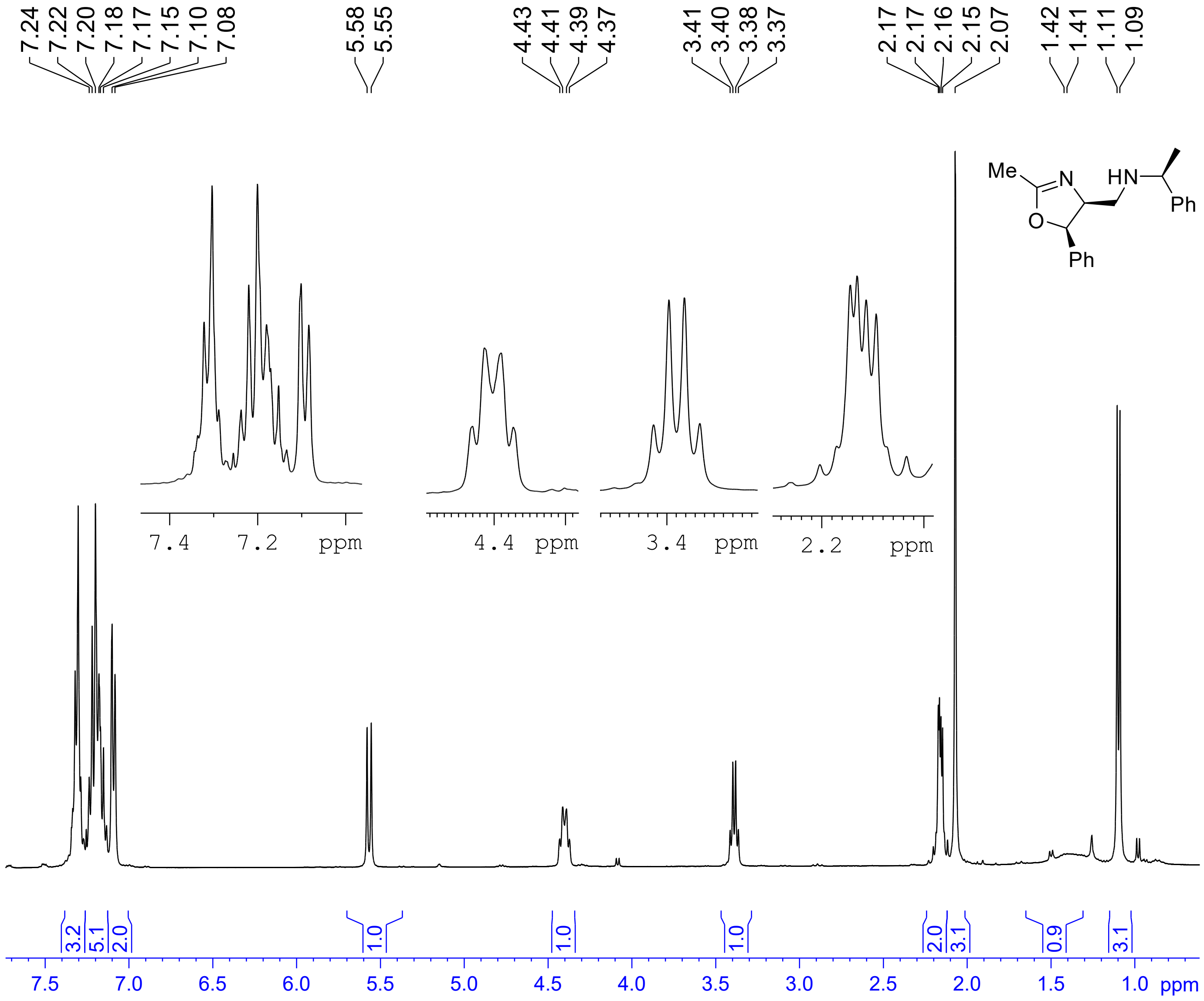

4o ^{1}H NMR

NAME	4o ZX-Me-oxazoline
EXPNO	1
PROCNO	1
Date_	20150206
Time	13.06
INSTRUM	spect
PROBHD	5 mm PABBO BB-
PULPROG	zg30
TD	65536
SOLVENT	CDCl3
NS	16
DS	2
SWH	8223.685 Hz
FIDRES	0.125483 Hz
AQ	3.9846387 sec
RG	32
DW	60.800 usec
DE	6.50 usec
TE	296.7 K
D1	1.00000000 sec
TD0	1

======== CHANNEL f1 ========

NUC1	1H
P1	13.80 usec
PL1	-1.00 dB
PL1W	13.18669796 W
SFO1	400.1724712 MHz
SI	32768
SF	400.1700061 MHz
WDW	EM
SSB	0
LB	0.30 Hz
GB	0
PC	1.00

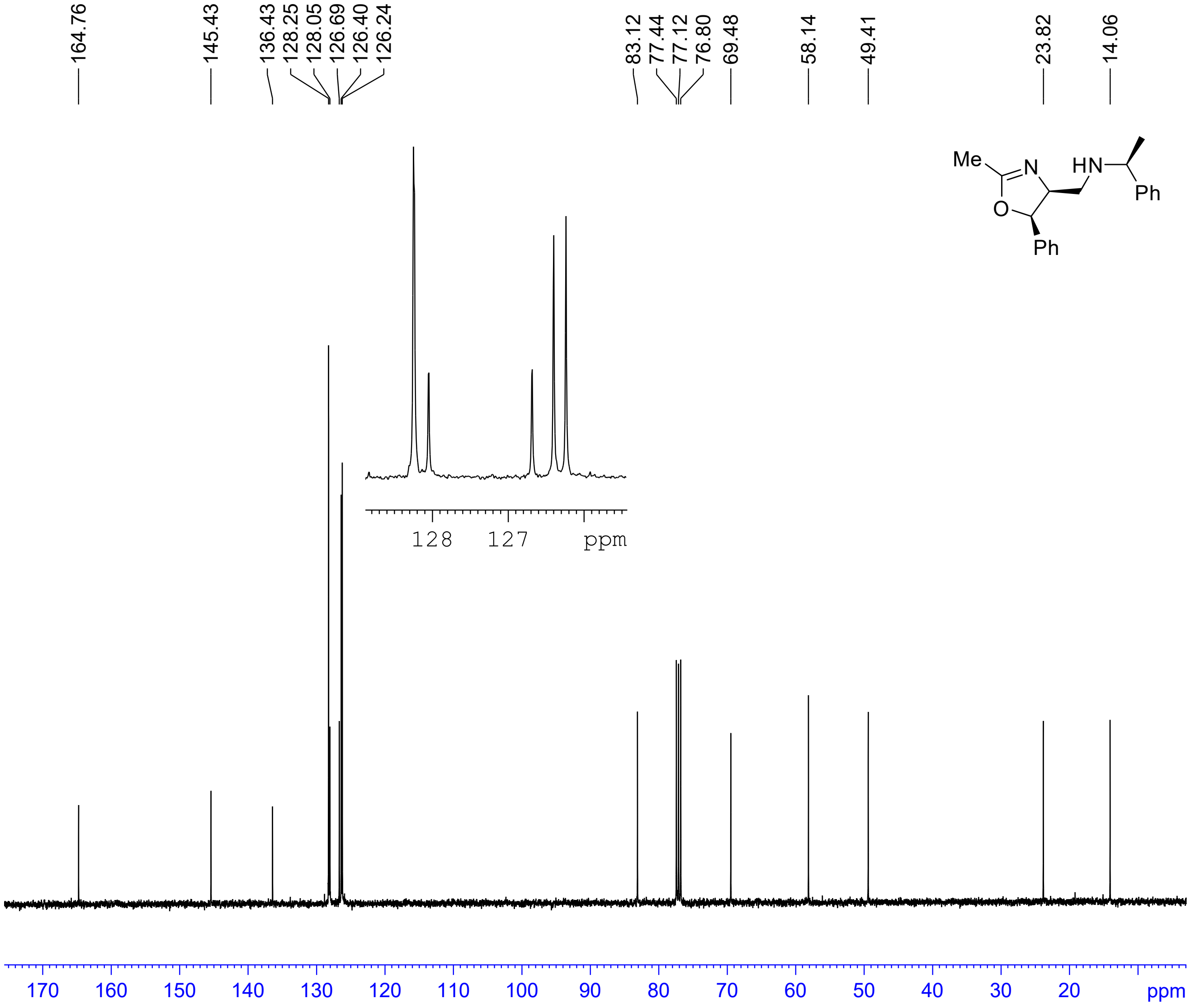

NAME	4o ZX-Me-oxazoline-C13
EXPNO	1
PROCNO	1
Date_	20150206
Time	15.30
INSTRUM	spect
PROBHD	5 mm PABBO BB-
PULPROG	zgpg30
TD	65536
SOLVENT	CDCl3
NS	73
DS	4
SWH	24038.461 Hz
FIDRES	0.366798 Hz
AQ	1.3631988 sec
RG	203
DW	20.800 usec
DE	6.50 usec
TE	297.4 K
D1	2.00000000 sec
D11	0.03000000 sec
TD0	1
======== CHANNEL f1 ========	
NUC1	13C
P1	8.50 usec
PL1	-2.00 dB
PL1W	57.32743073 W
SFO1	100.6328888 MHz
======== CHANNEL f2 ========	
CPDPRG2	waltz16
NUC2	1H
PCPD2	80.00 usec
PL2	-1.00 dB
PL12	14.26 dB
PL13	14.46 dB
PL2W	13.18669796 W
PL12W	0.39276794 W
PL13W	0.37509048 W
SFO2	400.1716007 MHz
SI	32768
SF	100.6228270 MHz
WDW	EM
SSB	0
LB	1.00 Hz
GB	0
PC	1.40

4o ^{13}C NMR

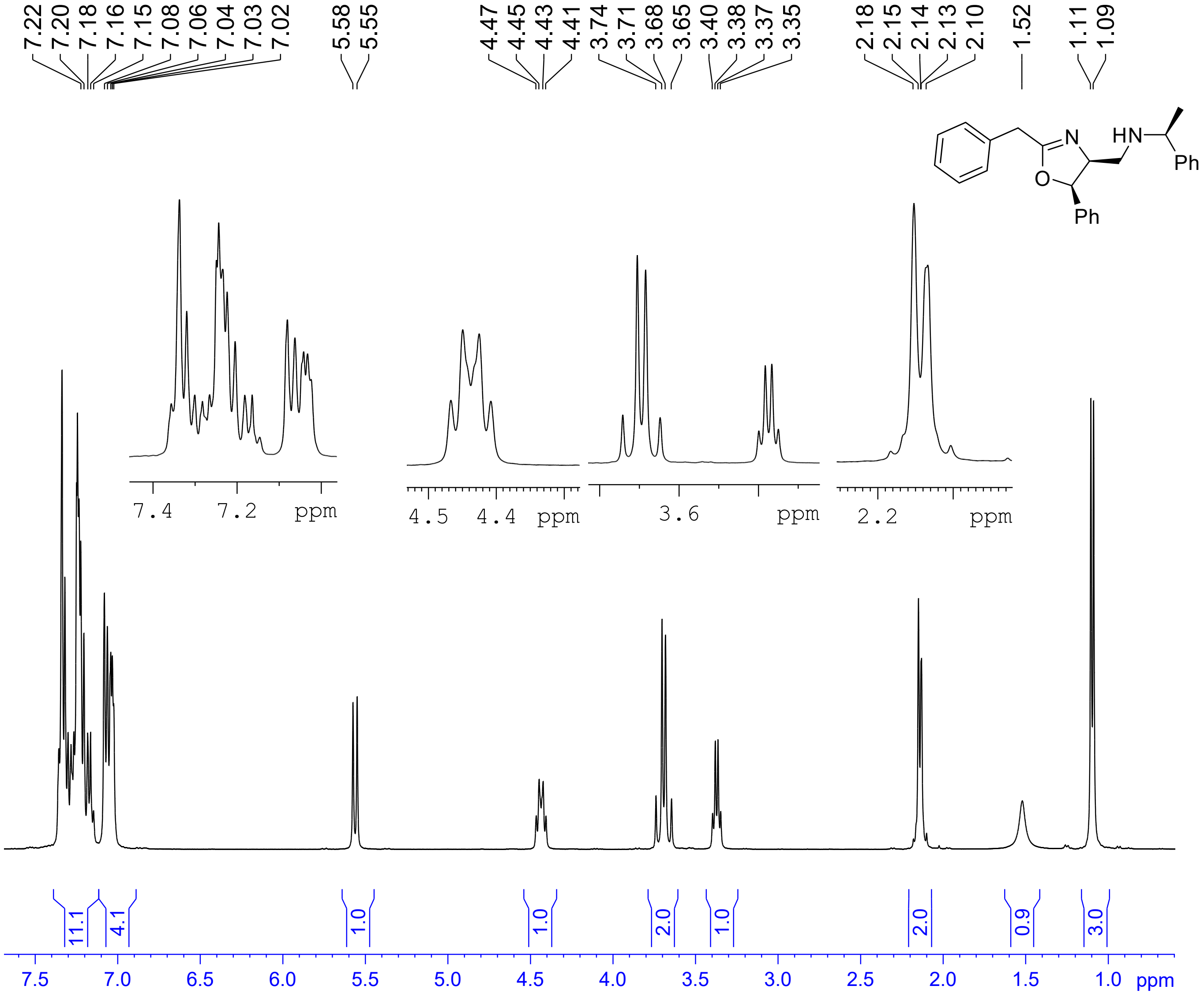

NAME	4p ZX-Bn-oxazoline-6
EXPNO	1
PROCNO	1
Date_	20150309
Time	21.06
INSTRUM	spect
PROBHD	5 mm PABBO BB-
PULPROG	zg30
TD	65536
SOLVENT	CDCl3
NS	16
DS	2
SWH	8223.685 Hz
FIDRES	0.125483 Hz
AQ	3.9846387 sec
RG	57
DW	60.800 usec
DE	6.50 usec
TE	293.2 K
D1	1.00000000 sec
TD0	1

======== CHANNEL f1 ========

NUC1	1H
P1	13.80 usec
PL1	-1.00 dB
PL1W	13.18669796 W
SFO1	400.1724712 MHz
SI	32768
SF	400.1700155 MHz
WDW	EM
SSB	0
LB	0.30 Hz
GB	0
PC	1.00

4p ^{1}H NMR

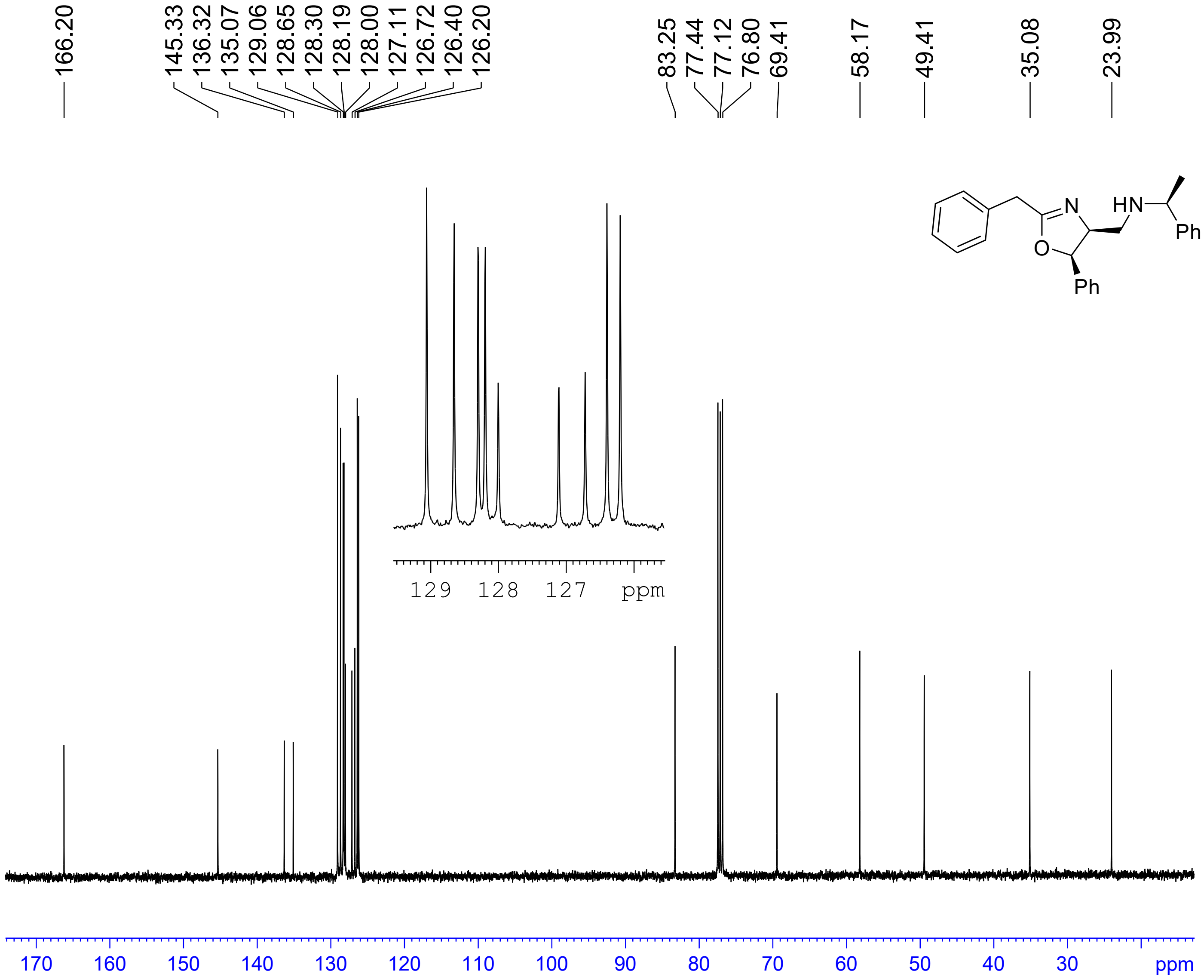

4p ^{13}C NMR

```
NAME       4p ZX-Bn-oxazoline-C13-1
EXPNO                     1
PROCNO                    1
Date_              20150309
Time                  21.09
INSTRUM               spect
PROBHD     5 mm PABBO BB-
PULPROG             zgpg30
TD                    65536
SOLVENT               CDCl3
NS                      143
DS                        4
SWH            24038.461 Hz
FIDRES          0.366798 Hz
AQ             1.3631988 sec
RG                      203
DW               20.800 usec
DE                 6.50 usec
TE                 293.6 K
D1            2.00000000 sec
D11           0.03000000 sec
TD0                       1

======== CHANNEL f1 ========
NUC1                    13C
P1                 8.50 usec
PL1               -2.00 dB
PL1W         57.32743073 W
SFO1         100.6328888 MHz

======== CHANNEL f2 ========
CPDPRG2             waltz16
NUC2                     1H
PCPD2             80.00 usec
PL2               -1.00 dB
PL12              14.26 dB
PL13              14.46 dB
PL2W         13.18669796 W
PL12W         0.39276794 W
PL13W         0.37509048 W
SFO2         400.1716007 MHz
SI                    32768
SF           100.6228270 MHz
WDW                      EM
SSB                       0
LB                 1.00 Hz
GB                        0
PC                     1.40
```
